# Design, Synthesis, and Antitumor Activity of Erlotinib Derivatives

**DOI:** 10.3389/fphar.2022.849364

**Published:** 2022-04-20

**Authors:** Long-fei Mao, Zhen-Zhen Wang, Qiong Wu, Xiaojie Chen, Jian-Xue Yang, Xin Wang, Yue-Ming Li

**Affiliations:** ^1^ State Key Laboratory of Medicinal Chemical Biology, College of Pharmacy and Tianjin Key Laboratory of Molecular Drug Research, Nankai University, Tianjin, China; ^2^ School of Nursing, School of Basic Medical Sciences, Henan University of Science and Technology, Luoyang, China; ^3^ Department of Neurology, The First Affiliated Hospital of Henan University of Science and Technology, Luoyang, China

**Keywords:** erlotinib, EGFR, anticancer, drug-resistant cancer cell lines, 1,2,3-triazole

## Abstract

Nineteen erlotinib derivatives bearing different 1,2,3-triazole moieties were designed, synthesized, and evaluated for their potential against different cancer cell lines. The structures of the synthesized compounds were confirmed *via* ^1^H NMR, ^13^C NMR, and HR MS. Preliminary antitumor activity assay results suggested that some compounds showed remarkable inhibitory activity against different cancer cell lines including the corresponding drug-resistant ones. Among these compounds, 3d was the most promising one with an IC_50_ of 7.17 ± 0.73 μM (KYSE70TR), 7.91 ± 0.61 μM (KYSE410TR), 10.02 ± 0.75 μM (KYSE450TR), 5.76 ± 0.3 3 μM (H1650TR), and 2.38 ± 0.17 μM (HCC827GR). A preliminary mechanism study suggested that compound 3d suppressed cancer cell proliferation through the EGFR-TK pathway.

## Introduction

Epidermal growth factor receptor (EGFR) is closely related to carcinogenesis of different cancers (Wells, 1989; Mendelsohn, 1992; Brand et al., 2006). Many important cellular functions such as cell growth, proliferation, and cell death can be controlled by epidermal growth factor receptor tyrosine kinase (EGFR-TK) (Thomas, 2003). Uncontrollable cell growth and malignant cell proliferation can occur upon the overexpression of EGFR-TK (Murtuza et al., 2019), and inhibiting the high expression of EGFR-TK has been proven to be an effective measure to reduce tumor growth and proliferation (Roskoski, 2014). As a consequence, targeting EGFR has become a prolific field of research such as anticancer (Cohen et al., 2003; Zhang et al., 2017), kidney inflammation and damage (Rayego-Mateos et al., 2018), and anti-inflammation against LPS-stimulated production of NO in peritoneal macrophages (Elkamhawy et al., 2019). Especially, significant progresses were achieved in the treatment of non-small-cell lung cancers using EGFR-targeting therapeutics (Kelloff et al., 1996; Mukherji and Spicer, 2009; Ou, 2012; Liao et al., 2015; Minari et al., 2016; Chen et al., 2019; Wright and Goss, 2019; Rebuzzi et al., 2020). These include different generations of EGFR-TKIs as early as gefitinib (Culy and Faulds, 2002), erlotinib (Kim and Murren, 2002), or the most recent EGFR-TKIs such as rociletinib (Chuang et al., 2016) or osimertinib (Greig, 2016).

While such EGFR-TKIs can significantly improve the life quality and the median survival rate of patients and show better performance in terms of progression-free survival rate or objective response rate, such therapeutics also suffer problems such as drug resistance after a period of administration (Spaans and Goss, 2014; Karlsen et al., 2021; Zhao et al., 2021), and developing new EGFR-TK targeting chemical entities with reduced drug resistance but similar antitumor activity is highly desirable (Zhang et al., 2014; Maione et al., 2015; Tsubata et al., 2021).

We are interested in developing new chemical entities using known therapeutics as lead compounds (Mao LF. et al., 2020; Mao L. et al., 2020; Wang et al., 2020). Herein, we present our preliminary results on the preparation of new erlotinib derivatives, and the antitumor activity of the prepared compounds against different cancer cell lines. Successfully marketed drugs generally showed good druggability such as good pharmacokinetic property, ideal solubility or high activity, and drug discovery process could be effectively facilitated using a known drug as a lead compound.

**FIGURE 1:**
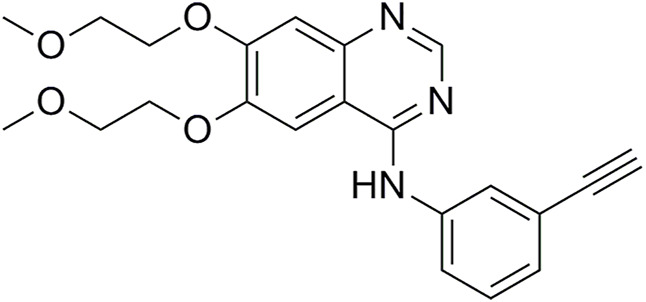
Structure of erlotinib.

Erlotinib (Figure 1) is a classical EGFR-TKI approved for the treatment of advanced non-small-cell lung cancer (NSCLC) (Kim and Murren, 2002; Schettino et al., 2008). Compared with traditional chemotherapeutics, erlotinib can improve the median survival rate of patients and exhibit better performance in terms of progression-free survival rate, objective response rate, quality of life, and tolerability (Mathew et al., 2015).

In addition, erlotinib was also effective in the treatment of esophageal cancer (Choi et al., 2012). Esophageal cancer is a common malignant cancer with high morbidity and mortality, and 5-year survival rate for esophageal cancer patients is lower than that of lung cancer (Hiroshi et al., 2005; Wei et al., 2020). A study showed that 40–80% of esophageal cancer patients are diagnosed with high expression of EGFR (Hirsch et al., 2017), and erlotinib can be used as an adjuvant therapy for the treatment of esophageal cancer in combination with radiotherapy and chemotherapy (Sutter et al., 2005; Zhong et al., 2020). However, drug resistance and adverse reactions have become prominent issues after a period of erlotinib treatment (Sutter et al., 2010; Whitley et al., 2012), and development of new EGFR-TKIs is highly desirable to maintain the antitumor activity of the drug on the one hand, and to tackle the drug resistance problem on the other (Chen et al., 2020).

Nitrogen-containing heterocyclic compounds are widely present in nature and have played key roles in drug discovery (DeSimone et al., 2004; Costantino, 2006). Especially, 1,2,3-triazole moieties are an important category of nitrogen-containing heterocycles in the design of biologically active molecules (Thomopoulou et al., 2015). 1,2,3-Triazole has a large dipole moment and can form a variety of non-covalent interactions with different functional groups. The structural characteristics of 1,2,3-triazoles make them ideal surrogates for amides, esters or carboxylic acids, and compounds bearing triazole moieties often showed broad-spectrum biological activities such as antibacterial (Röhrig et al., 2012; Mao et al., 2017), antimalarial, antifungal, antiviral (Hong et al., 2010), anti-tuberculosis, and antitumor activities (Qi et al., 2020).

**FIGURE 2:**
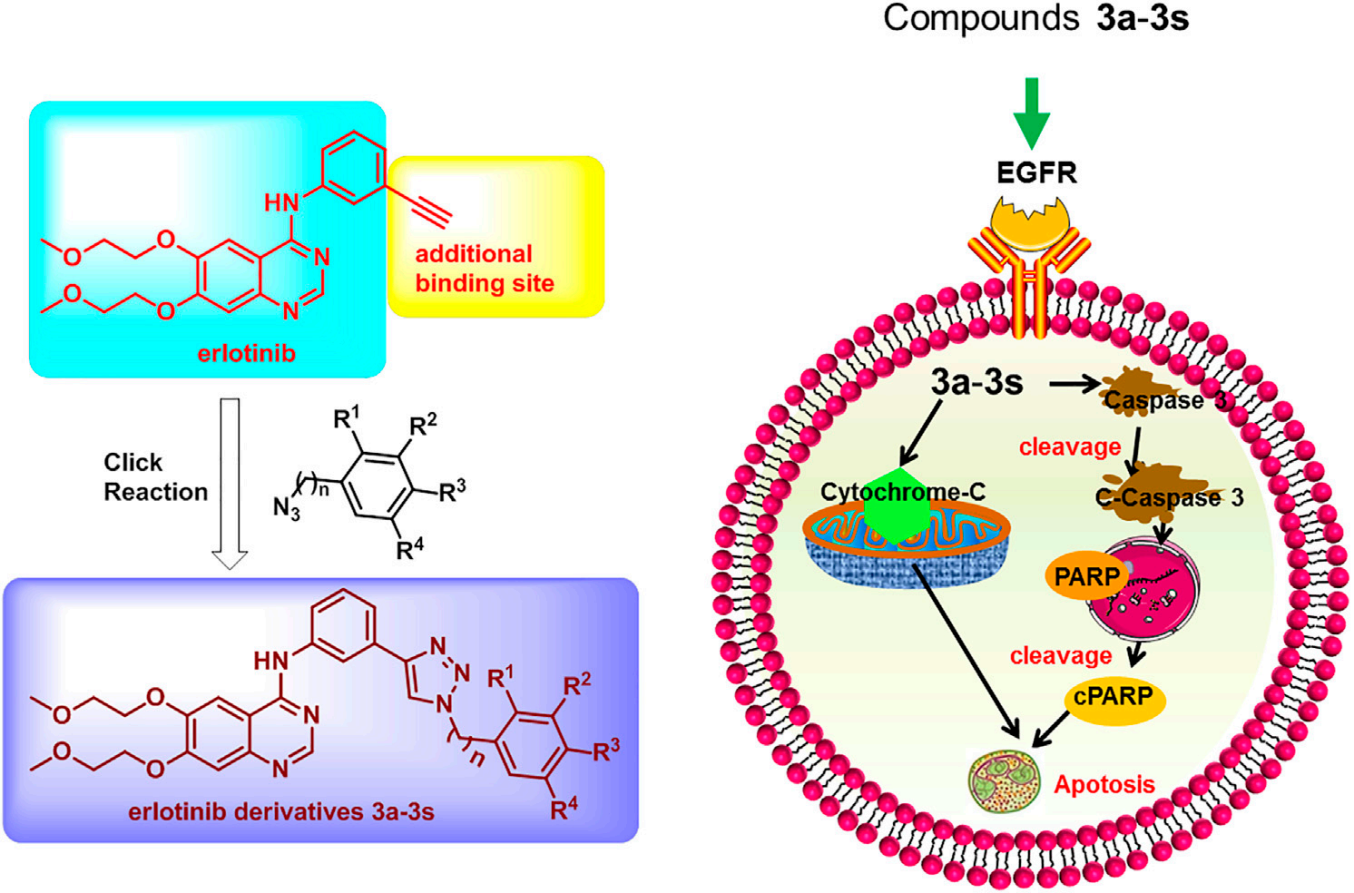
Design strategy of erlotinib derivatives.

On the basis of these rationales, different 1,2,3-triazole moieties were introduced to erlotinib in an attempt to maintaining the antitumor activity of the parent drug and in some extent tackling the drug resistance problem of the drug (Figure 2).

## Results and Discussion

### Chemistry

The preparation of the target compounds is illustrated in Scheme 1. Erlotinib (**2**) was obtained after the reaction of 4-chloro-6,7-bis(methoxyethoxy)quinazolinone with 3-aminophenylacetylene (Chandregowda et al., 2007a, b, c). The target compounds **3a-3s** were obtained *via* the click reaction of erlotinib with different azido compounds (Xu et al., 2016) (Table 1). The reaction conditions of these operations were mild, and the reactions were easy to carry out. The structures of the key intermediates and all target compounds were confirmed by nuclear magnetic resonance (^1^H NMR and ^13^C NMR) and high-resolution mass spectrometry (HR MS). The purity of the key compounds was checked with HPLC.

**TABLE 1:** Structures of compounds **3a-3s**.

Compound no.	n	*R* ^1^	*R* ^2^	*R* ^3^	*R* ^4^
**3a**	1	H	H	H	H
**3b**	1	I	H	H	H
**3c**	1	Br	H	H	H
**3d**	1	H	Br	H	Br
**3e**	1	H	OCH_3_	H	H
**3f**	0	F	H	H	H
**3g**	0	H	H	F	H
**3h**	0	Cl	H	H	H
**3i**	0	Br	H	H	H
**3j**	0	H	H	Br	H
**3k**	0	OCH_3_	H	H	H
**3l**	0	H	H	CH_3_	H
**3m**	0	H	NO_2_	H	H
**3n**	0	H	OCH_2_CH_3_	H	H
**3o**	0	H	H	H	H
**3p**	0	CF_3_	H	H	H
**3q**	0	OCH_3_	H	OCH_3_	H
**3r**	0	OH	H	CH_3_	H
**3s**	2	H	H	H	H

### Suppression of Esophageal Cancer Cell Lines and Non-Small-Cell Lung Cancer Cell Lines by Erlotinib–1,2,3-Triazole Derivatives

A total of five tumor cell lines including three esophageal cancer cell lines (KYSE70, KYSE410, and KYSE450) and two lung cancer cell lines (H1650 and HCC827) were chosen for evaluating the anticancer activity of the newly synthesized erlotinib derivatives. Three types of esophageal cancer cell lines KYSE70TR, KYSE410TR, and KYSE450TR which were resistant to paclitaxel, and NSCLC cell lines HCC827GR and H1650TR, which were resistant to gefitinib and paclitaxel, respectively, were also tested for the comparison purpose. The resistance indices of these cell lines are shown in Table 2.

**TABLE 2:** Resistance index of the five drug resistance cell lines.

Cell	24 h IC_50_ (nM/L)	Resistance index	48 h IC_50_ (nM/L)	Resistance index
KYSE 70	640.07 ± 43.03	7.69	20.21 ± 219	56
KYSE 70TR	4924.27 ± 274.11		1120.33 ± 35.16	
KYSE 410	1280.09 ± 69.27	5	52 ± 3.45	32.88
KYSE 410TR	6400.43 ± 430.15		1710.05 ± 86.42	
KYSE 450	55.03 ± 0.33	4.84	7.82 ± 0.84	15.38
KYSE 450TR	266.13 ± 66.14		120.32 ± 32.14	
H1650	870.07 ± 43.52	4.74	83.33 ± 3.33	7.74
H1650TR	4126.12 ± 207.65		645.03 ± 45.03	
HCC827	10.33 ± 0.33	4.26	21.39 ± 1.39	5.61
HCC827GR	44.11 ± 2.42		120.25 ± 25.82	

The results were from statistical analysis and were presented with mean ± SD.

At first, MTT experiments were carried out to study the cytotoxicity of **3a**-**3s** against five cell lines using erlotinib as a control. The results expressed in IC_50_ values are shown in Table 3. KYSE410 cells were most sensitive to erlotinib with an IC_50_ value of 5.00 ± 0.46 μM. KYSE450 cells and two lung cancer cell lines (H1650 and HCC827) also showed some effects following erlotinib treatment, with IC_50_ values of 7.60 ± 0.51 μM, 14.00 ± 1.19 μM, and 11.81 ± 1.02 μM, respectively. The KYSE70 cell line was not very sensitive to erlotinib, but some erlotinib 1,2,3-triazole derivatives had good inhibitory activities against KYSE70 cells. For example, compounds **3a**, **3b**, **3c**, **3d**, **3g**, **3j**, **3m**, and **3r** showed IC_50_ values less than 10 μM, which were 5.85 ± 0.28 μM, 9.03 ± 0.82 μM, 5.35 ± 0.34 μM, 5.43 ± 0.21 μM, 5.46 ± 0.23 μM, 3.72 ± 0.33 μM, 3.92 ± 0.15 μM, and 5.85 ± 0.52 μM, respectively. Compounds with IC_50_ values less than 10 μM against KYSE410 cells included **3a** (9.70 ± 0.75 μM), **3d** (6.91 ± 0.40 μM), **3m** (5.85 ± 0.20 μM), and **3r** (8.74 ± 0.74 μM). Compounds with IC_50_ values less than 10 μM against KYSE450 cells included **3a** (5.27 ± 0.31 μM), **3c** (5.47 ± 0.29 μM), **3d** (4.23 ± 0.19 μM), **3e** (6.25 ± 0.35 μM), **3j** (3.60 ± 0.27 μM), **3m** (4.05 ± 0.21 μM), and **3r** (8.20 ± 0.67 μM). Compounds with IC_50_ values less than 10 μM against H1650 cells included **3c** (5.72 ± 0.33 μM), **3d** (2.99 ± 0.13 μM), **3j** (4.98 ± 0.17 μM), and **3m** (4.27 ± 0.19 μM). Compounds with IC_50_ values less than 10 μM against HCC827 cells included **3d** (8.17 ± 0.42 μM), **3j** (6.27 ± 0.42 μM), **3k** (6.18 ± 0.61 μM), **3l** (4.61 ± 0.28 μM), and **3m** (8.44 ± 0.37 μM). These preliminary results suggested that compounds **3d** and **3m** showed good inhibitory activities against these tumor cell lines with IC_50_ values less than 10 μM. Compound **3d** was more suitable for further study due to the easy availability of the key azide raw material. The human esophageal epithelial cell, namely SHEE, was chosen for MTT assay to evaluate the toxicity of the compound against normal cells. As shown in Table 2, SHEE showed poor sensitivity to **3a**, **3d**, **3e**, **3m**, **3p**, and **3r**, with IC_50_ values of over 50 μM. These preliminary results suggested that compounds **3d**, **3m**, and **3r** may be used as lead compounds for the development of chemotherapeutic agents for esophageal cancer. Compound **3d** was chosen for further study due to its good performance and easier preparation.

**TABLE 3:** Anti-proliferative activities of compounds **3a-3s** against different cell lines.

Compound no.	IC_50_(μM)					
	KYSE70	KYSE410	KYSE450	H1650	HCC827	SHEE
**3a**	5.85 ± 0.28	9.70 ± 0.75	5.27 ± 0.31	33.96 ± 2.77	>50	>50
**3b**	9.03 ± 0.82	17.55 ± 1.37	20.57 ± 1.87	>50	>50	8.05 ± 0.71
**3c**	5.35 ± 0.34	>50	5.47 ± 0.29	5.72 ± 0.33	>50	22.35 ± 2.53
**3d**	5.43 ± 0.21	6.91 ± 0.40	4.23 ± 0.19	2.99 ± 0.13	8.17 ± 0.42	>50
**3e**	12.60 ± 1.01	>50	6.25 ± 0.35	21.10 ± 1.74	>50	>50
**3f**	>50	>50	34.20 ± 3.01	>50	17.81 ± 1.61	12.14 ± 1.33
**3g**	5.46 ± 0.23	>50	44.46 ± 4.23	29.58 ± 2.18	>50	8.01 ± 0.83
**3h**	>50	>50	>50	>50	>50	6.03 ± 0.57
**3i**	36.08 ± 3.17	>50	>50	>50	24.23 ± 2.34	6.23 ± 0.62
**3j**	3.72 ± 0.33	>50	3.60 ± 0.27	4.98 ± 0.17	6.27 ± 0.42	7.2 ± 0.63
**3k**	16.0 ± 1.53	32.00 ± 3.25	20.00 ± 2.25	16.02 ± 1.69	6.18 ± 0.61	10.33 ± 0.96
**3l**	11.11 ± 0.94	>50	>50	10.02 ± 0.79	4.61 ± 0.28	4.82 ± 0.32
**3m**	3.92 ± 0.15	5.85 ± 0.20	4.05 ± 0.21	4.27 ± 0.19	8.44 ± 0.37	>50
**3n**	>50	49.70 ± 4.57	12.76 ± 1.25	35.35 ± 3.46	13.71 ± 1.34	22.59 ± 2.31
**3o**	>50	49.5 ± 5.01	46.76 ± 4.63	>50	>50	14.17 ± 1.61
**3p**	>50	>50	24.90 ± 2.16	>50	34.67	>50
**3q**	>50	12.27 ± 0.93	>50	>50	>50	28.08 ± 2.47
**3r**	5.85 ± 0.52	8.74 ± 0.74	8.20 ± 0.67	4.32 ± 0.27	12.43 ± 1.07	>50
**3s**	>50	>50	>50	>50	>50	33.26 ± 3.43
**Erlotinib**	>50	5.00 ± 0.46	7.60 ± 0.51	14.00 ± 1.19	11.81 ± 1.02	20.99 ± 2.11

Conditions: Growth inhibition was evaluated with MTT assay. The absorbance at 490 nm was measured using a microplate reader (Thermo). The results were from statistical analysis and were presented as mean ± SD.

Next, **3d** and erlotinib were tested for their activity against three drug-resistant esophageal cancer cell lines (KYSE70TR, KYSE410TR, and KYSE450TR) and two drug-resistant lung cancer cell lines (H1650TR and HCC827GR). The results expressed by IC_50_ values are shown in Table 4. The preliminary results suggested that the inhibitory effect of erlotinib on drug-resistant tumor cells was not significant and the IC_50_ values were over 10 μM for all cases. In contrast, **3d** showed good inhibitory effect on five drug-resistant tumor cell lines with IC_50_ values of 7.17 ± 0.73 μM, 7.91 ± 0.61 μM, 10.02 ± 0.75 μM, 5.76 ± 0.33 μM, and 2.38 ± 0.17 μM, respectively.

**TABLE 4:** Anti-proliferative activities of compounds **3d** and erlotinib against drug-resistant cancer cell lines.

	IC_50_(μM)				
	KYSE70TR	KYSE410TR	KYSE450TR	H1650TR	HCC827GR
**3d**	7.17 ± 0.73	7.91 ± 0.61	10.02 ± 0.75	5.76 ± 0.33	2.38 ± 0.17
Erlotinib	>20	>20	>20	>20	>20

### Plate Clone Formation Assay

**FIGURE 3:**
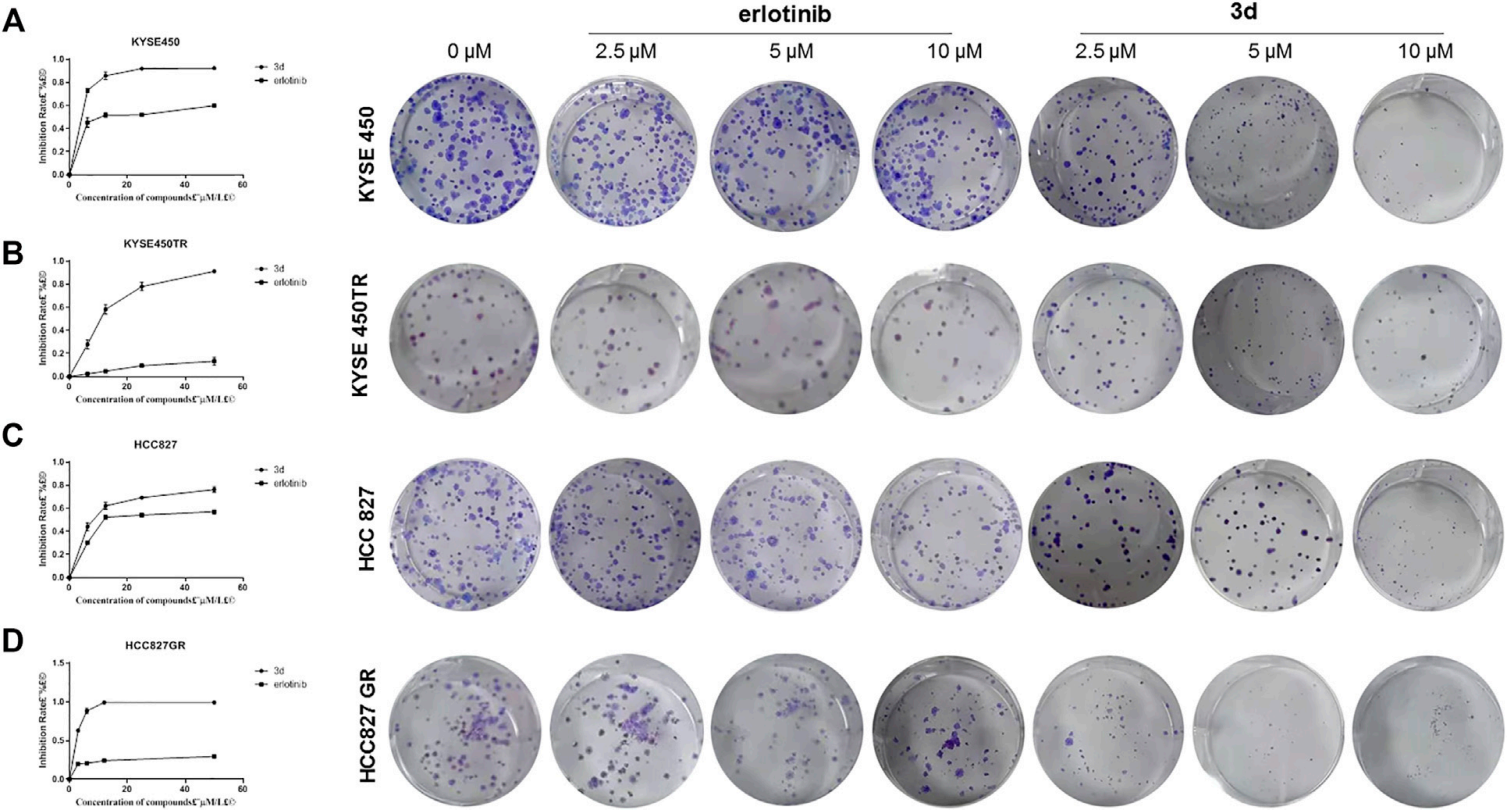
Plate clone results of **3d** and erlotinib. **(A)** and **(B)** The proliferation inhibitionof **3d** and erlotinib on KYSE450 and KYSE450TRcells. **(C)** and **(D)** The proliferation inhibitionof **3d** and erlotinibon HCC827 and HCC827GRcells. Unpaired Student’s t test was used in Plate clone. *p< 0.05, **p< 0.01, ***p< 0.001. Error bars represent the mean ± SD.

Plate clone experiments of **3d** and erlotinib were also carried out to study the tumor response (Von Hoff et al., 1980), and the results similar to MTT experiments were observed (Figure 3). The inhibition effects of **3d** on both cancer cell lines and the corresponding drug-resistant ones were more significant than that of erlotinib.

### Apoptosis in Esophageal Cancer Cell Lines Induced by Erlotinib–1,2,3-Triazole Derivatives

**FIGURE 4:**
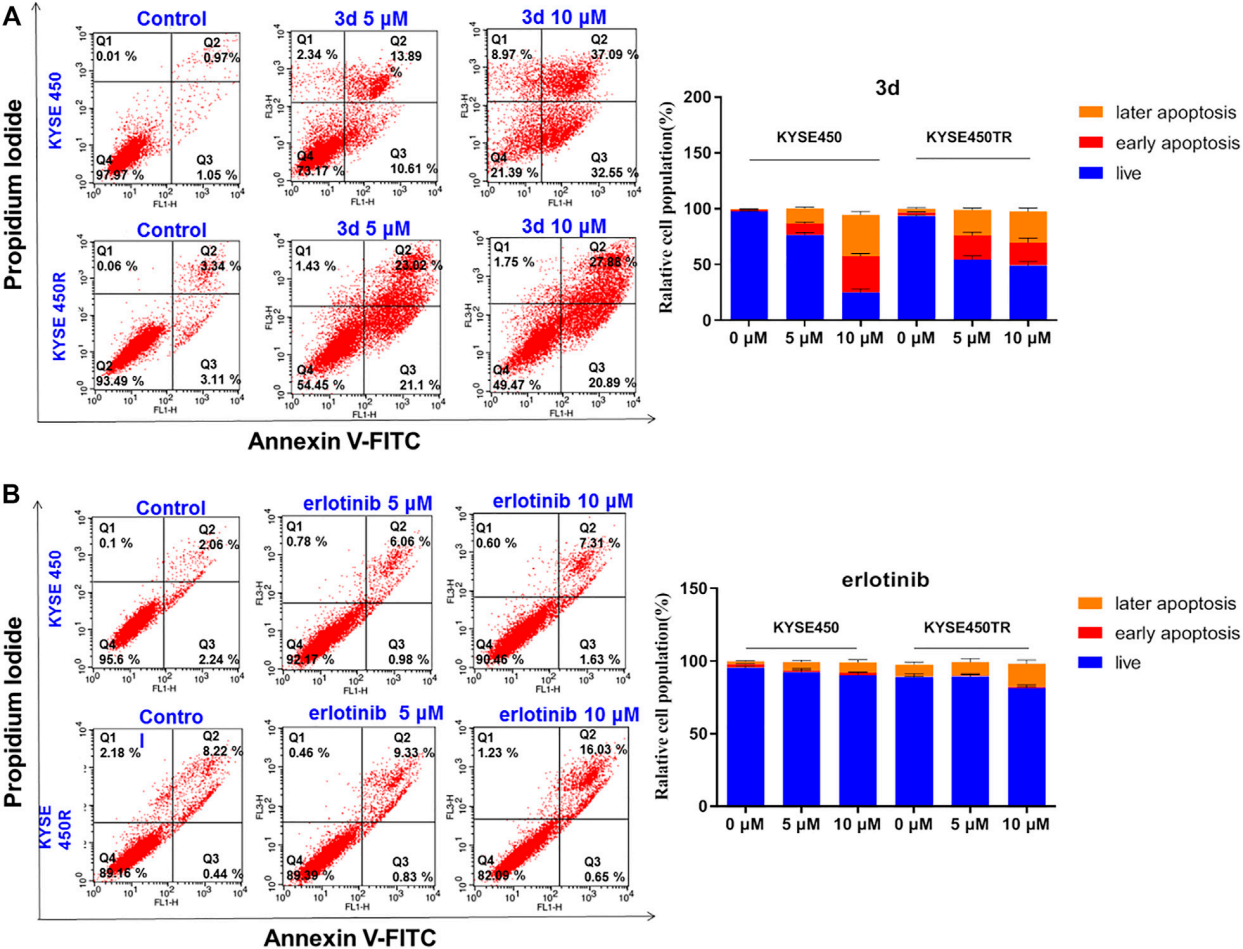
Cell apoptosis induced by compound **3d** and erlotinib. **(A)** Cell apoptosis induced by compound **3d** in KYSE450 cell and KYSE450TR cell, compared with cells treated with 0.1% DMSO; **(B)** Cell apoptosis induced by erlotinib in KYSE450 cell and KYSE450TR cell,compared with cells treated with 0.1% DMSO. Unpaired Student’s t test was used inCell apoptosis. *p< 0.05, **p< 0.01, **p< 0.001. Error bars represent the mean ± SD.

To clarify whether the inhibitory effects of these compounds on cell proliferation were related to apoptosis, compound **3d**, which showed strong inhibitory effects on the proliferation of esophageal cancer cell lines, were chosen for further study. KYSE450 and KYSE450T cells were treated with DMSO or different concentrations of **3d** and erlotinib for 48 h, the cells were stained with Annexin V and PI, and the proportion of apoptotic cells was detected with flow cytometry. The results are shown in Figure 4.

Results in Figure 4A showed that the proportion of KYSE450 apoptotic cells treated with **3d** was 24.50% (5 μM) and 69.64% (10 μM), while the proportion of KYSE450TR apoptotic cells treated with **3d** was 44.12% (5 μM) and 48.77% (10 μM). Results in Figure 4B showed that the proportion of KYSE450 apoptotic cells treated with erlotinib was 7.04% (5 μM) and 8.94% (10 μM), while the proportion of KYSE450TR apoptotic cells treated with erlotinib was 10.16% (5 μM) and 16.68% (10 μM). These preliminary results suggested that compound **3d** could induce apoptosis of esophageal cancer KYSE450 cells and drug-resistant KYSE450TR cells in a concentration-dependent manner, and the performance of compound **3d** on these two cells was better than that of erlotinib.

### Erlotinib–1,2,3-Triazole Derivatives Trigger Apoptosis Through the Mitochondrial Pathway

**FIGURE 5:**
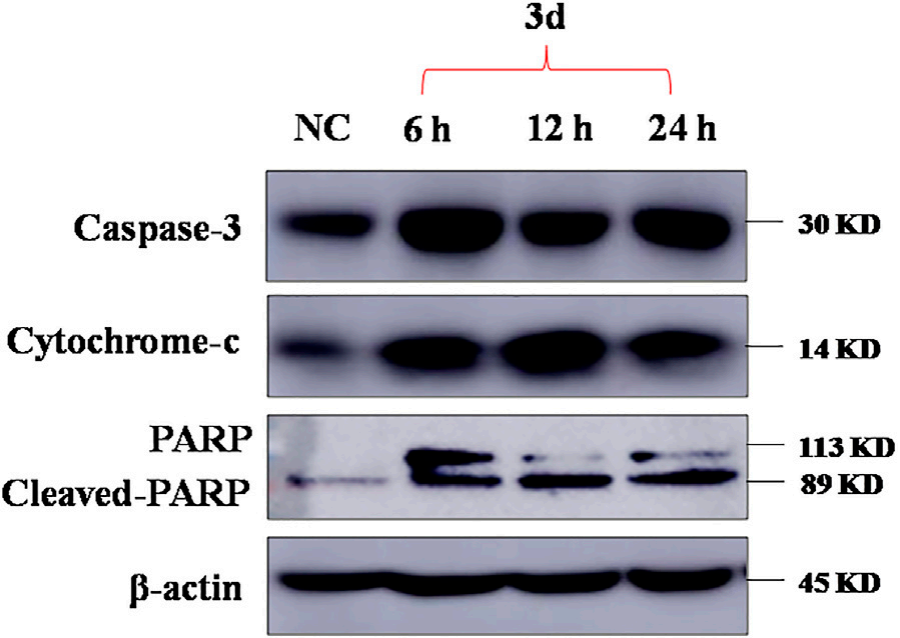
Apoptosis-associated protein changes induced by **3d** in KYSE450.

To investigate whether the mechanism of **3d**-induced KYSE450 cell apoptosis was related to mitochondrial apoptosis, protein electrophoresis was carried out to measure the protein levels of apoptosis-related marker proteins caspase-3, cytochrome-c, and PARP. The results are presented in Figure 5.

KYSE450 cells were treated with **3d** for 6, 12, and 24 h, respectively. Analysis of total cell proteins showed that in KYSE450 cells, the caspase-3 and cytochrome-c protein levels of **3d** (4 μM) group were higher than those in the control at 0 h after administration. The results suggest that **3d** can regulate KYSE450 cell apoptosis through the mitochondrial pathway. The cleaved PARP protein levels of the **3d** group were higher than those of the control at 0 h after administration, suggesting that compound **3d** may regulate KYSE450 apoptosis through DNA injury.

### Erlotinib–1,2,3-Triazole Derivatives Induce Esophageal Cells Death *via* Arresting Cell Cycle

**FIGURE 6:**
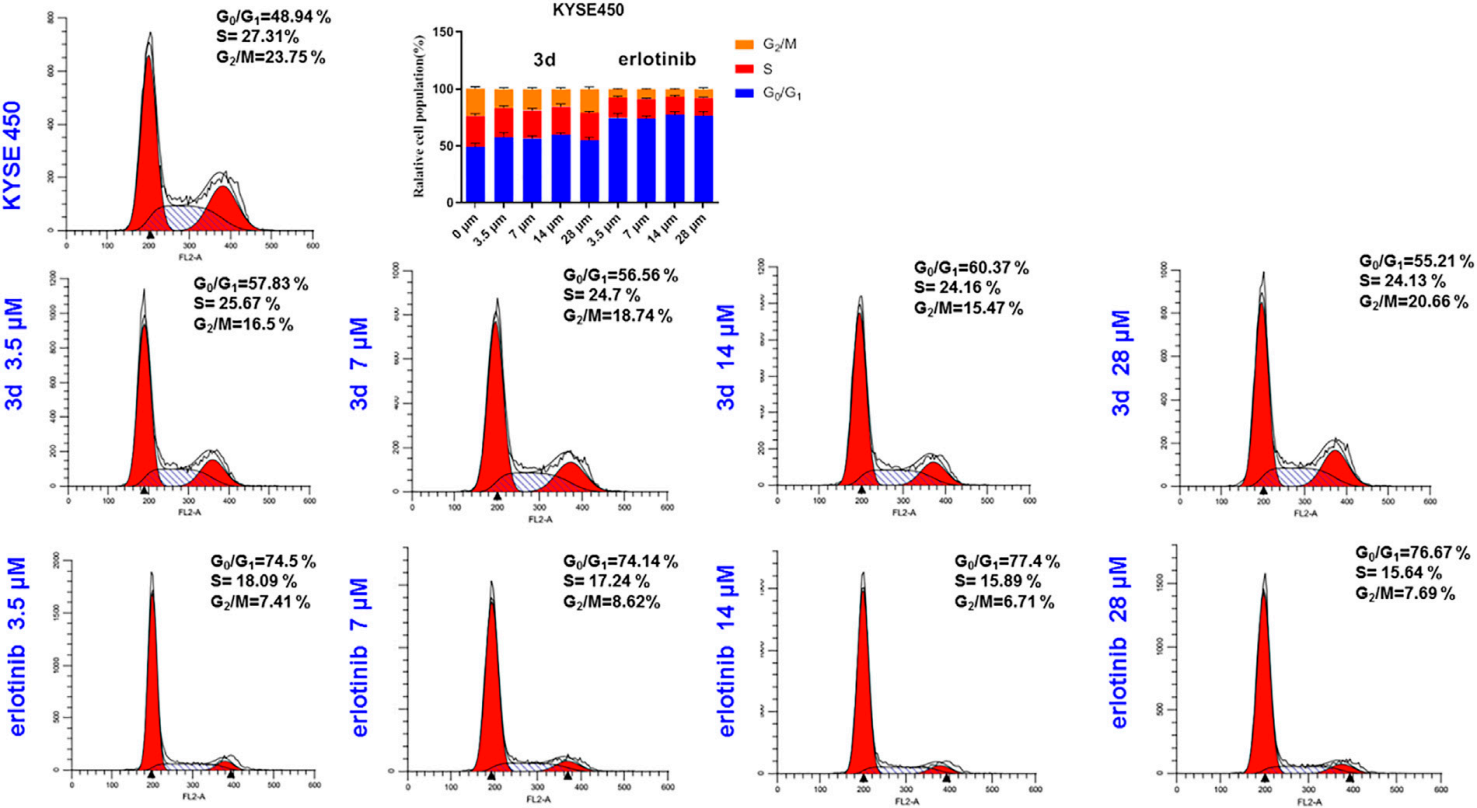
KYSE450 cell cycle arrests induced by compound **3d** and erlotinib in comparison with the cells treated with 0.1% DMSO.

To assess whether the inhibitory effect of these compounds on the proliferation of esophageal cancer cells was related to cell cycle arrest, cancer cells were treated with DMSO or different concentrations of **3d** and erlotinib. Then, the cell cycle phases were evaluated with flow cytometry (Figure 6). In comparison with the control, the **3d** group showed that the ratio of KYSE450 cells in the G_0_/G_1_ phase increased after 24 h with the increase in concentration (3.5, 7, 14, and 28 μM) in comparison with the control. However, the change in the ratio of S-phase cells and the ratio of G_2_/M-phase cells were not remarkable. When the cells were treated with erlotinib, the ratio of KYSE450 cells in the G_0_/G_1_ phase increased significantly at 3.5 μM and increased further with the concentration (3.5, 7, 14, and 28 μM) but did not change significantly with the further increase in concentration. The ratio of S-phase cells and the ratio of G_2_/M-phase cells decreased significantly at 3.5 μM but did not change significantly when the concentration was further increased. Therefore, both compound **3d** and erlotinib inhibited KYSE450 cells in the G_0_/G_1_ phase.

**FIGURE 7:**
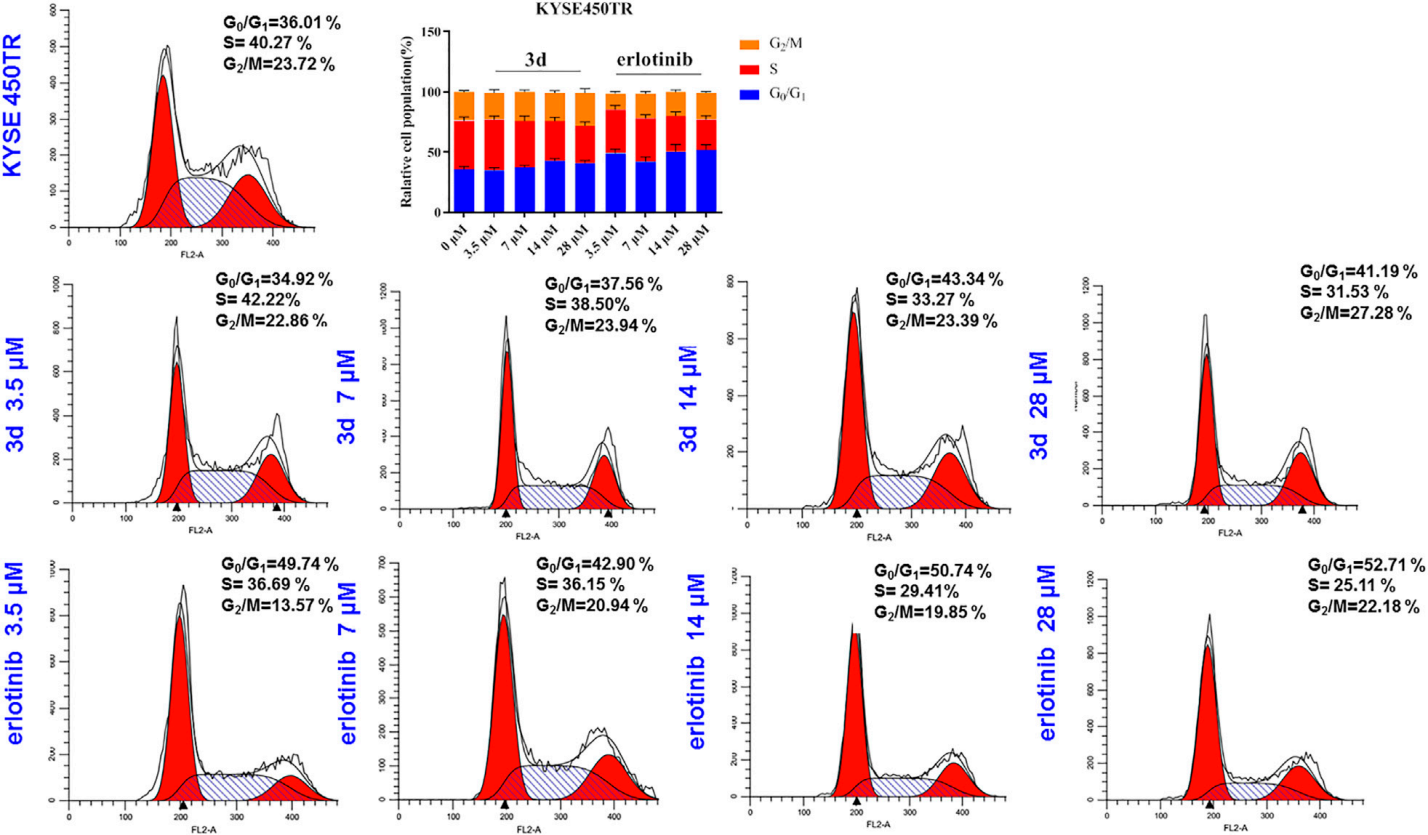
Cell cycle arrests induced by compound **3d** and erlotinib in KYSE450TR, compared with the cells treated with 0.1% DMSO.

For the drug resistance cell line KYSE450TR, the cell cycle results showed that the ratio of KYSE450TR cells in the G_0_/G_1_ phase increased after treatment with compound **3d** for 24 h with the increase in concentration (3.5, 7, 14, and 28 μM) in comparison with the control (Figure 7). However, the ratio of S-phase cells significantly reduced, and the ratio of G_2_/M-phase cells increased in a concentration-dependent manner. When cells were treated with erlotinib, the ratio of KYSE450TR cells in the G_0_/G_1_ phase increased significantly at 3.5 μM and further increased with concentration (3.5, 7, 14, and 28 μM) but did not change significantly when the concentration was further increased. The ratio of S-phase cells decreased significantly at 3.5 μM, and the change was significant with the increase in concentration. The ratio of cells in the G_2_/M phase increased to a small degree with the increase in concentration. Therefore, compound **3d** inhibited KYSE450TR cells in the G_2_/M phase, whereas erlotinib arrested KYSE450TR cells in the G_0_/G_1_ phase.

### Erlotinib–1,2,3-Triazole Derivatives Suppress Cancer Cell Proliferation Through EGFR-TK Pathway

**FIGURE 8:**
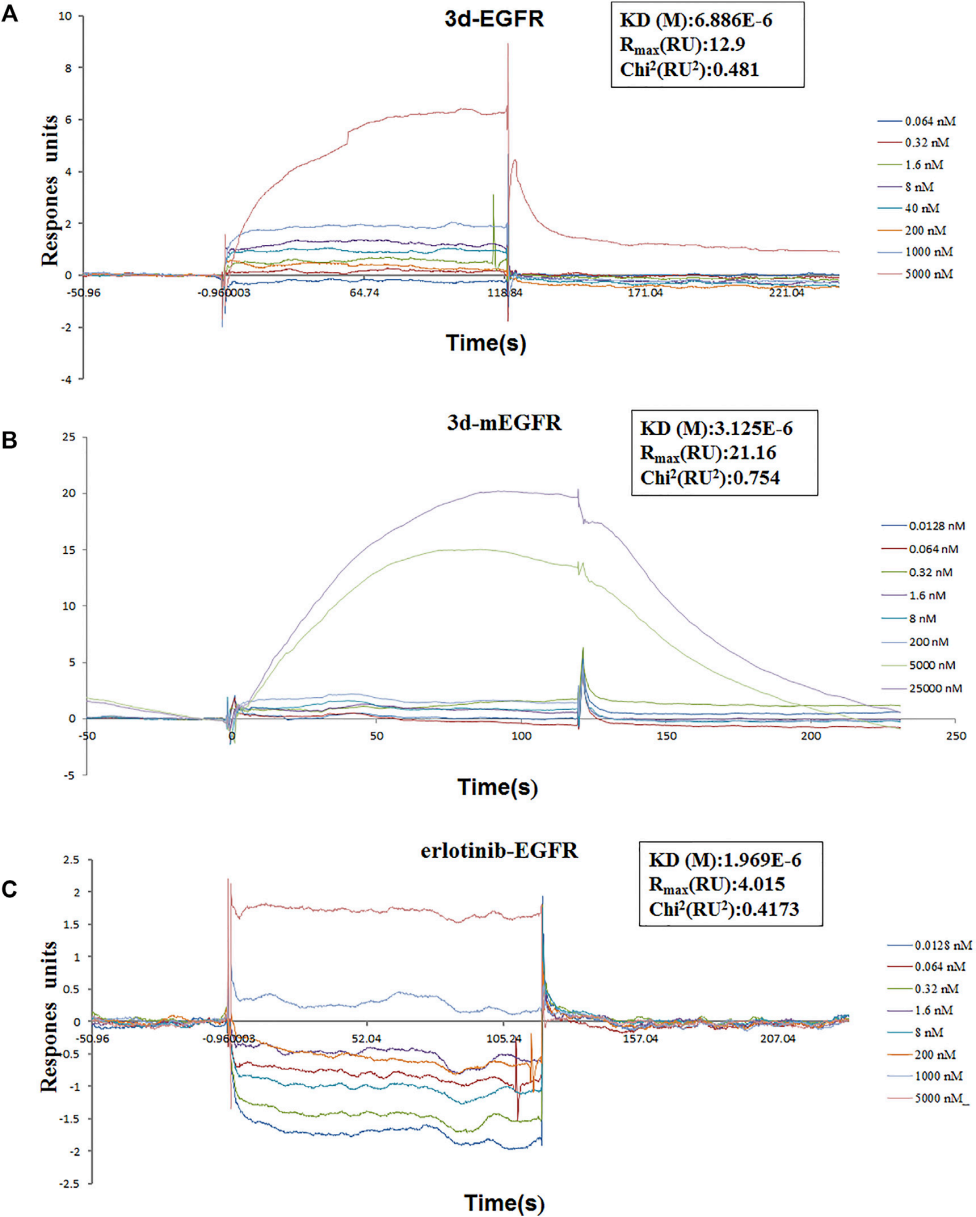
Surface plasmon resonance experiments of **3d** and erlotinib. **(A)** Binding sensorgrams for **3d** interaction with immobilized wild-type of EGFR. The K_D_(M) value between **3d** and wild-type EGFR is 6.88 × 10^−6^. **(B)** Binding sensorgrams for **3d** interaction with immobilized mutant protein of EGFR. The K_D_(M) value between **3d** and mutant protein EGFR is 3.12 × 10^−6^. **(C)** Binding sensorgrams for erlotinib interaction with immobilized wild-type of EGFR. The K_D_(M) value between erlotinib and wild-type EGFR is 1.97 × 10^−6^.

To investigate whether the mechanism of **3d** suppressing cancer cell proliferation was related to the EGFR-TK pathway, surface plasmon resonance (SPR) experiments were carried out to study the interaction between **3d** and erlotinib with the EGFR. The results are presented in Figure 8. As these results showed, **3d** can bind to the EGFR wild-type protein (H672-E410) and EGFR mutant protein (672-1210, L858R) with a *K*_D_(M) value of 6.88 × 10^−6^ and 3.12 × 10^−6^, respectively. The *K*_D_(M) value for erlotinib and EGFR was 1.97 × 10^−6^. These preliminary results also suggested that **3d** suppressed cancer cells proliferation through the EGFR-TK pathway.

## Conclusion

In summary, erlotinib derivatives bearing 1,2,3-triazole moieties were designed, synthesized, and evaluated for their activity against different cancer cell lines. Several of these compounds exhibited remarkable antitumor activity than erlotinib against one or more cancer cell lines. Among these compounds, **3d** demonstrated good cytotoxicity against all ten cancer cell lines. The underlying mechanisms of **3d**-induced cancer cell deaths were mitochondrial apoptosis and cell cycle arrest. In addition, a preliminary study on the interaction between **3d** and EGFR suggested that **3d** can bind to both wild-type protein (H672-E410) and mutant protein (672-1210, L858R). Taking these results together, erlotinib–1,2,3-triazole derivative **3d** could induce apoptosis and arrest cell cycle, and the combination of erlotinib and 1,2,3-triazole might be a successful strategy for the development of new EGFR inhibitors for cancer therapy.

## Experimental Protocols

### Chemistry

All reagents and solvents were obtained from commercially available sources and were used as received. ^1^H NMR and ^13^C NMR spectra were acquired in DMSO-d_6_ solution using a Bruker 600 spectrometer. Chemical shifts (δ) were given in parts per million with tetramethylsilane as internal reference. Coupling constants were expressed in hertz. High-resolution mass spectra (HRMS) measurements were carried out using a Bruker MicrOTOF-Q II mass spectrometer.

### Preparation of 3a-3s

#### Preparation of Erlotinib

Compound **2** (3 g, 0.01 mol) was suspended in isopropanol alcohol (50 ml). 3-Aminophenylacetylene (1.2 g, 0.01 mol) was added to the solution. The suspension was stirred at 85°C for 6 h under nitrogen. Solid gradually formed, and the course of the reaction was monitored with TLC. After the completion of the reaction, the reaction mixture was transferred to ice water, and the mixture was stirred for half an hour. The solid was collected by filtration and was washed twice with isopropanol (30 ml) to give 2.1 g of erlotinib. ^1^H NMR (600 MHz, DMSO-d_6_): *δ* 9.48 (s, 1H, NH), 8.51 (s, 1H, CH), 8.00 (s, 1H, Ar-H), 7.91 (d, J = 9.5 Hz, 1H, Ar-H), 7.87 (s, 1H, Ar-H), 7.41 (t, J = 7.9 Hz, 1H, Ar-H), 7.27–7.17 (m, 2H, Ar-H), 4.31–4.29 (m, 4H, CH_2_CH_2_), 4.21 (s, 1H, CH), 3.80–3.75 (m, 4H, CH_2_CH_2_), 3.38 (s, 3H, CH_3_), 3.36 (s, 3H, CH_3_); ^13^C NMR (150 MHz, DMSO-*d*_*6*_): 156.6, 154.1, 153.2, 148.6, 147.4, 140.2, 129.3, 126.8, 125.2, 123.0, 122.2, 109.3, 108.6, 103.6, 83.9, 81.0, 70.5, 70.5, 68.8, 68.5, 58.8, and 58.8; HRMS (ESI)*m/z*: calcd for C_22_H_23_O_4_N_3_Na (M + Na)^+^ 416.1581, found 416.1585.

#### General Procedure for Preparation of Compounds 3a-3s

**FIGURE 9:**
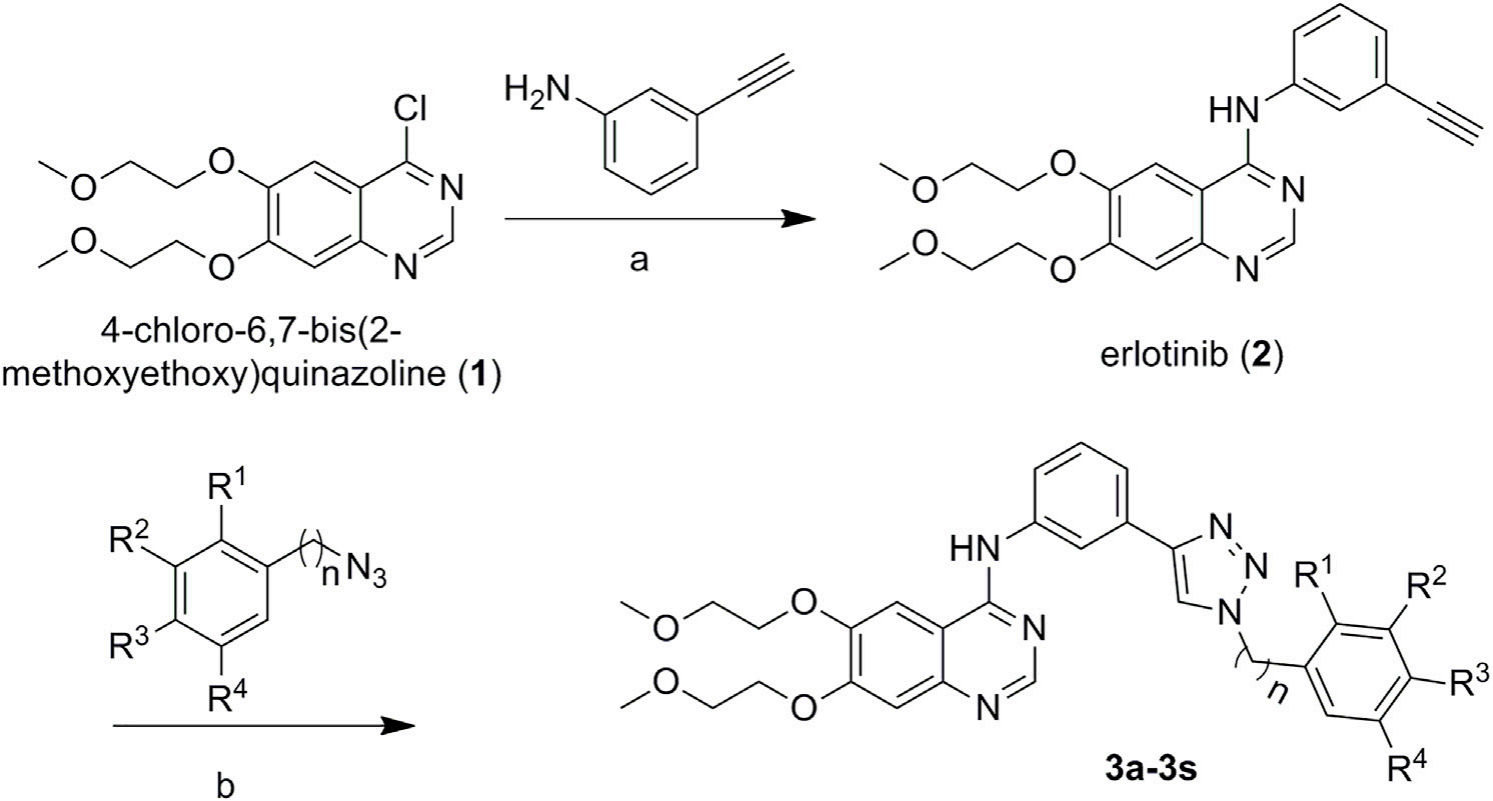
Synthetic routes to erlotinib–1,2,3-triazole derivatives. Conditions: **(A)** isopropanol alcohol, 85°C for 6 h, and **(B)** CuI, 80°C.

Aryl azide (1.2 mmol) and erlotinib (1.0 mmol) were added to 30 ml of mixed solvent (water: *t*-butanol = 2:1). The reaction was carried out in the presence of cuprous iodide (0.1 mmol) at 80°C. After completion of the reaction (monitored by TLC), the mixture was extracted with dichloromethane (20 ml × 3). The combined organic phase was washed successively with water and brine, dried over sodium sulfate, and concentrated *in vacuo*. The residue was purified through column chromatography (VCH_2_Cl_2_/V_MeOH_ = 30:1) to give the desired **3a**-**3s** (Figure 9).

##### {3-[1-Benzyl-1H-(1,2,3)Triazol-4-yl]-Phenyl}-[6,7-Bis-(2-Methoxy-Ethoxy)-Quinazolin-4-yl]-Amine (3a)

Purity 99%; m. p. 89–92°C; ^1^H NMR (600 MHz, DMSO-*d*_*6*_): *δ* 9.56 (s, 1H, NH), 8.67 (s, 1H, CH), 8.49 (s, 1H, CH), 8.27 (s, 1H, Ar-H), 7.95–7.86 (m, 2H, Ar-H), 7.56 (d, *J* = 7.7 Hz, 1H, Ar-H), 7.51–7.28 (m, 6H, Ar-H), 7.24 (s, 1H, Ar-H), 5.67 (s, 2H, CH_2_), 4.33–4.29 (m, 4H, CH_2_CH_2_), 3.81–3.75 (m, 4H, CH_2_CH_2_), 3.39 (s, 3H, CH_3_), 3.36 (s, 3H, CH_3_). ^13^C NMR (150 MHz, DMSO-*d*_*6*_): 156.8, 154.0, 153.4, 148.5, 147.4, 147.1, 140.5, 136.5, 131.3, 129.4, 129.3, 128.6, 128.4, 122.2, 122.1, 120.7, 119.2, 109.4, 108.6, 103.6, 70.6, 70.5, 68.8, 68.5, 58.8, 58.8, 53.5; HR MS (ESI) *m/z*: calcd for C_29_H_30_O_4_N_6_Na (M + Na)^+^ 549.2221, found 549.2231.

##### [6,7-Bis-(2-Methoxy-Ethoxy)-Quinazolin-4-yl]-{3-[1-(2-Iodo-Benzyl)-1H-(1,2,3)Triazol-4-yl]-Phenyl}-Amine (3b)

Purity 98%; m. p. 93–96°C; ^1^H NMR (600 MHz, DMSO-*d*_*6*_): *δ* 9.63 (s, 1H, NH), 8.64 (s, 1H, CH), 8.54 (s, 1H, CH), 8.32 (s, 1H, Ar-H), 8.06–7.90 (m, 3H, Ar-H), 7.63 (d, *J* = 7.7 Hz, 1H, Ar-H), 7.50 (dd, *J* = 16.4, 8.0 Hz, 2H, Ar-H), 7.28 (s, 1H, Ar-H), 7.20 (dd, *J* = 11.8, 7.6 Hz, 2H, Ar-H), 5.75 (s, 2H, CH_2_), 4.37–4.34 (m, 4H, CH_2_CH_2_), 3.85–3.80 (m, 4H, CH_2_CH_2_), 3.43 (s, 3H, CH_3_), 3.41 (s, 3H, CH_3_). ^13^C NMR (150 MHz, DMSO-*d*_*6*_): 156.8, 154.0, 153.3, 148.5, 147.3, 146.9, 140.5, 140.0, 138.3, 131.3, 130.8, 130.1, 129.4, 129.3, 122.5, 122.3, 120.8, 119.3, 109.4, 108.5, 103.7, 99.7, 70.5, 70.5, 68.8, 68.5, 58.8, 58.8, 58.0; HR MS (ESI) *m/z*: calcd for C_29_H_29_O_4_N_6_INa (M + Na)^+^ 675.1187, found 675.1196.

##### [6,7-Bis-(2-Methoxy-Ethoxy)-Quinazolin-4-yl]-{3-[1-(2-Bromo-Benzyl)-1H-(1,2,3)Triazol-4-yl]-Phenyl}-Amine (3c)

Purity 99%; m. p. 94–97°C; ^1^H NMR (600 MHz, DMSO-*d*_*6*_): *δ* 9.60 (s, 1H, NH), 8.63 (s, 1H, CH), 8.50 (s, 1H, CH), 8.27 (s, 1H, Ar-H), 7.98–7.84 (m, 2H, Ar-H), 7.72 (d, *J* = 7.9 Hz, 1H, Ar-H), 7.58 (d, *J* = 7.7 Hz, 1H, Ar-H), 7.46 (dt, *J* = 11.5, 7.7 Hz, 2H, Ar-H), 7.34 (t, *J* = 8.3 Hz, 1H, Ar-H), 7.29–7.16 (m, 2H, Ar-H), 5.76 (s, 2H, CH_2_), 4.33–4.29 (m, 4H, CH_2_CH_2_), 3.81–3.75 (m, 4H, CH_2_CH_2_), 3.38 (s, 3H, CH_3_), 3.36 (s, 3H, CH_3_). ^13^C NMR (150 MHz, DMSO-*d*_*6*_): 156.8, 154.1, 153.3, 148.5, 147.2, 146.9, 140.4, 135.2, 133.4, 131.3, 131.0, 129.9, 129.5, 128.8, 123.3, 122.5, 122.4, 120.9, 119.3, 109.4, 108.5, 103.7, 87.3, 70.6, 70.5, 68.8, 68.5, 58.8, 58.8, 53.6; HR MS (ESI) *m/z*: calcd for C_29_H_29_O_4_N_6_BrNa (M + Na)^+^ 627.1331, found 627.1336.

##### [6,7-Bis-(2-Methoxy-Ethoxy)-Quinazolin-4-yl]-{3-[1-(3,5-Dibromo-Benzyl)-1H-(1,2,3)Triazol-4-yl]-Phenyl}-Amine (3d)

Purity 98%; m. p. 102–105°C; ^1^H NMR (600 MHz, DMSO-*d*_*6*_): *δ* 9.58 (s, 1H, NH), 8.72 (s, 1H, CH), 8.49 (s, 1H, CH), 8.28 (s, 1H, Ar-H), 7.99–7.89 (m, 2H, Ar-H), 7.86 (s, 1H, Ar-H), 7.64 (s, 2H, Ar-H), 7.57 (d, *J* = 7.6 Hz, 1H, Ar-H), 7.47 (t, *J* = 7.9 Hz, 1H, Ar-H), 7.24 (s, 1H, Ar-H), 5.70 (s, 2H, CH_2_), 4.33–4.29 (m, 4H, CH_2_CH_2_), 3.81–3.75 (m, 4H, CH_2_CH_2_), 3.39 (s, 3H, CH_3_), 3.36 (s, 3H, CH_3_); ^13^C NMR (150 MHz, DMSO-*d*_*6*_): 156.8, 154.0, 153.3, 148.5, 147.4, 147.2, 140.8, 140.5, 133.7, 131.2, 130.6, 129.5, 123.2, 122.4, 120.8, 119.3, 109.4, 108.6, 103.7, 70.6, 70.5, 68.8, 68.5, 58.8, 58.8, 52.0; HR MS(ESI) *m/z*: calcd for C_29_H_29_O_4_N_6_Br_2_Na (M + Na)^+^ 683.0612, found 683.0624.

##### [6,7-Bis-(2-Methoxy-Ethoxy)-Quinazolin-4-yl]-{3-[1-(3-Methoxy-Phenyl)-1H-(1,2,3)Triazol-4-yl]-Phenyl}-Amine (3e)

Purity 99%; m. p. 85–88°C; ^1^H NMR (600 MHz, DMSO-*d*_*6*_): *δ* 9.61 (s, 1H, NH), 8.66 (s, 1H, CH), 8.27 (s, 1H, CH), 8.01 (s, 1H, Ar-H), 7.91 (d, *J* = 7.9 Hz, 1H, Ar-H), 7.57 (d, *J* = 7.6 Hz, 1H, Ar-H), 7.46 (t, *J* = 7.9 Hz, 1H, Ar-H), 7.32 (t, *J* = 7.9 Hz, 2H, Ar-H), 6.97 (s, 1H, Ar-H), 6.93 (d, *J* = 7.9 Hz, 2H, Ar-H), 5.63 (s, 2H, CH_2_), 4.33–4.31 (m, 4H, CH_2_CH_2_), 3.80–3.76 (m, 4H, CH_2_CH_2_), 3.76 (s, 3H, OCH_3_), 3.39 (s, 3H, CH_3_), 3.37 (s, 3H, CH_3_).^13^C NMR (150 MHz, DMSO-*d*_*6*_): 159.9, 156.7, 154.0, 148.6, 147.0, 140.4, 137.9, 131.4, 130.4, 129.5, 122.3, 122.1, 120.8, 120.5, 119.3, 114.2, 113.9, 108.9, 103.8, 87.7, 70.6, 70.5, 68.8, 68.5, 58.8, 58.8, 55.6, 53.4, 22.5; HR MS(ESI) *m/z*: calcd for C_30_H_33_O_5_N_6_ (M + H)^+^ 557.2512, found 557.2508.

##### [6,7-Bis-(2-Methoxy-Ethoxy)-Quinazolin-4-yl]-{3-[1-(2-Fluoro-Phenyl)-1H-(1,2,3)Triazol-4-yl]-Phenyl}-Amine (3f)

Purity 98%; m. p. 83–86°C; ^1^H NMR (600 MHz, DMSO-*d*_*6*_): *δ* 9.62 (s, 1H, NH), 9.11 (s, 1H, CH), 8.51 (s, 1H, CH), 8.39 (s, 1H, Ar-H), 8.04–7.86 (m, 3H, Ar-H), 7.73–7.59 (m, 3H, Ar-H), 7.56–7.46 (m, 2H, Ar-H), 7.25 (s, 1H, Ar-H), 4.34–4.30 (m, 4H, CH_2_CH_2_), 3.82–3.76 (m, 4H, CH_2_CH_2_), 3.39 (s, 3H, CH_3_), 3.37 (s, 3H, CH_3_). ^13^C NMR (150 MHz, DMSO-*d*_*6*_): 156.8, 155.2, 154.0, 153.4, 148.5, 147.4, 140.6, 131.8, 130.7, 129.6, 126.5, 126.0, 123.4, 122.7, 121.0, 119.4, 117.7, 117.6, 109.4, 108.6, 103.7, 70.6, 70.5, 68.8, 68.5, 58.8, 58.8; HR MS (ESI) *m/z*: calcd for C_28_H_27_O_4_N_6_FNa (M + Na)^+^ 553.1970, found 553.1979.

##### [6,7-Bis-(2-Methoxy-Ethoxy)-Quinazolin-4-yl]-{3-[1-(4-Fluoro-Phenyl)-1H-(1,2,3)Triazol-4-yl]-Phenyl}-Amine (3g)

Purity 97%; m. p. 88–91°C; ^1^H NMR (600 MHz, DMSO-*d*_*6*_): *δ* 9.73 (s, 1H, NH), 9.32 (s, 1H, CH), 8.57 (s, 1H, CH), 8.38 (s, 1H, Ar-H), 8.14–7.86 (m, 4H, Ar-H), 7.67 (d, *J* = 7.6 Hz, 1H, Ar-H), 7.52 (dt, *J* = 12.5, 8.3 Hz, 3H, Ar-H), 7.26 (s, 1H, Ar-H), 4.34–4.30 (m, 4H, CH_2_CH_2_), 3.82–3.76 (m, 4H, CH_2_CH_2_), 3.39 (s, 3H, CH_3_), 3.37 (s, 3H, CH_3_); ^13^C NMR (150 MHz, DMSO-*d*_*6*_): 162.9, 161.3, 156.9, 156.9, 154.2, 153.1, 148.6, 147.8, 140.4, 133.7, 130.9, 129.6, 122.8, 122.8, 120.4, 119.6, 117.3, 117.2, 108.3, 103.7, 70.5, 70.5, 68.8, 68.5, 58.8, 58.8; HR MS(ESI) *m/z*: calcd for C_28_H_27_O_4_N_6_FNa (M + Na)^+^ 553.1970, found 553.1979.

##### [6,7-Bis-(2-Methoxy-Ethoxy)-Quinazolin-4-yl]-{3-[1-(2-Chloro-Phenyl)-1H-(1,2,3)Triazol-4-yl]-Phenyl}-Amine (3h)

Purity 99%; m. p. 131–134°C; ^1^H NMR (600 MHz, DMSO-*d*_*6*_): *δ* 9.62 (s, 1H, NH), 9.08 (s, 1H, CH), 8.51 (s, 1H, CH), 8.40 (s, 1H, Ar-H), 7.94 (d, *J* = 10.6 Hz, 2H, Ar-H), 7.85–7.79 (m, 2H, Ar-H), 7.70–7.61 (m, 3H, Ar-H), 7.52 (t, *J* = 7.9 Hz, 1H, Ar-H), 7.25 (s, 1H, Ar-H), 4.34–4.30 (m, 4H, CH_2_CH_2_), 3.81–3.76 (m, 4H, CH_2_CH_2_), 3.39 (s, 3H, CH_3_), 3.37 (s, 3H, CH_3_); ^13^C NMR (150 MHz, DMSO-*d*_*6*_): 156.8, 154.0, 153.4, 148.5, 147.4, 146.9, 140.6, 135.0, 131.0, 130.8, 129.6, 129.1, 129.0, 128.9, 124.1, 122.6, 121.0, 119.4, 109.4, 108.6, 103.7, 70.6, 70.5, 68.8, 68.5, 58.8, 58.8; HR MS(ESI) *m/z*: calcd for C_28_H_27_O_4_N_6_ClNa (M + Na)^+^ 569.1675, found 569.1678.

##### [6,7-Bis-(2-Methoxy-Ethoxy)-Quinazolin-4-yl]-{3-[1-(2-Bromo-Phenyl)-1H-(1,2,3)Triazol-4-yl]-Phenyl}-Amine (3i)

Purity 97%; m. p. 93–97°C; ^1^H NMR (600 MHz, DMSO-*d*_*6*_): *δ* 9.63 (s, 1H, NH), 9.05 (s, 1H, CH), 8.51 (s, 1H, CH), 8.40 (s, 1H, Ar-H), 8.00–7.89 (m, 3H, Ar-H), 7.77 (dd, *J* = 7.8, 1.5 Hz, 1H, Ar-H), 7.67 (t, *J* = 7.7 Hz, 2H, Ar-H), 7.60 (t, *J* = 8.6 Hz, 1H, Ar-H), 7.52 (t, *J* = 7.9 Hz, 1H, Ar-H), 7.25 (s, 1H, Ar-H), 4.34–4.30 (m, 4H, CH_2_CH_2_), 3.81–3.76 (m, 4H, CH_2_CH_2_), 3.39 (s, 3H, CH_3_), 3.37 (s, 3H, CH_3_); ^13^C NMR (150 MHz, DMSO-*d*_*6*_): 156.8, 154.1, 153.3, 148.5, 147.4, 146.9, 140.6, 136.7, 134.1, 132.5, 130.9, 129.6, 129.4, 129.2, 124.2, 122.6, 121.0, 119.4, 109.4, 108.6, 103.7, 100.0, 70.6, 70.5, 68.8, 68.5, 58.8, 58.8; HR MS(ESI) *m/z*: calcd for C_28_H_27_O_4_N_6_BrNa (M + Na)^+^ 613.1169, found 613.1180.

##### [6,7-Bis-(2-Methoxy-Ethoxy)-Quinazolin-4-yl]-{3-[1-(4-Bromo-Phenyl)-1H-(1,2,3)Triazol-4-yl]-Phenyl}-Amine (3j)

Purity 98%; m. p. 105–108°C; ^1^H NMR (600 MHz, DMSO-*d*_*6*_): *δ* 9.63 (s, 1H, NH), 9.37 (s, 1H, CH), 8.51 (s, 1H, CH), 8.38 (s, 1H, Ar-H), 7.96 (dd, *J* = 16.5, 7.6 Hz, 4H, Ar-H), 7.86 (d, *J* = 8.8 Hz, 2H, Ar-H), 7.66 (d, *J* = 7.6 Hz, 1H, Ar-H), 7.53 (t, *J* = 7.9 Hz, 1H, Ar-H), 7.25 (s, 1H, Ar-H), 4.34–4.30 (m, 4H, CH_2_CH_2_), 3.82–3.76 (m, 4H, CH_2_CH_2_), 3.39 (s, 3H, CH_3_), 3.37 (s, 3H, CH_3_); ^13^C NMR (150 MHz, DMSO-*d*_*6*_): 156.8, 154.0, 153.4, 148.5, 147.9, 147.4, 140.6, 136.3, 133.3, 130.8, 129.6, 122.7, 122.3, 121.8, 121.0, 120.1, 119.4, 109.4, 108.6, 103.6, 70.6, 70.5, 68.8, 68.5, 58.8, 58.8; HR MS(ESI) *m/z*: calcd for C_28_H_27_O_4_N_6_BrNa (M + Na)^+^ 613.1169, found 613.1177.

##### [6,7-Bis-(2-Methoxy-Ethoxy)-Quinazolin-4-yl]-{3-[1-(2-Methoxy-Phenyl)-1H-[1,2,3]Triazol-4-yl]-Phenyl}-Amine (3k)

Purity 98%; m. p. 87–90°C; ^1^H NMR (600 MHz, DMSO-*d*_*6*_): *δ* 9.62 (s, 1H, NH), 8.94 (s, 1H, CH), 8.50 (s, 1H, CH), 8.36 (s, 1H, Ar-H), 7.98–7.91 (m, 2H, Ar-H), 7.69 (dd, *J* = 21.0, 7.7 Hz, 2H, Ar-H), 7.58 (t, *J* = 7.9 Hz, 1H, Ar-H), 7.50 (t, *J* = 7.9 Hz, 1H, Ar-H), 7.36 (d, *J* = 8.2 Hz, 1H, Ar-H), 7.25 (s, 1H, Ar-H), 7.19 (t, *J* = 7.6 Hz, 1H, Ar-H), 4.34–4.30 (m, 4H, CH_2_CH_2_), 3.90 (s, 3H, OCH_3_), 3.82–3.76 (m, 4H, CH_2_CH_2_), 3.39 (s, 3H, CH_3_), 3.37 (s, 3H, CH_3_); ^13^C NMR (150 MHz, DMSO-*d*_*6*_): 156.8, 154.0, 153.4, 152.3, 148.5, 147.4, 146.6, 140.5, 131.3, 131.2, 129.5, 126.4, 126.2, 123.9, 122.5, 121.3, 121.0, 119.4, 113.4, 109.4, 108.6, 103.7, 68.8, 68.5, 58.8, 58.5, 56.6; HR MS(ESI) *m/z*: calcd for C_29_H_30_O_5_N_6_Na (M + Na)^+^ 565.2170, and found 565.2172.

##### [6,7-Bis-(2-Methoxy-Ethoxy)-Quinazolin-4-yl]-[3-(1-p-Tolyl-1H-(1,2,3)Triazol-4-yl)-Phenyl]-Amine (3l)

Purity 97%; m. p. 95–98°C; ^1^H NMR (600 MHz, DMSO-*d*_*6*_): *δ* 9.63 (s, 1H, NH), 9.28 (s, 1H, CH), 8.51 (s, 1H, CH), 8.37 (s, 1H, Ar-H), 7.95 (d, *J* = 9.9 Hz, 2H, Ar-H), 7.87 (d, *J* = 8.3 Hz, 2H, Ar-H), 7.67 (d, *J* = 7.6 Hz, 1H, Ar-H), 7.52 (t, *J* = 7.9 Hz, 1H, Ar-H), 7.45 (d, *J* = 8.3 Hz, 2H, Ar-H), 7.25 (s, 1H, Ar-H), 4.34–4.30 (m, 4H, CH_2_CH_2_), 3.82–3.76 (m, 4H, CH_2_CH_2_), 3.39 (s, 3H, CH_3_), 3.37 (s, 3H, CH_3_), 2.51 (s, 3H, CH_3_); ^13^C NMR (150 MHz, DMSO-*d*_*6*_): 156.8, 154.0, 153.4, 148.5, 147.7, 147.4, 140.5, 138.8, 134.8, 131.0, 130.7, 129.5, 122.6, 121.0, 120.3, 120.0, 119.4, 109.4, 108.6, 103.6, 70.6, 70.5, 68.8, 68.5, 58.8, 58.8, 21.0; HR MS(ESI) *m/z*: calcd for C_29_H_31_O_4_N_6_ (M + H)^+^ 527.2401, found 527.2410.

##### [6,7-Bis-(2-Methoxy-Ethoxy)-Quinazolin-4-yl]-{3-[1-(3-Nitro-Phenyl)-1H-(1,2,3)Triazol-4-yl]-Phenyl}-Amine (3m)

Purity 99%; m. p. 98–101°C; ^1^H NMR (600 MHz, DMSO-*d*_*6*_): *δ* 9.64 (s, 1H, NH), 9.60 (s, 1H, CH), 8.83 (t, *J* = 2.1 Hz, 1H, CH), 8.50 (d, *J* = 12.4 Hz, 2H, Ar-H), 8.41 (s, 1H, Ar-H), 8.37 (d, *J* = 7.5 Hz, 1H, Ar-H), 7.99–7.94 (m, 3H, Ar-H), 7.69 (d, *J* = 7.8 Hz, 1H, Ar-H), 7.55 (t, *J* = 7.9 Hz, 1H, Ar-H), 7.25 (s, 1H, Ar-H), 4.34–4.30 (m, 4H, CH_2_CH_2_), 3.82–3.76 (m, 4H, CH_2_CH_2_), 3.39 (s, 3H, CH_3_), 3.37 (s, 3H, CH_3_); ^13^C NMR (150 MHz, DMSO-*d*_*6*_): 156.8, 154.0, 153.4, 149.0, 148.5, 147.4, 140.6, 137.7, 132.0, 130.6, 129.6, 126.4, 123.6, 122.9, 121.0, 120.6, 119.5, 115.0, 109.4, 108.6, 103.6, 70.6, 70.5, 68.8, 68.5, 58.8, 58.8; HR MS(ESI) *m/z*: calcd for C_28_H_27_O_6_N_7_Na (M + Na)^+^ 580.1915, found 580.1923.

##### [6,7-Bis-(2-Methoxy-Ethoxy)-Quinazolin-4-yl]-{3-[1-(3-Ethoxy-Phenyl)-1H-(1,2,3)Triazol-4-yl]-Phenyl}-Amine (3n)

Purity 98%; m. p. 110–114°C; ^1^H NMR (600 MHz, DMSO-*d*_*6*_): *δ* 9.63 (s, 1H, NH), 9.36 (s, 1H, CH), 8.51 (s, 1H, CH), 8.37 (s, 1H, Ar-H), 7.95 (d, *J* = 10.1 Hz, 2H, Ar-H), 7.67 (d, J = 7.7 Hz, 1H, Ar-H), 7.55 (d, *J* = 36.4 Hz, 4H, Ar-H), 7.25 (s, 1H, Ar-H), 7.08 (d, *J* = 10.2 Hz, 1H, Ar-H), 4.34–4.30 (m, 4H, CH_2_CH_2_), 4.17 (q, *J* = 7.0 Hz, 2H, CH_2_), 3.82–3.76 (m, 4H, CH_2_CH_2_), 3.39 (s, 3H, CH_3_), 3.37 (s, 3H, CH_3_), 1.39 (t, *J* = 7.0 Hz, 3H, CH_3_); ^13^C NMR (150 MHz, DMSO-*d*_*6*_): 159.9, 156.8, 154.0, 153.4, 148.5, 147.8, 147.4, 140.6, 138.1, 131.3, 130.9, 129.5, 122.7, 121.0, 120.1, 119.4, 115.2, 112.2, 109.4, 108.6, 106.4, 103.6, 70.6, 70.5, 68.8, 68.5, 64.1, 58.8, 58.8, 15.0; HR MS(ESI) *m/z*: calcd for C_30_H_32_O_5_N_6_Na (M + Na)^+^ 579.2326, found 579.2332.

##### [6,7-Bis-(2-Methoxy-Ethoxy)-Quinazolin-4-yl]-[3-(1-Phenyl-1H-(1,2,3)Triazol-4-yl)-Phenyl]-Amine (3o)

Purity 96%; m. p. 137–140°C; ^1^H NMR (600 MHz, DMSO-*d*_*6*_): *δ* 9.63 (s, 1H, NH), 9.34 (s, 1H, CH), 8.50 (s, 1H, CH), 8.38 (s, 1H, Ar-H), 7.99 (d, *J* = 7.6 Hz, 2H, Ar-H), 7.94 (d, *J* = 9.8 Hz, 2H, Ar-H), 7.66 (dd, *J* = 16.3, 8.6 Hz, 3H, Ar-H), 7.53 (d, *J* = 18.2 Hz, 2H, Ar-H), 7.24 (s, 1H, Ar-H), 4.34–4.29 (m, 4H, CH_2_CH_2_), 3.81–3.76 (m, 4H, CH_2_CH_2_), 3.39 (s, 3H, CH_3_), 3.36 (s, 3H, CH_3_); ^13^C NMR (150 MHz, DMSO-*d*_*6*_): 156.8, 154.0, 153.4, 148.5, 147.8, 147.4, 140.6, 137.1, 130.9, 130.4, 129.5, 129.2, 122.7, 121.0, 120.4, 120.1, 119.4, 109.4, 108.6, 103.6, 70.6, 70.5, 68.8, 68.5, 58.8, 58.8; HR MS(ESI) *m/z*: calcd for C_28_H_28_O_4_N_6_Na (M + Na)^+^ 535.2064, found 535.2069.

##### [6,7-Bis-(2-Methoxy-Ethoxy)-Quinazolin-4-yl]-{3-[1-(2-Trifluoromethyl-Phenyl)-1H-(1,2,3)Triazol-4-yl]-Phenyl}-Amine (3p)

Purity 97%; m. p. 113–116°C; ^1^H NMR (400 MHz, DMSO-*d*_*6*_): *δ* 9.62 (s, 1H, NH), 9.05 (s, 1H, CH), 8.51 (s, 1H, CH), 8.39 (s, 1H, Ar-H), 8.08 (d, *J* = 7.1 Hz, 1H, Ar-H), 7.93 (d, *J* = 61.3 Hz, 5H, Ar-H), 7.65 (d, J = 7.8 Hz, 1H, Ar-H), 7.52 (t, *J* = 7.9 Hz, 1H, Ar-H), 7.24 (s, 1H, Ar-H), 4.34–4.29 (m, 4H, CH_2_CH_2_), 3.81–3.75 (m, 4H, CH_2_CH_2_), 3.39 (s, 3H, CH_3_), 3.37 (s, 3H, CH_3_); ^13^C NMR (100 Hz, DMSO-*d*_*6*_): 156.8, 154.0, 153.3, 148.5, 147.3, 146.9, 140.6, 134.5, 131.7, 130.7, 129.8, 129.6, 127.9, 125.5, 125.2, 124.7, 122.7, 121.9, 121.0, 119.4, 109.3, 108.5, 103.5, 70.5, 70.5, 68.7, 68.4, 58.8, 58.8; HR MS (ESI) *m/z*: calcd for C_29_H_27_O_4_N_6_F_3_Na (M + Na)^+^ 603.1938, found 603.1945.

##### [6,7-Bis-(2-Methoxy-Ethoxy)-Quinazolin-4-yl]-{3-[1-(2,4-Dimethoxy-Phenyl)-1H-(1,2,3)Triazol-4-yl]-Phenyl}-Amine (3q)

Purity 98%; m. p. 85–88°C; ^1^H NMR (400 MHz, DMSO-*d*_*6*_): *δ* 9.66 (s, 1H, NH), 8.88 (s, 1H, CH), 8.55 (s, 1H, CH), 8.39 (s, 1H, Ar-H), 8.02–7.92 (m, 2H, Ar-H), 7.70 (d, J = 7.8 Hz, 1H, Ar-H), 7.63 (d, *J* = 8.7 Hz, 1H, Ar-H), 7.54 (t, *J* = 7.9 Hz, 1H, Ar-H), 7.29 (s, 1H, Ar-H), 6.92 (d, *J* = 2.5 Hz, 1H, Ar-H), 6.78 (dd, *J* = 8.8, 2.6 Hz, 1H, Ar-H), 4.39–4.34 (m, 4H, CH_2_CH_2_), 3.93 (s, 3H, OCH_3_), 3.92 (s, 3H, OCH_3_), 3.87–3.80 (m, 4H, CH_2_CH_2_), 3.44 (s, 3H, CH_3_), 3.42 (s, 3H, CH_3_); ^13^C NMR (100 MHz, DMSO-*d*_*6*_): 161.7, 156.8, 154.0, 153.7, 153.3, 148.5, 146.4, 140.5, 131.2, 129.4, 127.4, 124.0, 122.4, 120.9, 119.6, 119.3, 108.6, 105.7, 103.6, 99.9, 70.5, 70.5, 68.7, 68.4, 58.8, 58.8, 56.6, 56.1; HR MS (ESI) *m/z*: calcd for C_30_H_32_O_6_N_6_Na (M + Na)^+^ 595.2276, found 595.2285.

##### 2-(4-{3-[6,7-Bis-(2-Methoxy-Ethoxy)-Quinazolin-4-Ylamino]-Phenyl}-(1,2,3)Triazol-1-yl)-5-Methyl-Phenol (3r)

Purity 98%; m. p. 100–103°C; ^1^H NMR (400 MHz, DMSO-*d*_*6*_): *δ* 9.66 (s, 1H, NH), 8.98 (s, 1H, CH), 8.55 (s, 1H, CH), 8.40 (s, 1H, OH), 7.99 (d, *J* = 12.3 Hz, 2H, Ar-H), 7.73 (dd, *J* = 14.5, 7.8 Hz, 2H, Ar-H), 7.63 (t, *J* = 7.9 Hz, 1H, Ar-H), 7.55 (t, *J* = 7.9 Hz, 1H, Ar-H), 7.41 (d, *J* = 7.7 Hz, 1H, Ar-H), 7.29 (s, 1H, Ar-H), 7.24 (t, J = 7.6 Hz, 1H, Ar-H), 4.39–4.34 (m, 4H, CH_2_CH_2_), 3.95 (s, 3H, CH_3_), 3.87–3.80 (m, 4H, CH_2_CH_2_), 3.44 (s, 3H, CH_3_), 3.42 (s, 3H, CH_3_); ^13^C NMR (100 MHz, DMSO-*d*_*6*_): 156.8, 154.0, 153.3, 152.2, 148.5, 147.3, 146.6, 140.5, 131.4, 131.1, 129.5, 126.4, 126.1, 123.9, 122.5, 121.3, 121.0, 119.3, 113.4, 109.4, 108.5, 103.6, 70.5, 70.5, 68.7, 68.4, 58.8, 58.8, 56.6; HR MS (ESI) *m/z*: calcd for C_29_H_30_O_5_N_6_Na (M + Na)^+^ 565.2170, found 565.2175.

##### [6,7-Bis-(2-Methoxy-Ethoxy)-Quinazolin-4-yl]-{3-[1-Phenethyl-1H-(1,2,3)Triazol-4-yl]-Phenyl}-Amine (3s)

Purity 97%; m. p. 109–112°C; ^1^H NMR (600 MHz, DMSO-*d*_*6*_): *δ* 9.56 (s, 1H, NH), 8.53 (s, 1H, CH), 8.49 (s, 1H, CH), 8.24 (s, 1H, Ar-H), 7.93 (s, 1H, Ar-H), 7.89 (d, J = 8.9 Hz, 1H, Ar-H), 7.51 (d, *J* = 7.7 Hz, 1H, Ar-H), 7.45 (t, *J* = 7.8 Hz, 1H, Ar-H), 7.29 (t, J = 7.4 Hz, 2H, Ar-H), 7.22 (dd, J = 13.1, 6.9 Hz, 4H, Ar-H), 4.68 (t, J = 7.3 Hz, 2H, CH_2_), 4.33–4.29 (m, 4H,CH_2_CH_2_), 3.81–3.75 (m, 4H,CH_2_CH_2_), 3.38 (s, 3H, CH_3_), 3.36 (s, 3H, CH_3_), 3.24 (t, *J* = 7.3 Hz, 2H, CH_2_); ^13^C NMR (150 MHz, DMSO-*d*_*6*_): 156.8, 154.0, 153.3, 148.5, 147.4, 146.5, 140.5, 138.1, 131.5, 129.4, 129.1, 127.0, 122.1, 121.8, 120.6, 119.1, 109.4, 108.6, 103.6, 70.6, 70.5, 68.8, 68.5, 58.8, 58.8, 51.1, 36.0; HR MS(ESI) *m/z*: calcd for C_30_H_32_O_4_N_6_Na (M + Na)^+^ 563.2377, found 563.2381.

### Biological Assay

#### Cell Culture and Treatment

The human cancer cells H1650/H1650TR, HCC827/HCC827GR, KYSE70/KYSE70TR, KYSE410/KYSE410TR, and KYSE450/KYSE450TR were cultured in the RPMI-1640 complete growth medium containing 100 U/mL of penicillin–streptomycin and 10% FBS. The cells were incubated at 37°C with 5% of CO_2_. The compounds were dissolved in DMSO to make a 50 mM stock solution and were diluted to the concentration of working solutions with the complete growth medium before administration.

#### MTT Assay for Cell Proliferation and Cytotoxicity

Cells were seeded in 96-well plates with densities of 2,200–2,500 cells/well in 100 μL. One day after seeding, the concentration of the test compounds between 0 and 50 μM, 0.1% DMSO was added to cells as control. Approximately 2200-2500 transfected cells in 100 μL were incubated in quintuplicate in 96-well plates. After 48 h, MTT was added and incubated in the plate for 1–4 h in the incubator. The absorbance at 490 nm was measured using a microplate reader (Thermo).

#### Culture Assay for Tumor Colony-forming Cells

Cells were seeded in 6-well plates with a density of 200 cells/well and cultured overnight for attachment. The cells were exposed to **3d** or erlotinib of various concentrations (0, 2.5, 5, and 10 µM) separately for 10 days. Medium with or without compounds was changed every 48 h. When colony formation was visible, the medium was discharged. Then, the colonies were washed with cold PBS, fixed with 4% paraformaldehyde (PFA) for at least 30 min, and then stained with 0.2% crystal violet solution in 100% ethanol for 20 min.

#### Flow Cytometry Detection for Cell Apoptosis

Cell apoptosis analysis was carried out by flow cytometry using the Annexin V/PI apoptosis methods. Briefly, KYSE450/KYSE450TR (2 × 10^4^- 3×10^4^/well) cells were incubated in 6-well plates for 48 h and then treated with 0.1% DMSO (as control), either compound **3d** or erlotinib at various concentrations for 48 h, respectively. The cells were harvested and incubated with 250 μL of 1 × Annexin V binding buffer containing 5 μL of PI and 5 μL of FITC Annexin V (final concentration 1.8 μg/ml, Biolegend cat: 640945) for 15 min at room temperature in the dark. Then 200 µL of 1 × binding buffer was added for flow cytometry analysis (BD FACSCalibur™ Flow Cytometer).

#### Western Blot Analysis

KYSE450 cells (3 × 10^5^/well) were incubated overnight in 10 square petri dishes and then treated with compound **3d** at 4 μM for 0, 6, 12, and 24 h. Cells treated with 0.1% DMSO were used as control. Then, the cells were harvested, and total proteins were extracted. Total proteins were separated by 12% SDS polyacrylamide gel electrophoresis and transferred onto PVDF membranes. The membrane was blocked for 1 h, then incubated overnight with a 1:1000 dilution of anti-caspase-3, anti-cytochrome-c, and anti-PARP, or 1 : 3,000 dilution of anti-β-action primary antibody at 4°C. Finally, 1 : 3,000 anti-rabbit secondary antibodies were incubated for 2 h at room temperature. Protein bands were developed by chemiluminescence.

#### Cell Cycle Analysis

KYSE450 and KYSE450TR cells were plated in 6-well plates with a density of 1 × 10^5^ cells/well and cultured overnight to attach. The cells were treated with **3d** or erlotinib at different concentrations (0, 3.5, 7, 14, and 28 μM) for 24 h. After trypsinization treatment, the cells were collected by centrifugation. The cell pellet was re-suspended in 70% ethanol at −20°C for at least 3 h. The cells were washed with PBS and was re-suspended in 250 μL of 0.6% tricine with renease A for 1 h, then stained in PI (final concentration 1.8 μg/ml, Biolegend cat: 640945) for 15 min in the dark. Cells were re-transferred to the BD FACSCalibur™ Flow Cytometer. All analyses were performed with FlowJo software v105.3.6.

#### EGFR Protein Affinity Was Determined by SPR

Surface plasmon resonance experiments were carried out to evaluate the interaction between **3d** with the EGFR wild-type (0.468 μg/μL, Active MOTIF, cat: 31165) and the EGFR mutant type (672-1210, L858R, Active MOTIF, cat: 81200). The interaction of erlotinib with wild-type EGFR was also studied. Biacore T-200 (GE healthcare, Waukesha, WI, United States) equipment was used for the study. First, the EGFR wild-type (0.468 μg/μL, Active MOTIF, cat: 31165) and the EGFR mutant type (672-1210, L858R, Active MOTIF, cat: 81200) were covalently immobilized at densities of 2000 response units onto a CM5 sensor chip. Then, **3d** dissolved in DMSO was injected at concentrations between 0.064 and 5,000 nM at 25°C. The final doses of DMSO did not exceed 1% (v/v). During the interaction, the changes in the refractive index were measured in real time to allow the plotting of the results of interaction as response units versus time. The interaction results were analyzed with BIA evaluation 3.0 software.

## Data Availability

The original contributions presented in the study are included in the article/Supplementary Material; further inquiries can be directed to the corresponding authors.
