# Supplementary material for: Design, Synthesis, and Antitumor Activity of Erlotinib Derivatives
Source: Front Pharmacol. 2022 Apr 20;13:849364. doi: 10.3389/fphar.2022.849364 (PMC9065260; doi:10.3389/fphar.2022.849364)

Supplementary Information

Design, Synthesis and Antitumor Activity of Erlotinib Derivatives

Longfei Mao[a ∇], Zhen-zhen Wang[a∇], Qiong Wu[a], Xiao-jie Chen[b], Jian-xue Yang[b,c,*], Xin Wang[a,*] and Yue-Ming Li[a,*]

[a] State Key Laboratory of Medicinal Chemical Biology, College of Pharmacy and Tianjin Key Laboratory of Molecular Drug Research, Nankai University, Haihe Education Park, 38 Tongyan Road, Tianjin 300350, China

[b] Medical College, School of Nursing, Henan University of Science and Technology, 263 Kaiyuan Road, Luoyang 471003, China

[c] Department of Neurology, The First Affiliated Hospital of Henan University of Science and Technology, Luoyang, China

[∇] These authors contributed equally to this work.

* Corresponding authors:

Jianxue Yang, The First Affiliated Hospital of Henan University of Science and Technology, 263 Kaiyuan Road, Luoyang 471003, China. E-mail: Docyjx1969@126.com

Xin Wang, Nankai University, Haihe Education Park, 38 Tongyan Road, Tianjin 300350, China. E-mail: wangxinnk@nankai.edu.cn

Yue-Ming Li, Nankai University, Haihe Education Park, 38 Tongyan Road, Tianjin 300350, China. E-mail: ymli@nankai.edu.cn

Table of Contents

Figure S1-1. ^{1}H NMR spectrum (600MHz, DMSO-d_6) of compound 3a..4
Figure S1-2. ^{13}C NMR spectrum (150MHz, DMSO-d_6) of compound 3a..5
Figure S1-3. HR MS of compound 3a 6
Figure S2-1. ^{1}H NMR spectrum (600MHz, DMSO-d_6) of compound 3b..7
Figure S2-2. ^{13}C NMR spectrum (150MHz, DMSO-d_6) of compound 3b..8
Figure S2-3. HR MS of compound 3b 9
Figure S3-1. ^{1}H NMR spectrum (600MHz, DMSO-d_6) of compound 3c..10
Figure S3-2. ^{13}C NMR spectrum (150MHz, DMSO-d_6) of compound 3c..11
Figure S3-3. HR MS of compound 3c 12
Figure S4-1. ^{1}H NMR spectrum (600MHz, DMSO-d_6) of compound 3d..13
Figure S4-2. ^{13}C NMR spectrum (150MHz, DMSO-d_6) of compound 3d..14
Figure S4-3. HR MS of compound 3d 15
Figure S5-1. ^{1}H NMR spectrum (600MHz, DMSO-d_6) of compound 3e..16
Figure S5-2. ^{13}C NMR spectrum (150MHz, DMSO-d_6) of compound 3e..17
Figure S5-3. HR MS of compound 3e 18
Figure S6-1. ^{1}H NMR spectrum (600MHz, DMSO-d_6) of compound 3f 19
Figure S6-2. ^{13}C NMR spectrum (150MHz, DMSO-d_6) of compound 3f 20
Figure S6-3. HR MS of compound 3f...21
Figure S7-1. ^{1}H NMR spectrum (600MHz, DMSO-d_6) of compound 3g..22
Figure S7-2. ^{13}C NMR spectrum (150MHz, DMSO-d_6) of compound 3g..23
Figure S7-3. HR MS of compound 3g 24
Figure S8-1. ^{1}H NMR spectrum (600MHz, DMSO-d_6) of compound 3h..25
Figure S8-2. ^{13}C NMR spectrum (150MHz, DMSO-d_6) of compound 3h..26
Figure S8-3. HR MS of compound 3h 27
Figure S9-1. ^{1}H NMR spectrum (600MHz, DMSO-d_6) of compound 3i...28
Figure S9-2. ^{13}C NMR spectrum (150MHz, DMSO-d_6) of compound 3i...29
Figure S9-3. HR MS of compound 3i 30
Figure S10-1. ^{1}H NMR spectrum (600MHz, DMSO-d_6) of compound 3j.......................................31
Figure S10-2. ^{13}C NMR spectrum (150MHz, DMSO-d_6) of compound 3j.......................................32
Figure S10-3. HR MS of compound 3j 33
Figure S11-1. ^{1}H NMR spectrum (600MHz, DMSO-d_6) of compound 3k.......................................34
Figure S11-2. ^{13}C NMR spectrum (150MHz, DMSO-d_6) of compound 3k.......................................35
Figure S11-3. HR MS of compound 3k 36
Figure S12-1. ^{1}H NMR spectrum (600MHz, DMSO-d_6) of compound 3l.......................................37
Figure S12-2. ^{12}C NMR spectrum (150MHz, DMSO-d_6) of compound 3l.......................................38
Figure S12-3. HR MS of compound 3l 39
Figure S13-1. ^{1}H NMR spectrum (600MHz, DMSO-d_6) of compound 3m.......................................40
Figure S13-2. ^{13}C NMR spectrum (150MHz, DMSO-d_6) of compound 3m.......................................41

Figure S13-3. HR MS of compound 3m 42
Figure S14-1. ^{1}H NMR spectrum (600MHz, DMSO-d_6) of compound 3n 43
Figure S14-2. ^{13}C NMR spectrum (150MHz, DMSO-d_6) of compound 3n 44
Figure S14-3. HR MS of compound 3n 45
Figure S15-1. ^{1}H NMR spectrum (600MHz, DMSO-d_6) of compound 3o 46
Figure S15-2. ^{13}C NMR spectrum (150MHz, DMSO-d_6) of compound 3o 47
Figure S15-3. HR MS of compound 3o 48
Figure S16-1. ^{1}H NMR spectrum (400MHz, DMSO-d_6) of compound 3p 49
Figure S16-2. ^{13}C NMR spectrum (100MHz, DMSO-d_6) of compound 3p 50
Figure S16-3. HR MS of compound 3p 51
Figure S17-1. ^{1}H NMR spectrum (400MHz, DMSO-d_6) of compound 3q 52
Figure S17-2. ^{13}C NMR spectrum (100MHz, DMSO-d_6) of compound 3q 53
Figure S17-3. HR MS of compound 3q 54
Figure S18-1. ^{1}H NMR spectrum (400MHz, DMSO-d_6) of compound 3r 55
Figure S18-2. ^{13}C NMR spectrum (100MHz, DMSO-d_6) of compound 3r 56
Figure S18-3. HR MS of compound 3r 57
Figure S19-1. ^{1}H NMR spectrum (600MHz, DMSO-d_6) of compound 3s 58
Figure S19-2. ^{13}C NMR spectrum (150MHz, DMSO-d_6) of compound 3s 59
Figure S19-3. HR MS of compound 3s 60

Figure S1-1. ^{1}H NMR spectrum (600MHz, DMSO-d_6) of compound 3a

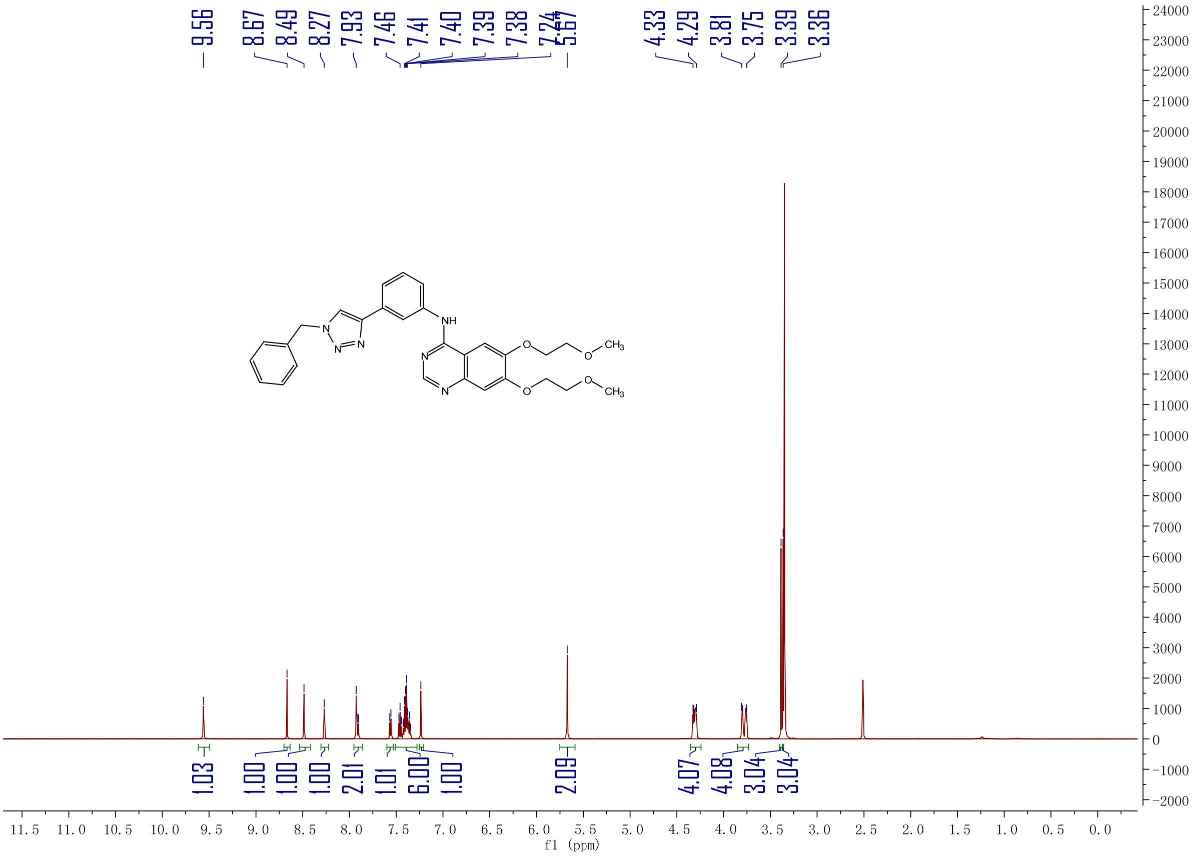

Figure S1-2. ^{13}C NMR spectrum (150MHz, DMSO-d$_6$) of compound 3a

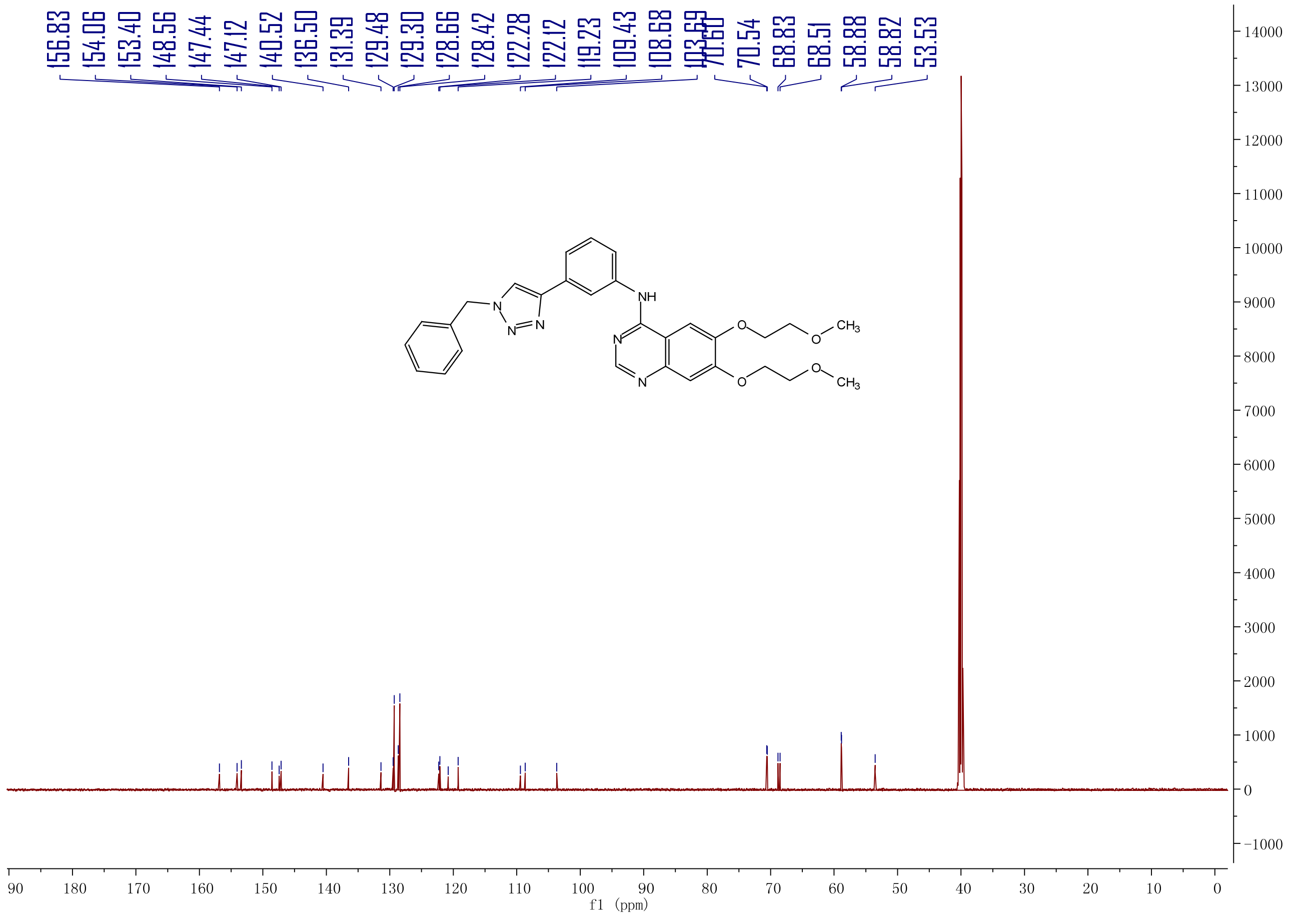

Figure S1-3. HR MS of compound 3a

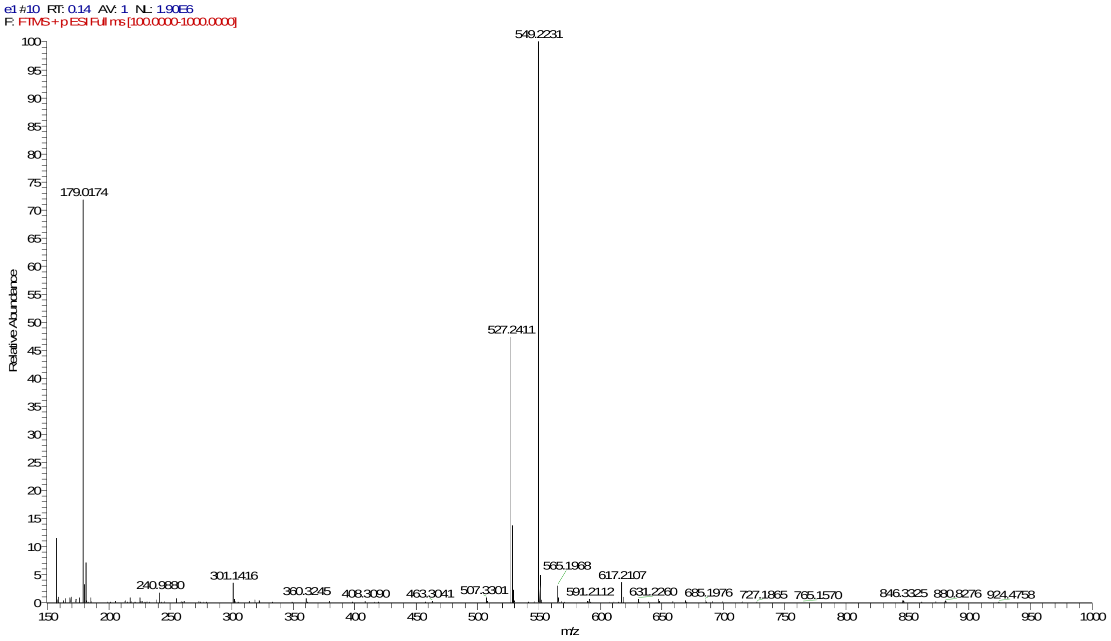

Figure S2-1. ^{1}H NMR spectrum (600MHz, DMSO-d_6) of compound 3b

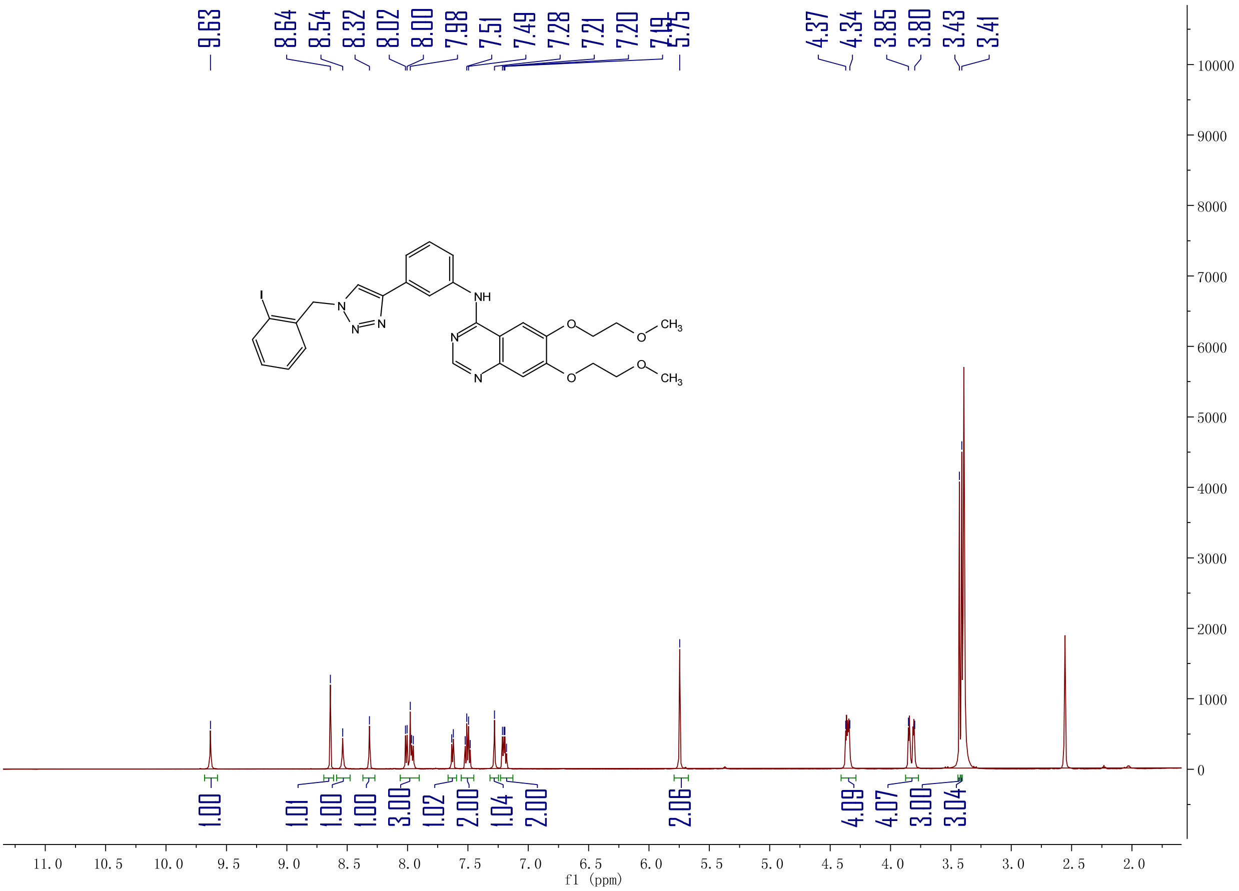

Figure S2-2. ^{13}C NMR spectrum (150MHz, DMSO-d_6) of compound 3b

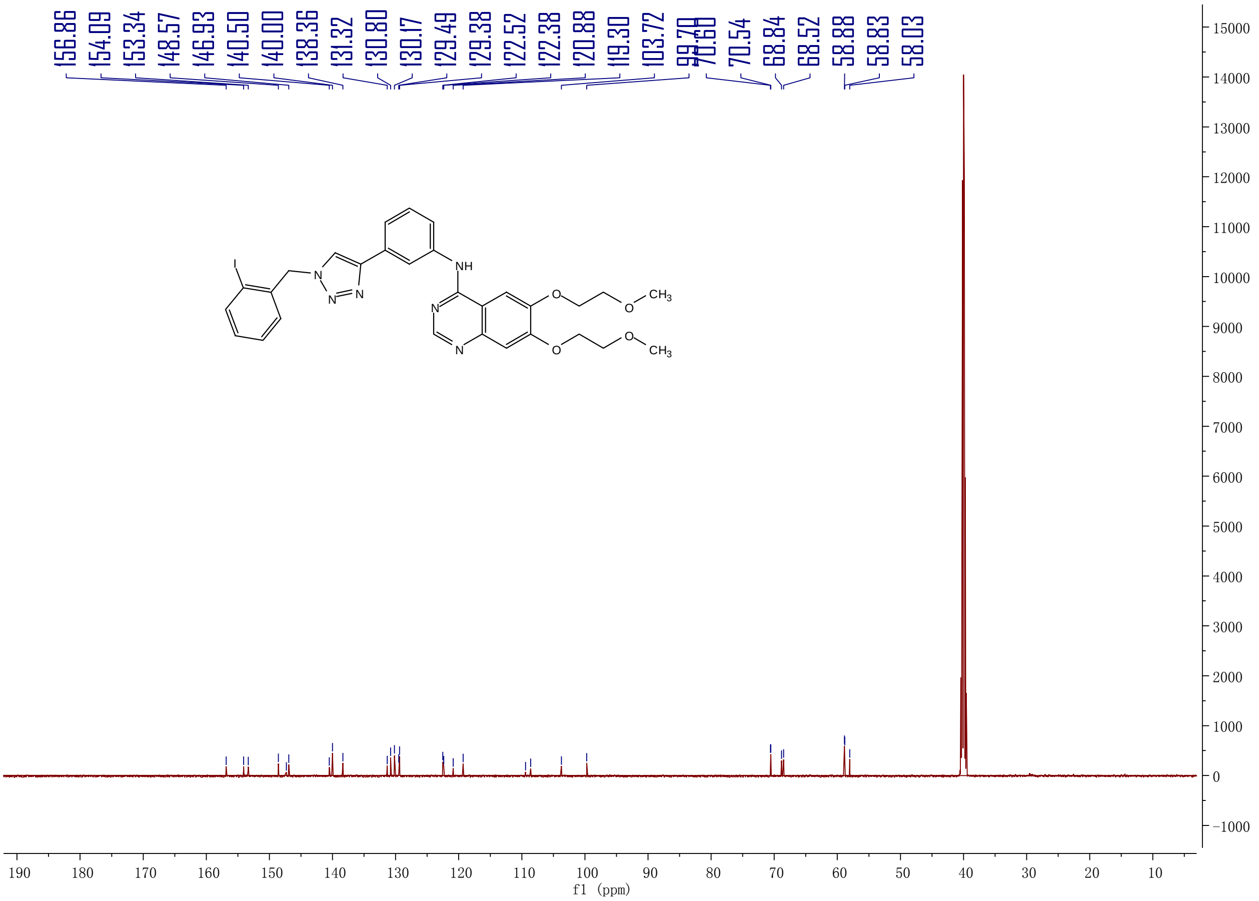

Figure S2-3. HR MS of compound 3b

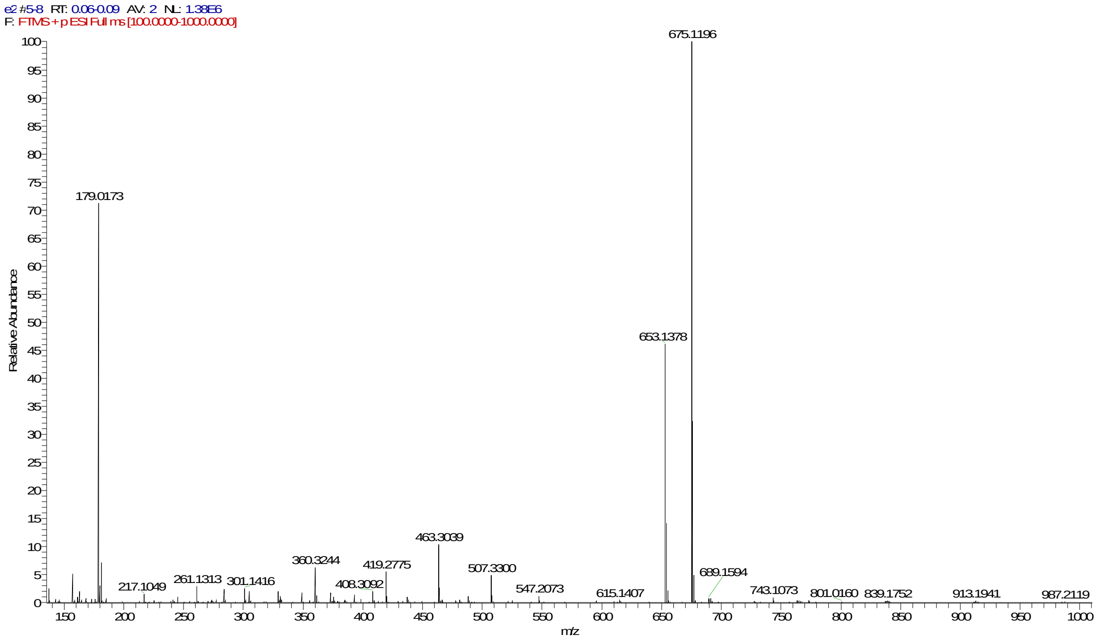

Figure S3-1. ^{1}H NMR spectrum (600MHz, DMSO-d$_6$) of compound 3c

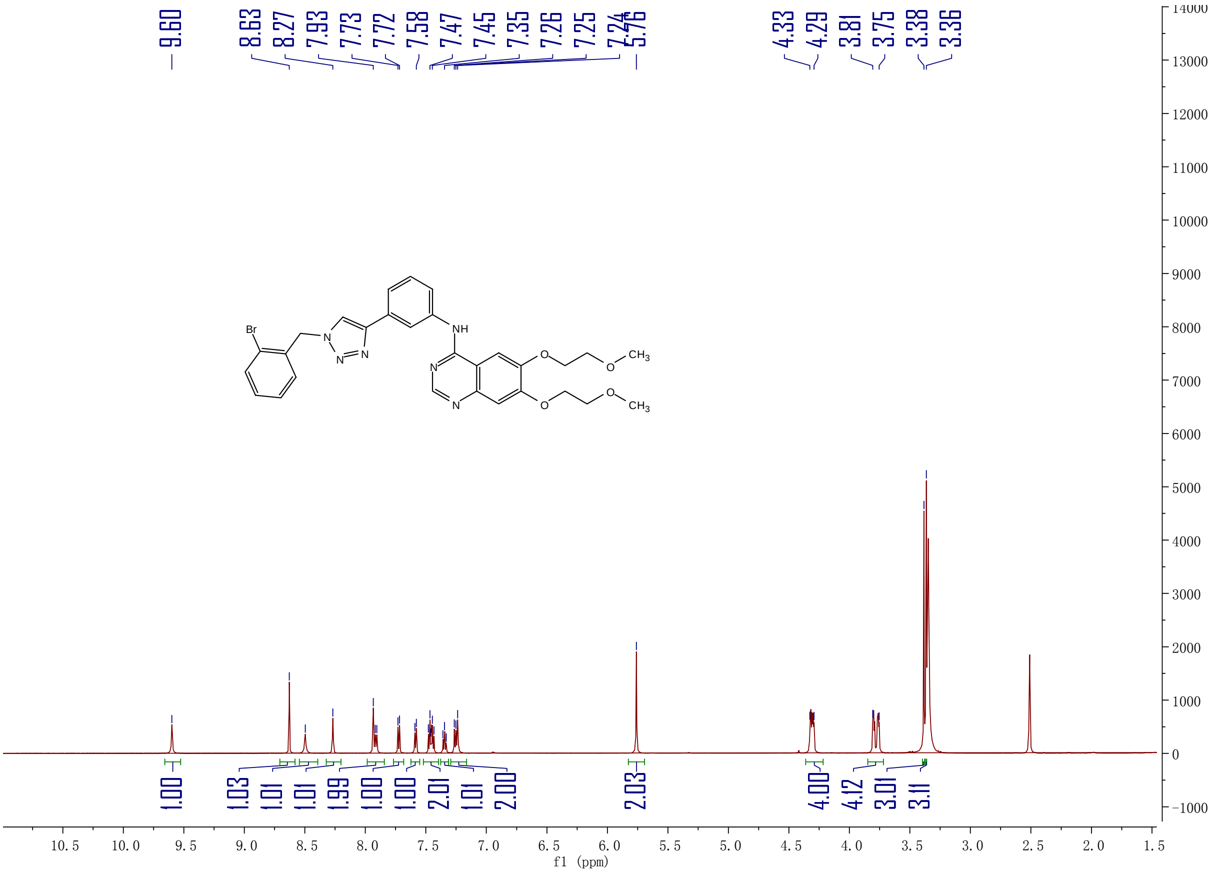

Figure S3-2. ^{13}C NMR spectrum (150MHz, DMSO-d_6) of compound 3c

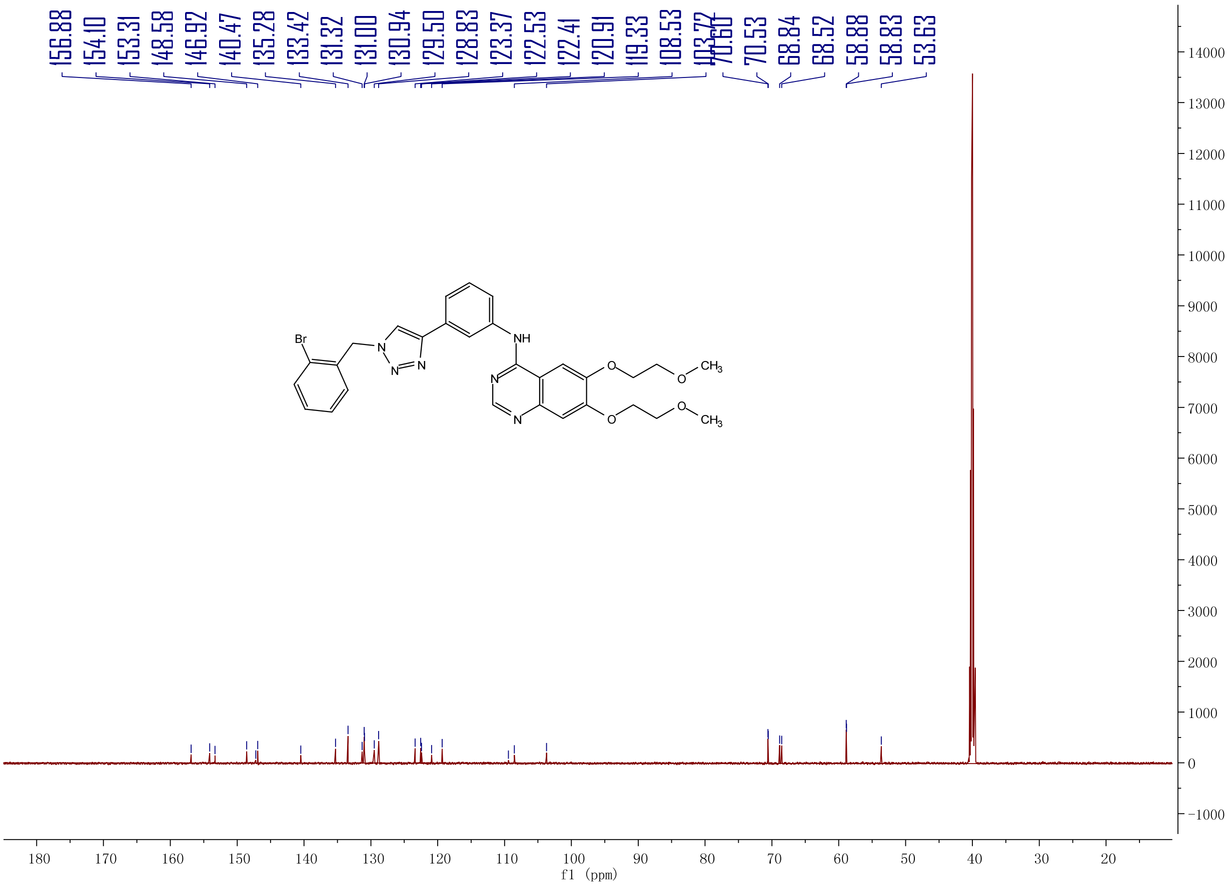

Figure S3-3. HR MS of compound 3c

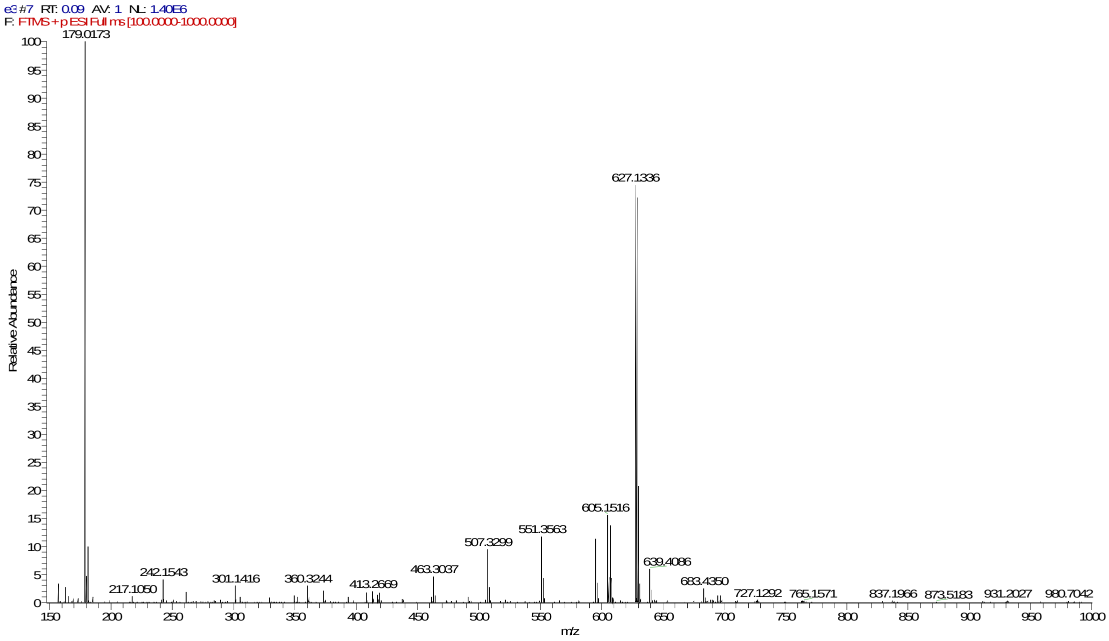

Figure S4-1. ^{1}H NMR spectrum (600MHz, DMSO-d$_6$) of compound 3d

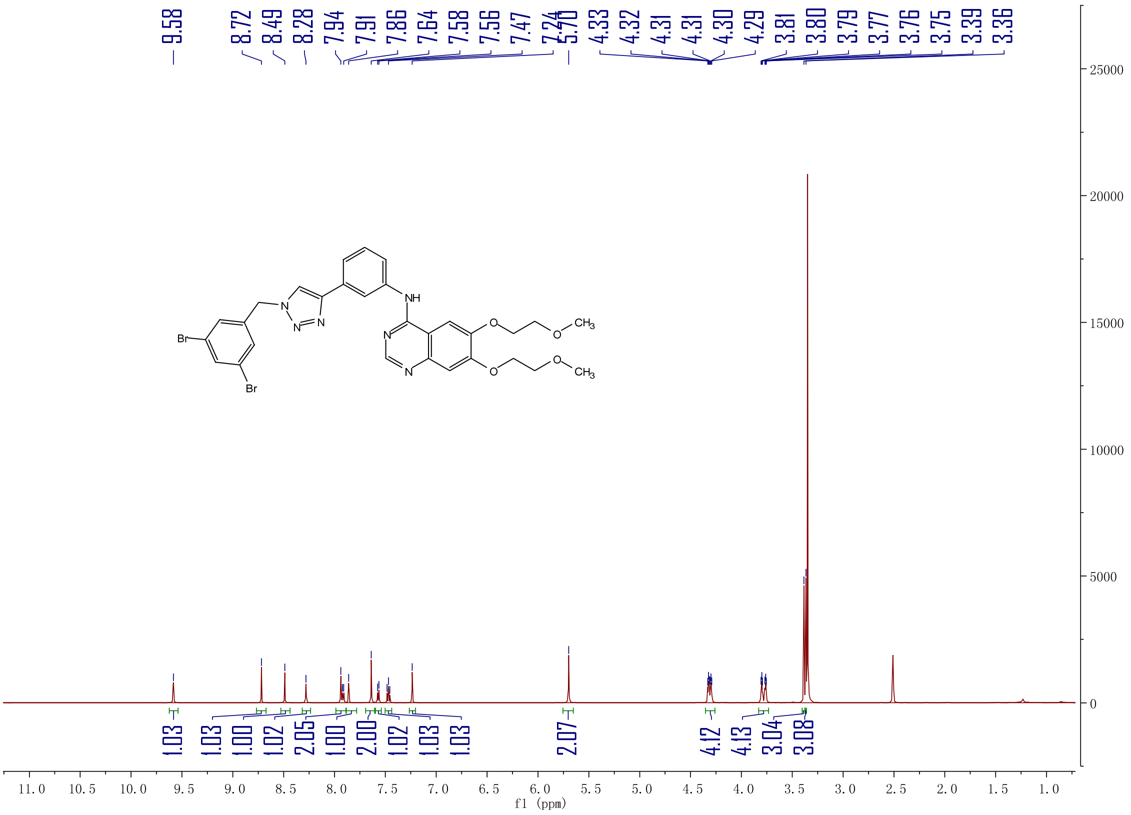

Figure S4-2. ^{13}C NMR spectrum (150MHz, DMSO-d_6) of compound 3d

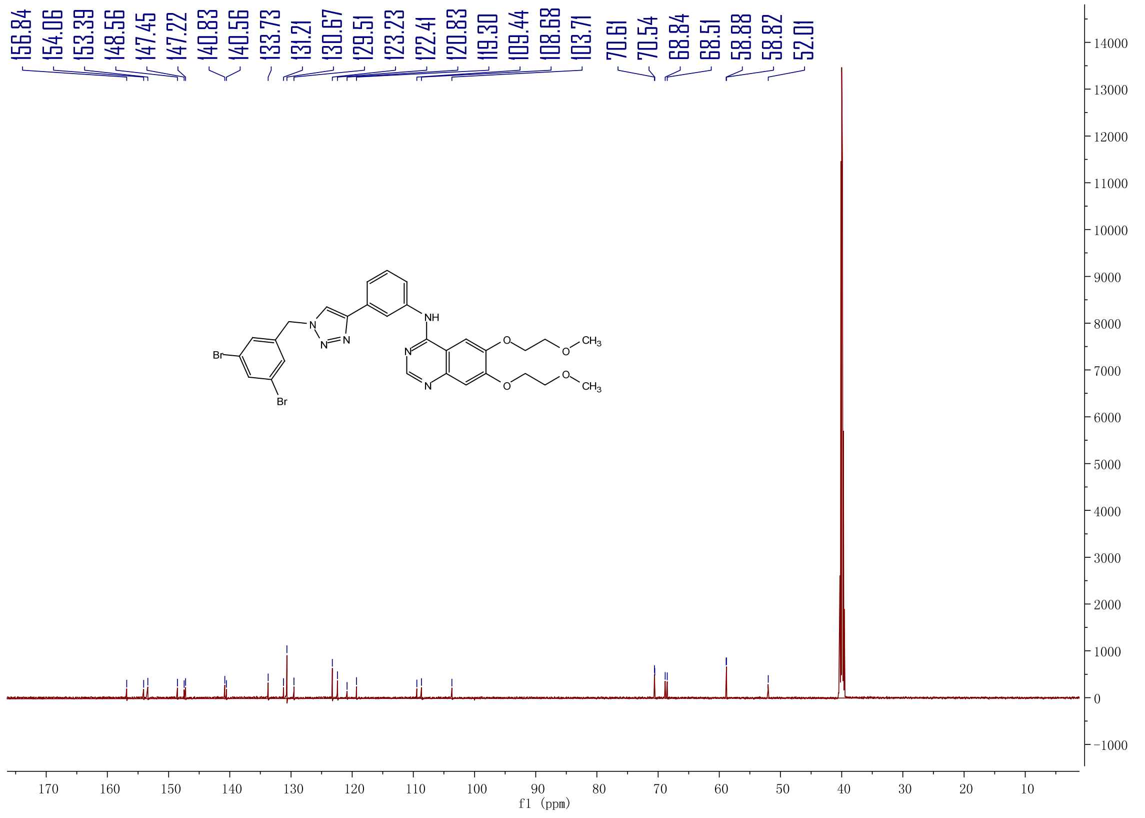

Figure S4-3. HR MS of compound 3d

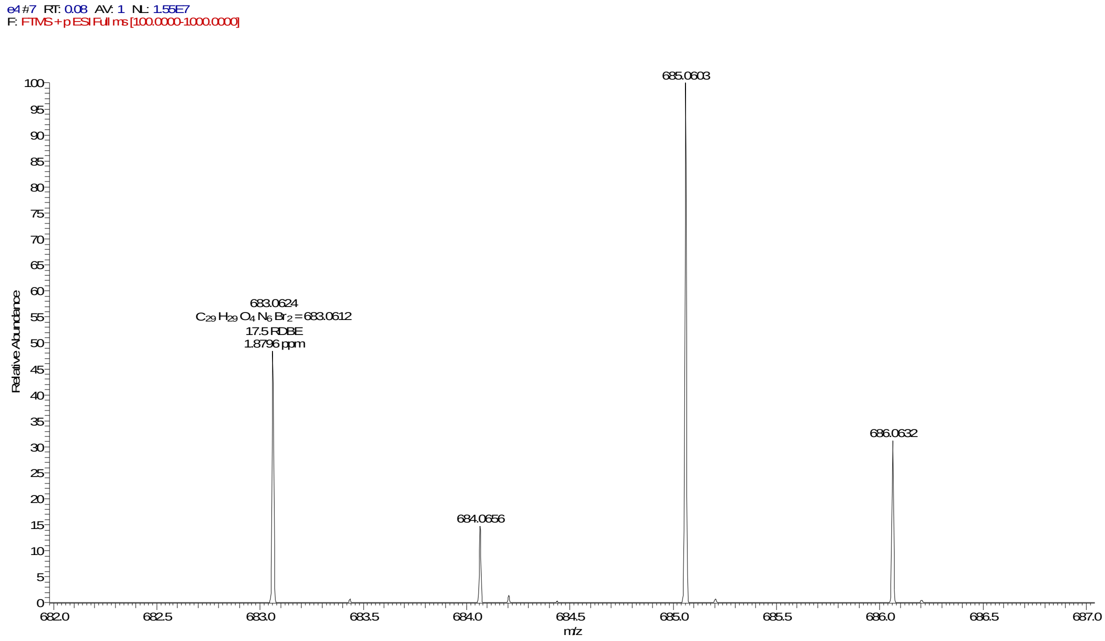

Figure S5-1. ^{1}H NMR spectrum (600MHz, DMSO-d$_6$) of compound 3e

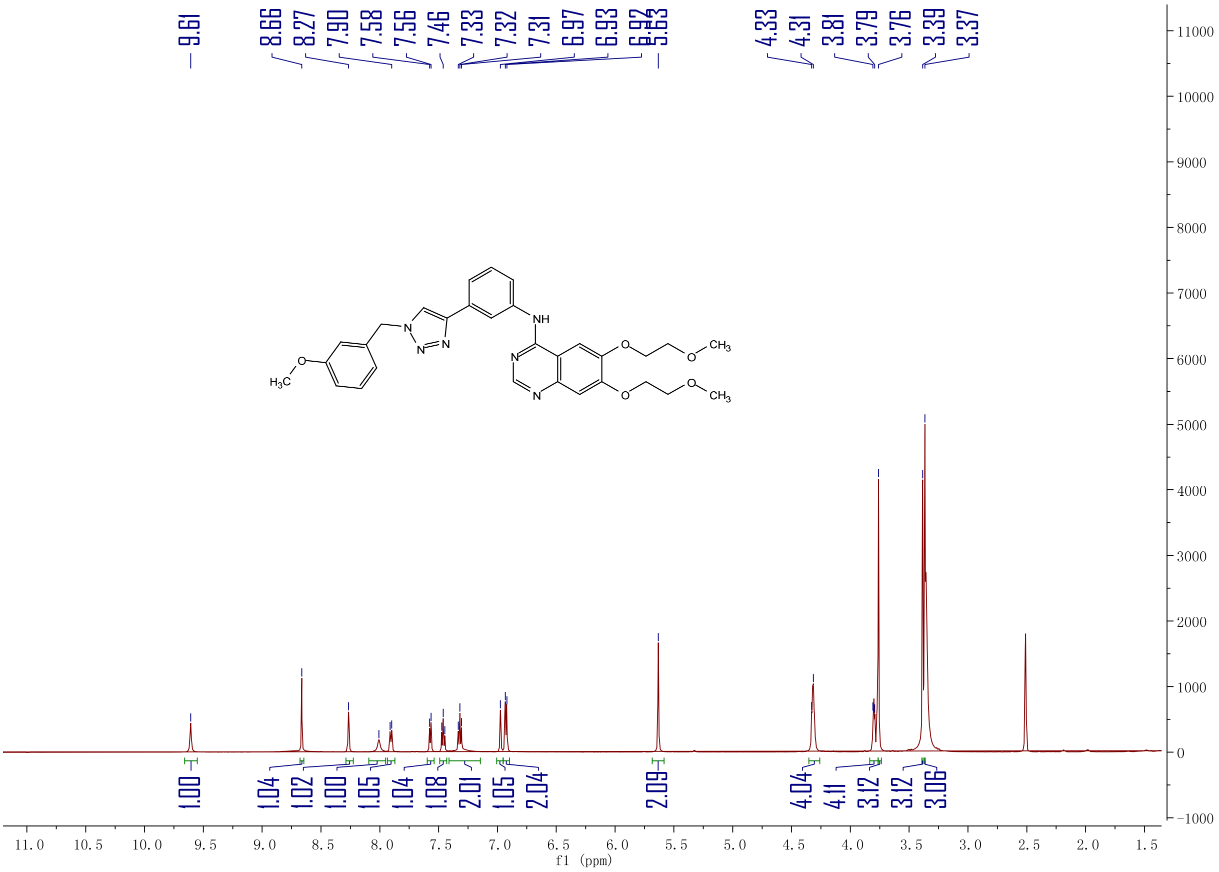

Figure S5-2. ^{13}C NMR spectrum (150MHz, DMSO-d_6) of compound 3e

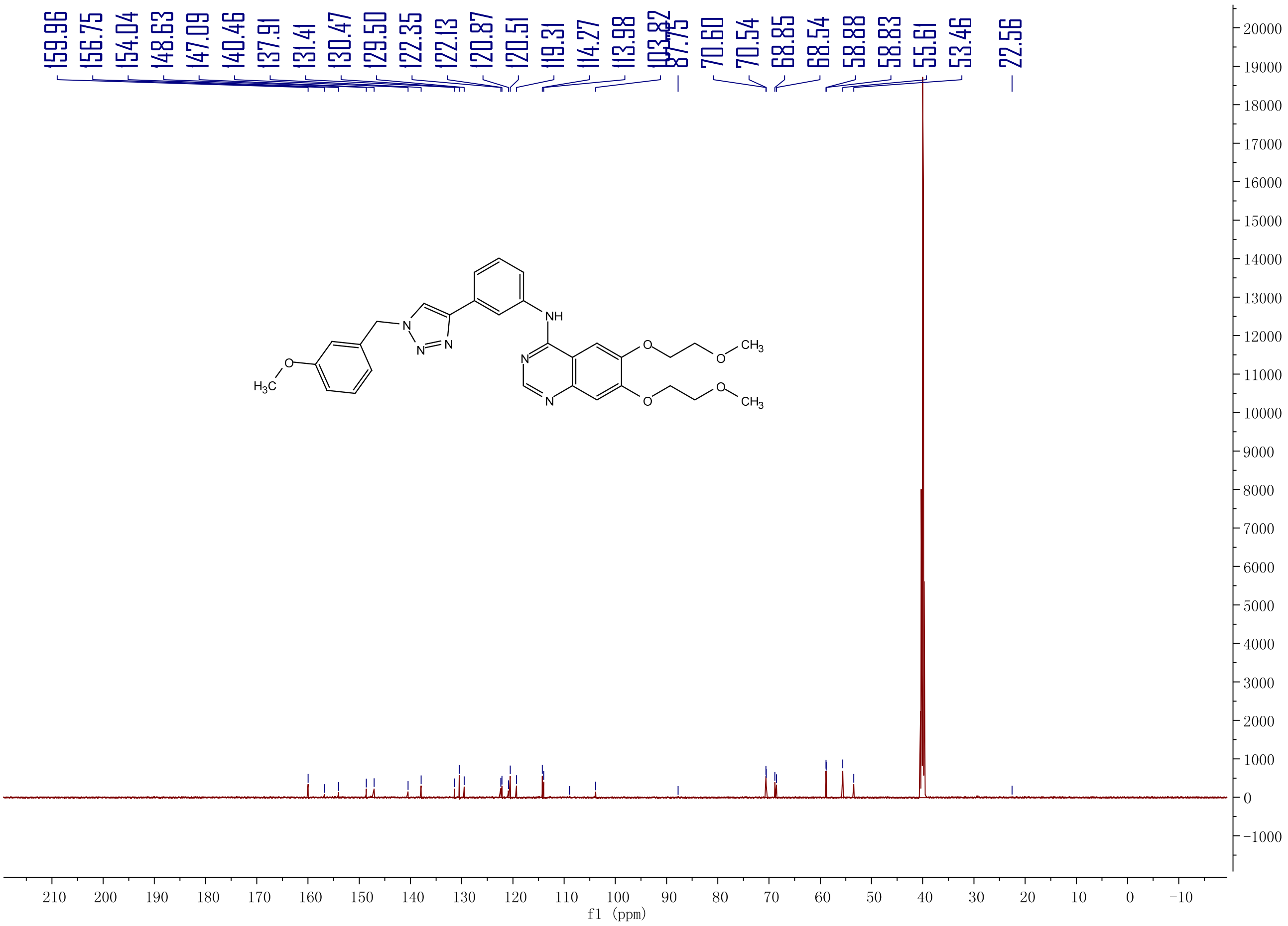

Figure S5-3. HR MS of compound 3e

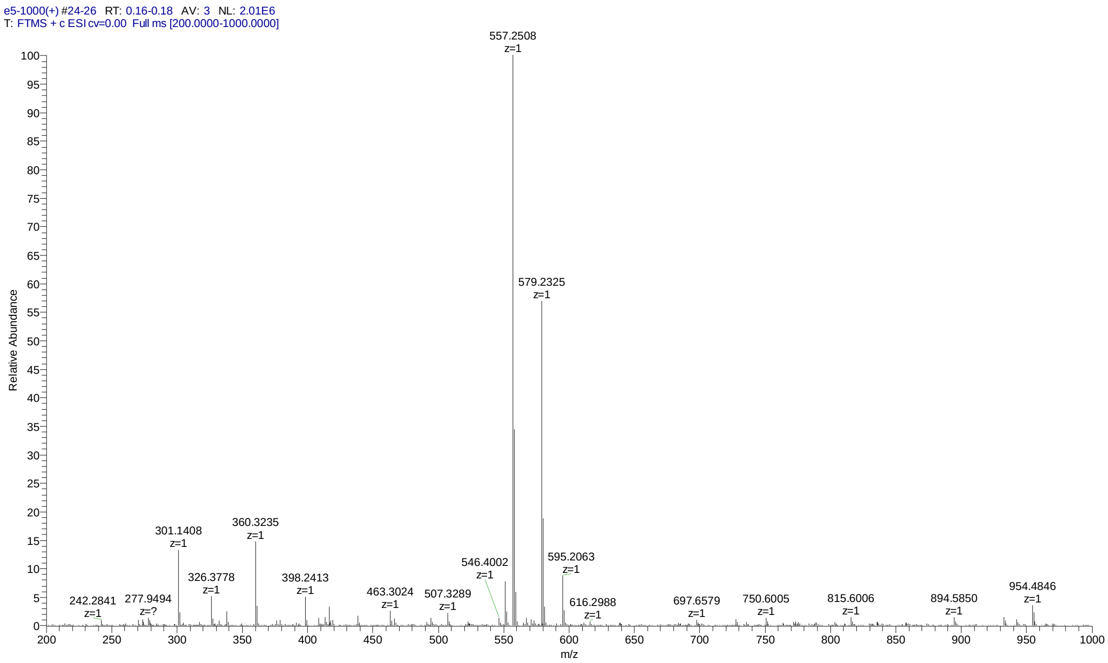

Figure S6-1. ^{1}H NMR spectrum (600MHz, DMSO-d_6) of compound 3f

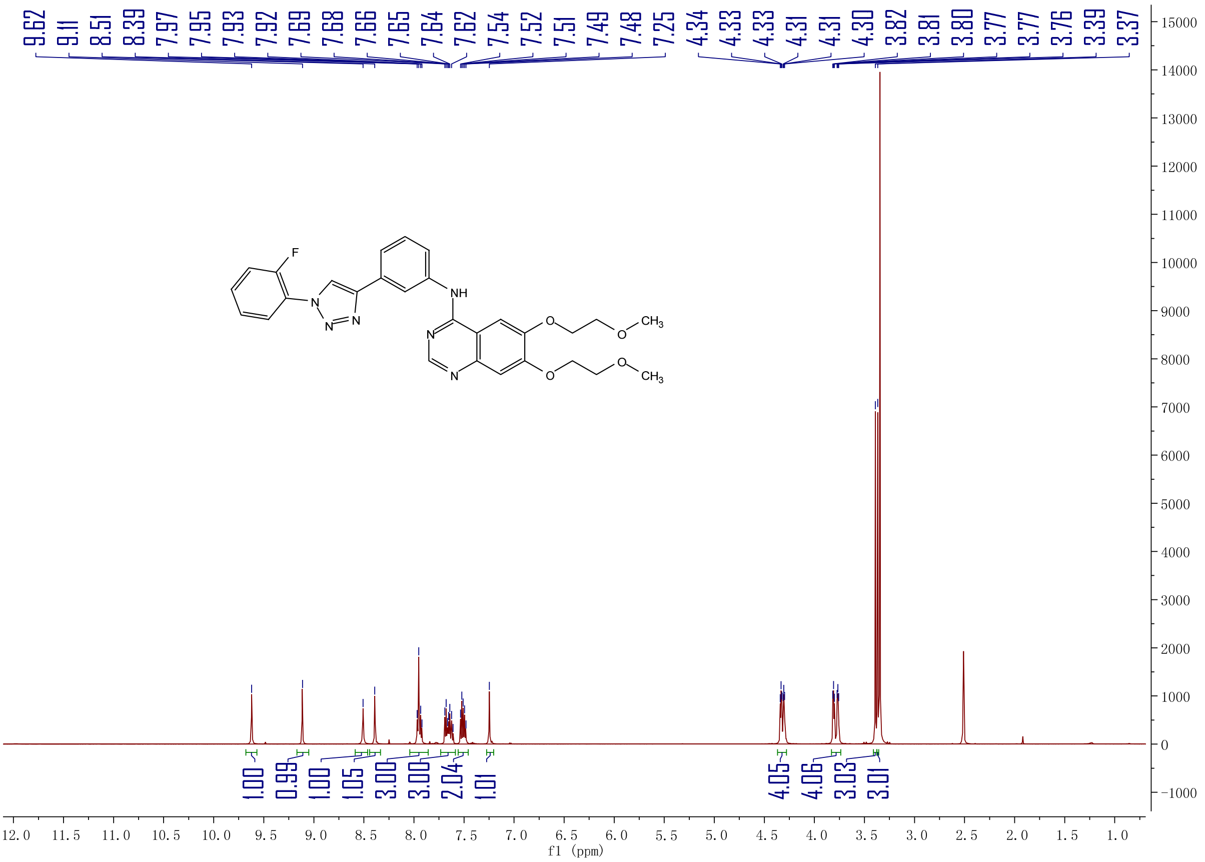

Figure S6-2. ^{13}C NMR spectrum (150MHz, DMSO-d_6) of compound 3f

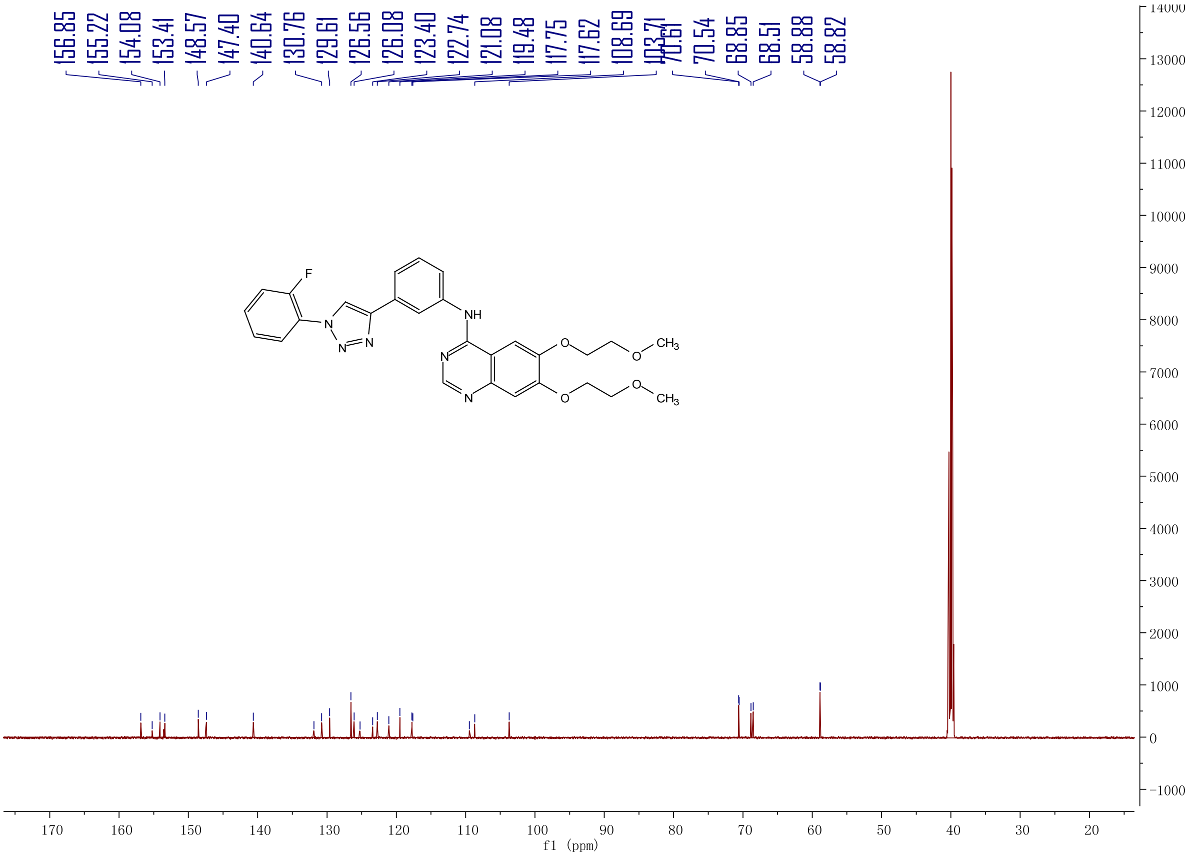

Figure S6-3. HR MS of compound 3f

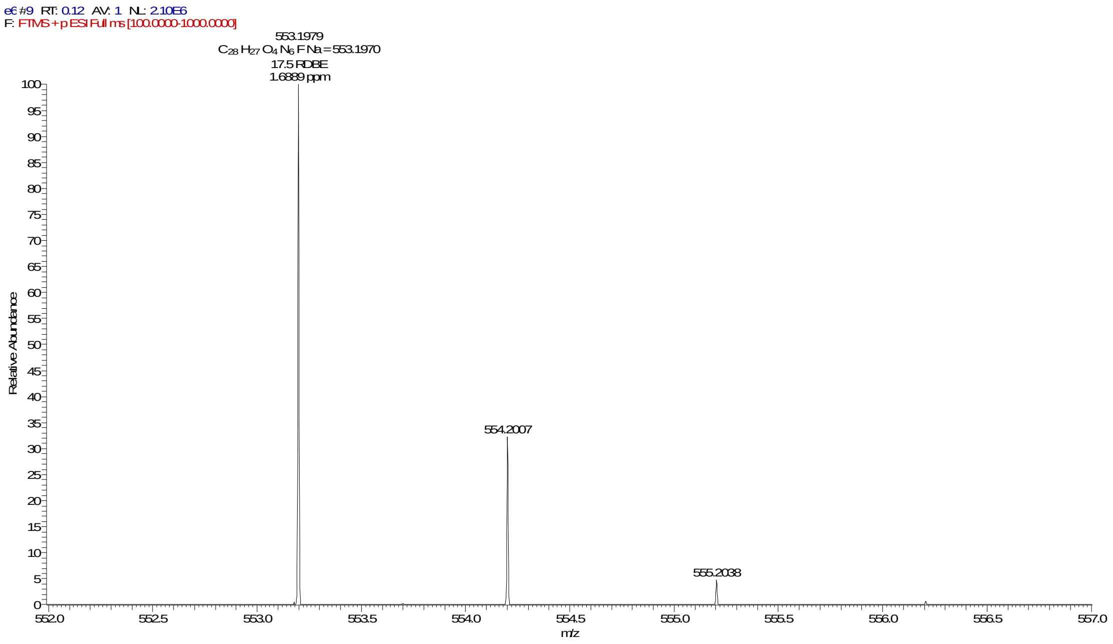

Figure S7-1. ^{1}H NMR spectrum (600MHz, DMSO-d_6) of compound 3g

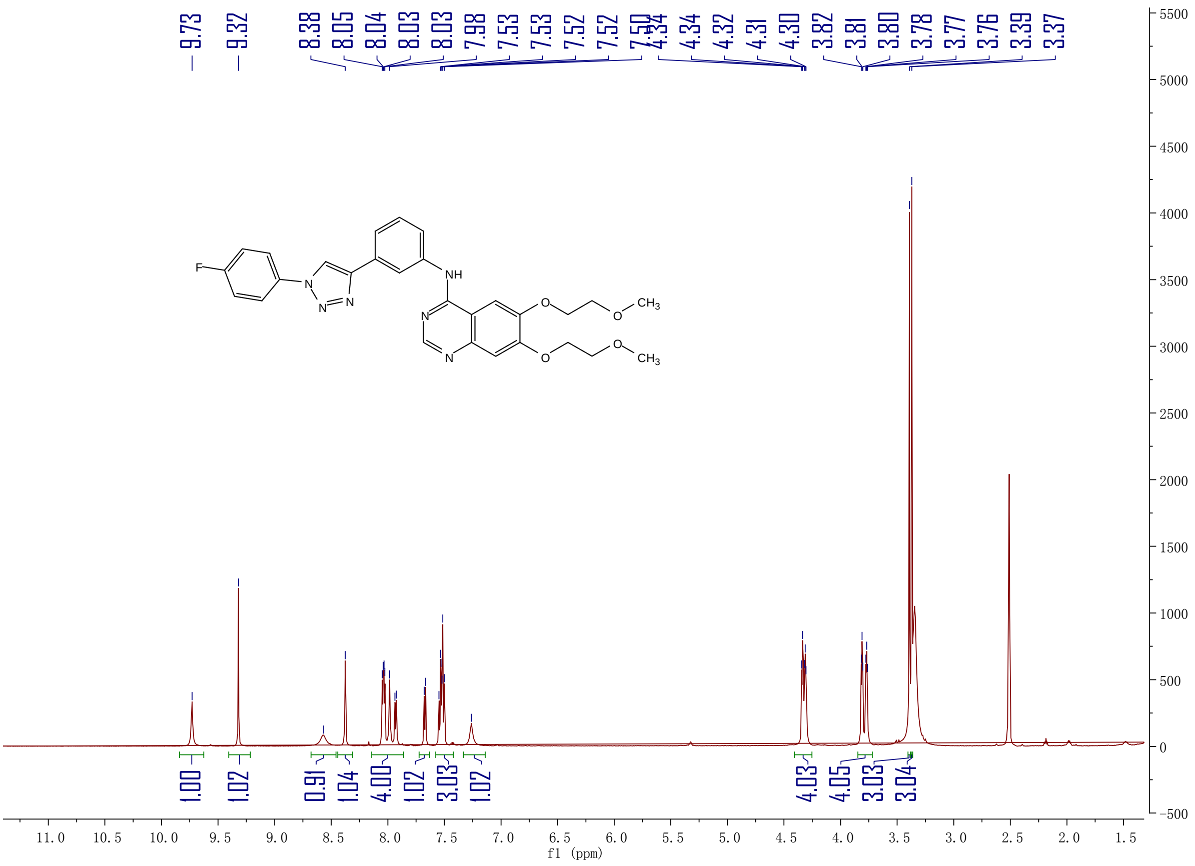

Figure S7-2. ^{13}C NMR spectrum (150MHz, DMSO-d$_6$) of compound 3g

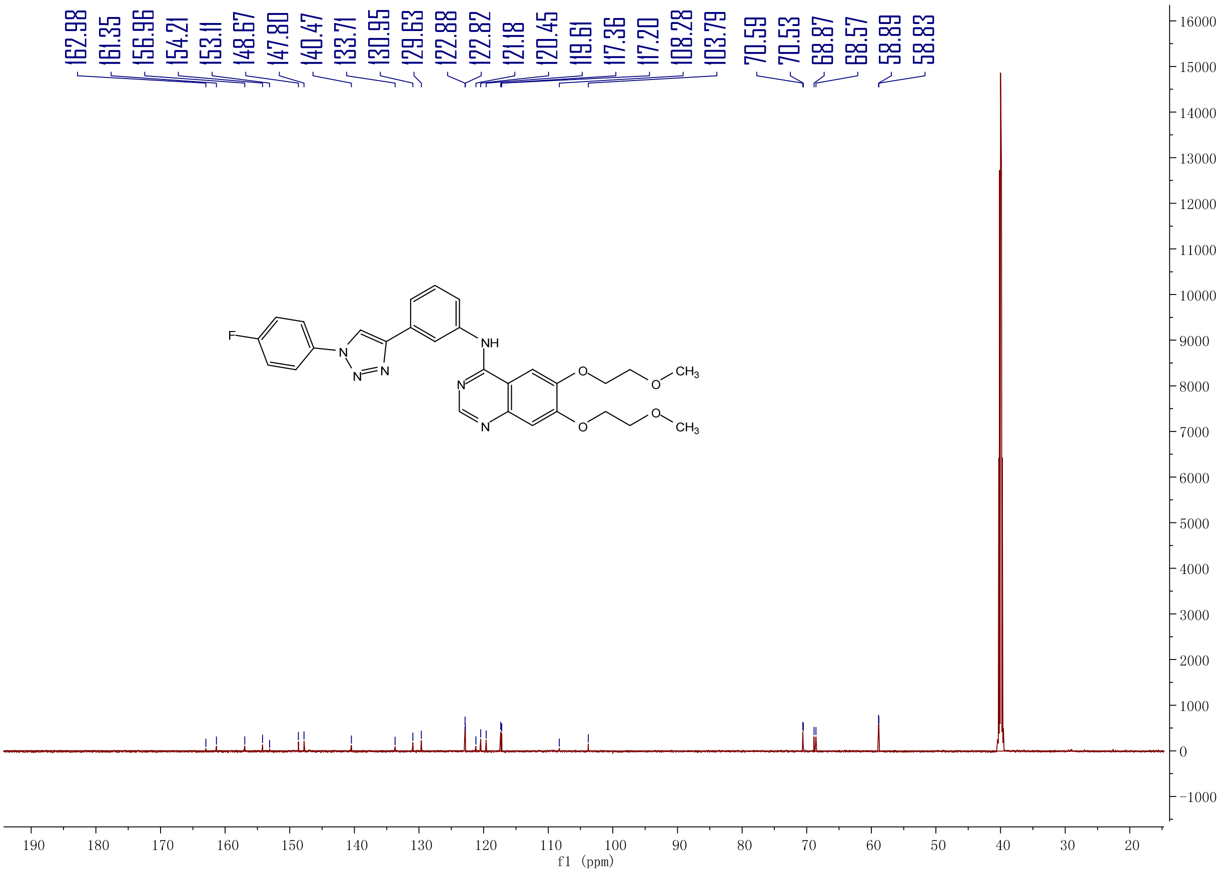

Figure S7-3. HR MS of compound 3g

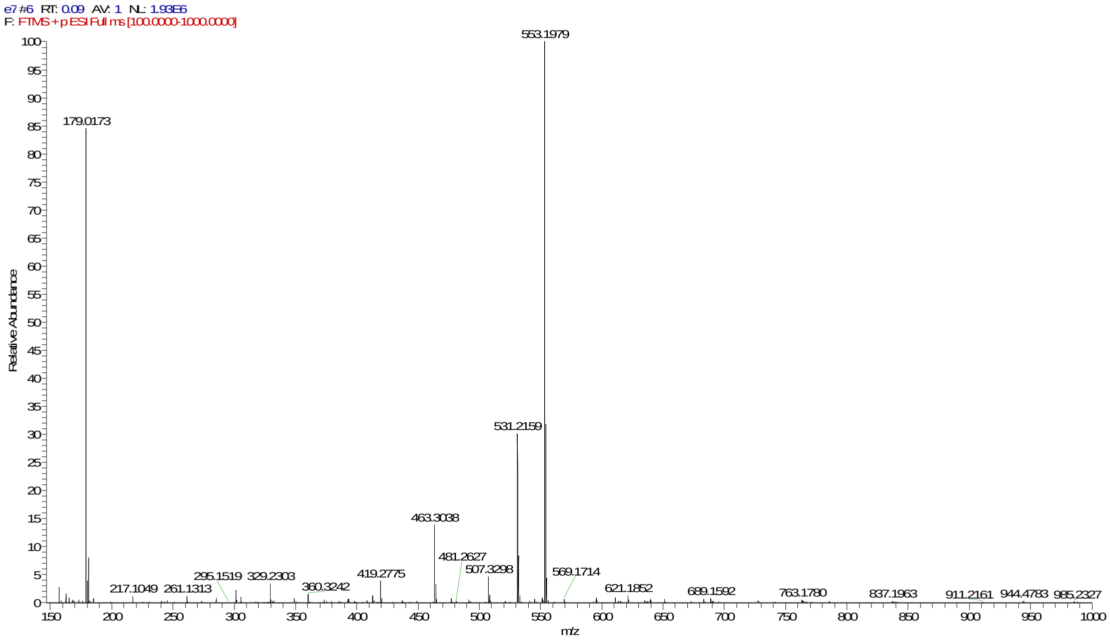

Figure S8-1. ^{1}H NMR spectrum (600MHz, DMSO-d$_6$) of compound 3h

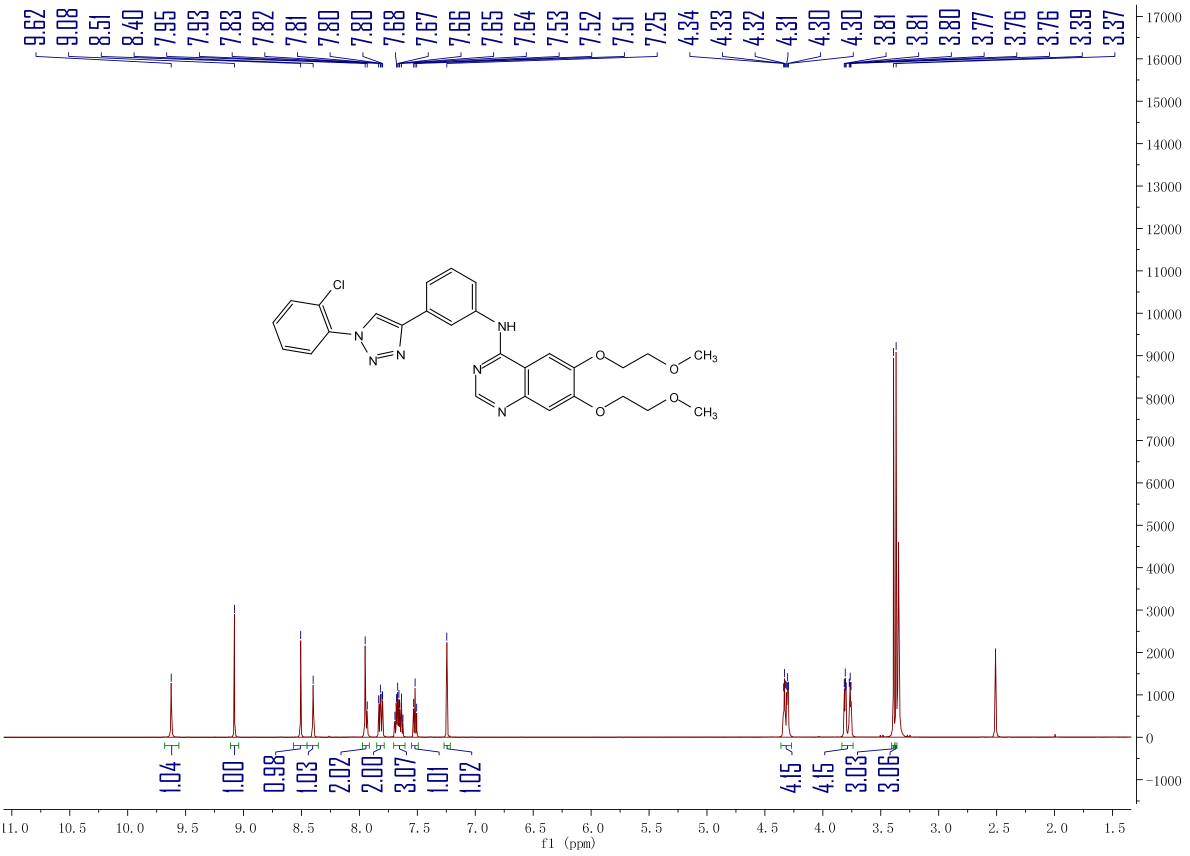

Figure S8-2. ^{13}C NMR spectrum (150MHz, DMSO-d_6) of compound 3h

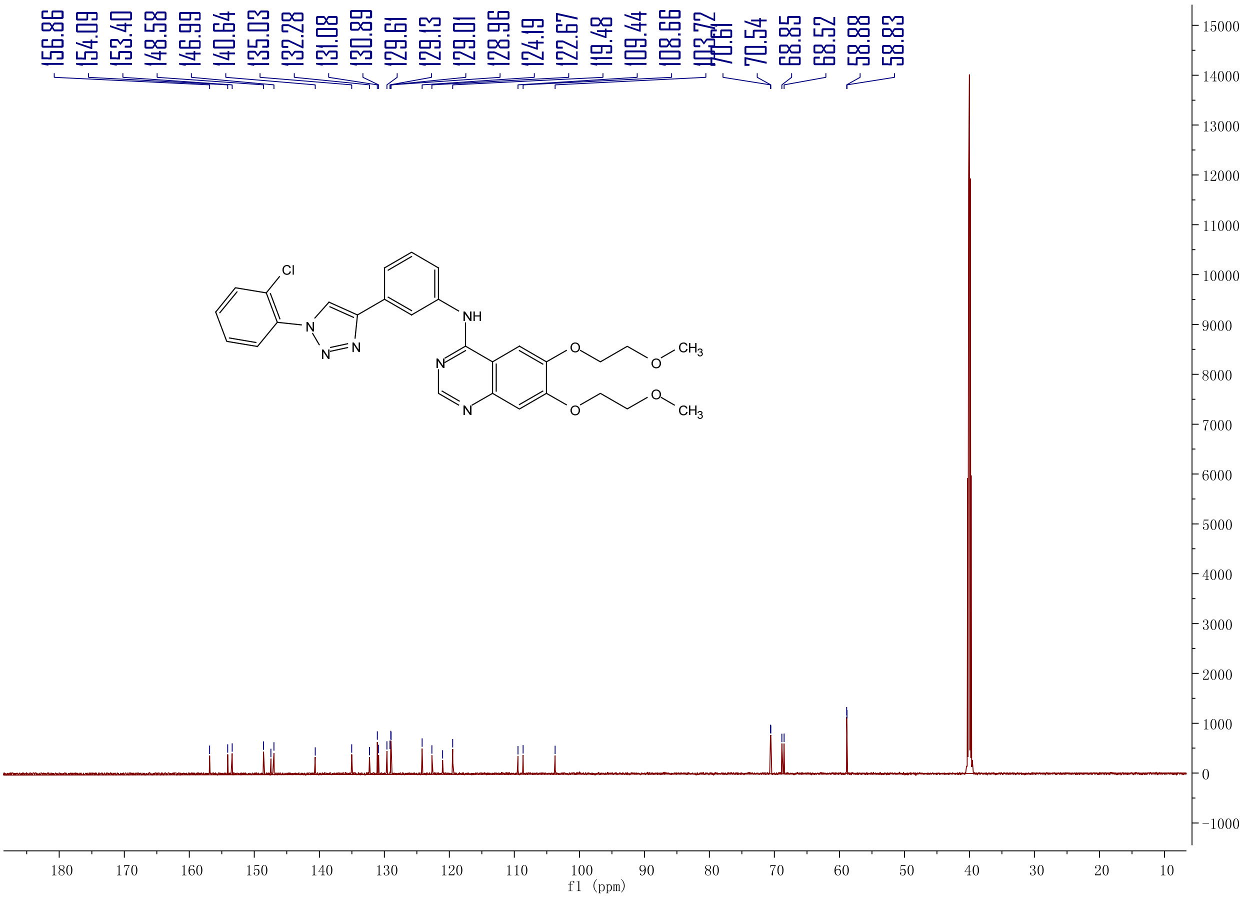

Figure S8-3. HR MS of compound 3h

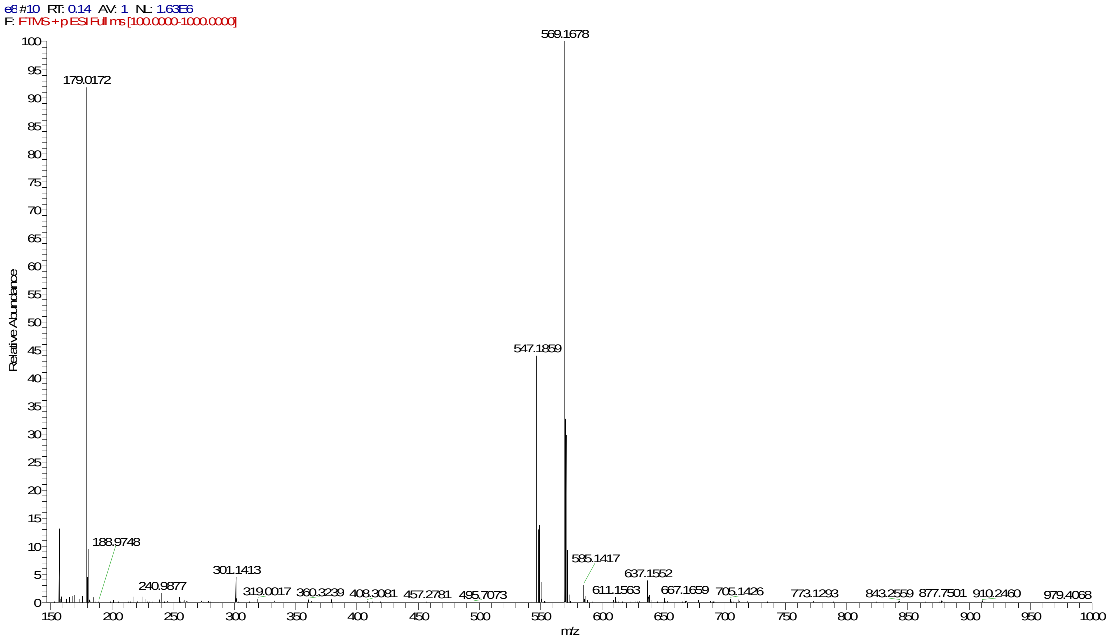

Figure S9-1. ^{1}H NMR spectrum (600MHz, DMSO-d_6) of compound 3i

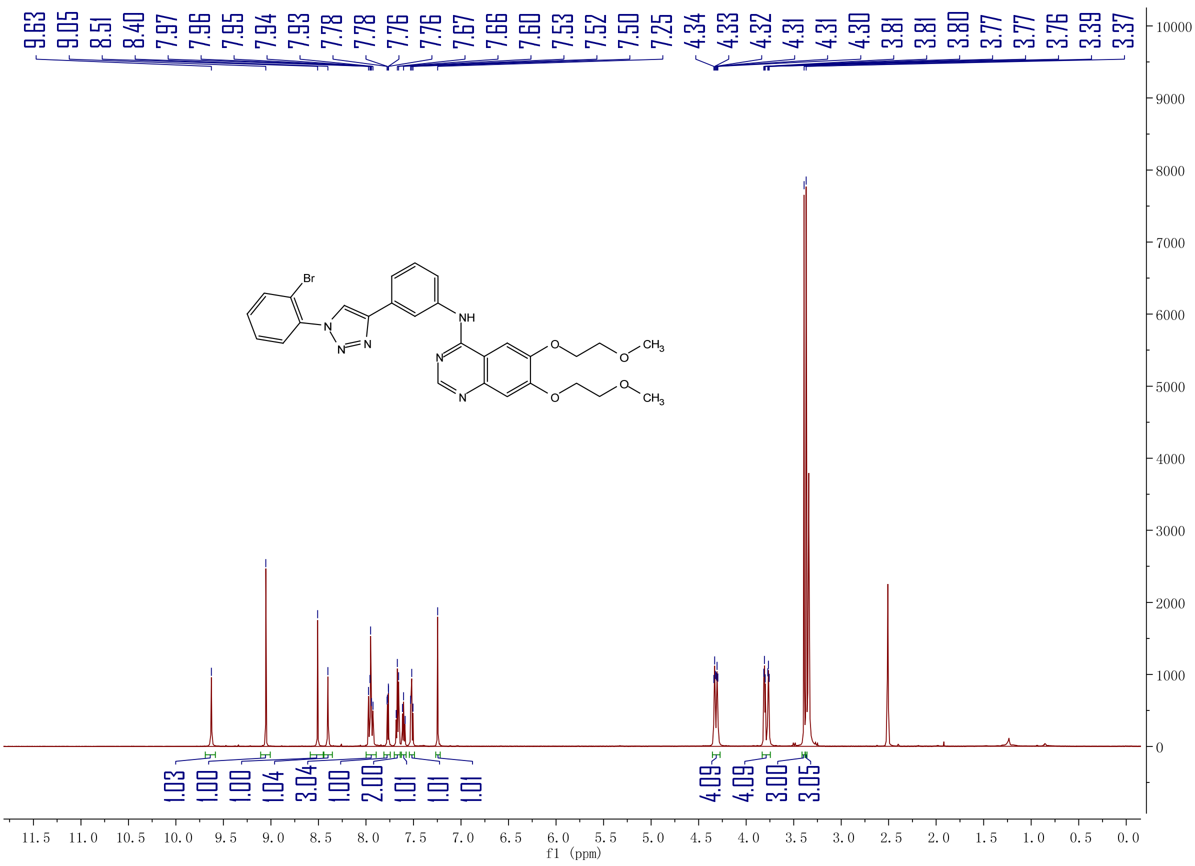

Figure S9-2. ^{13}C NMR spectrum (150MHz, DMSO-d_6) of compound 3i

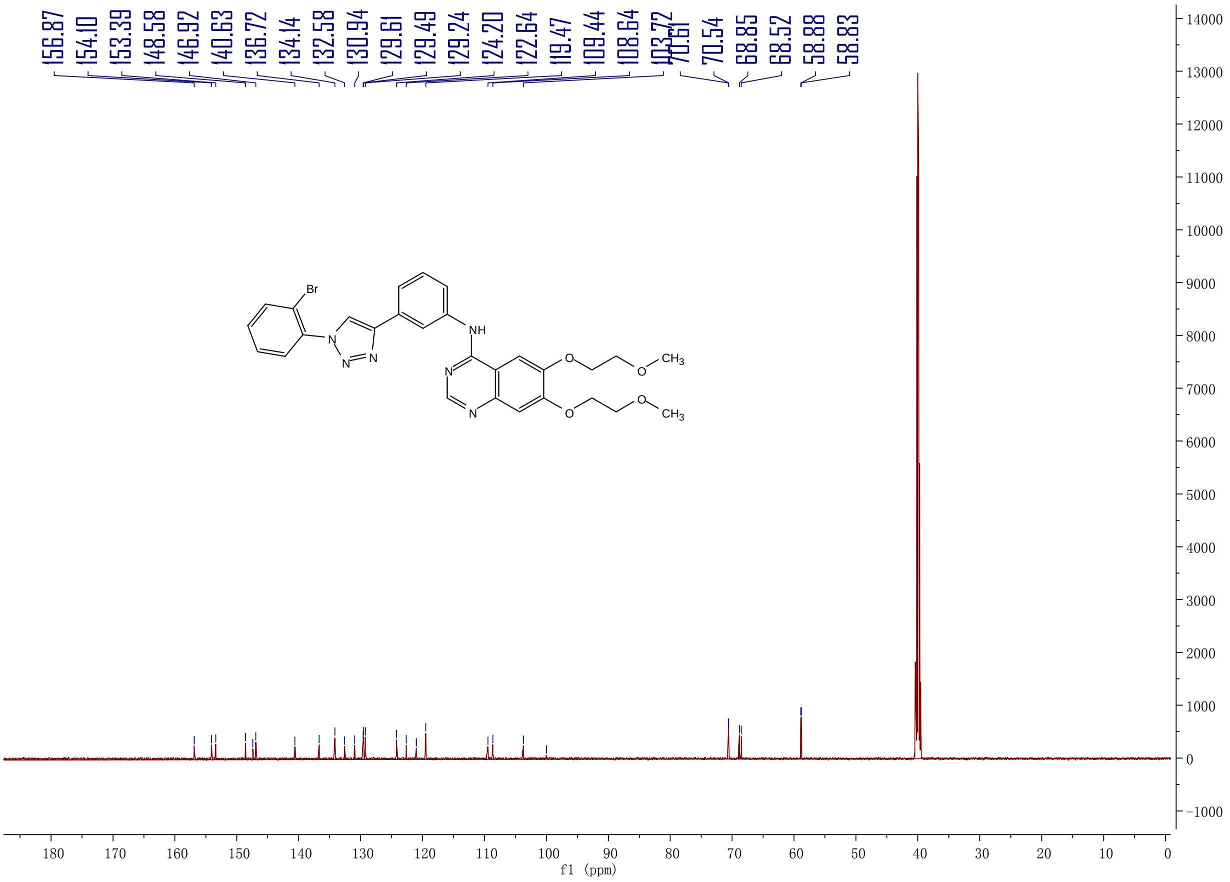

Figure S9-3. HR MS of compound 3i

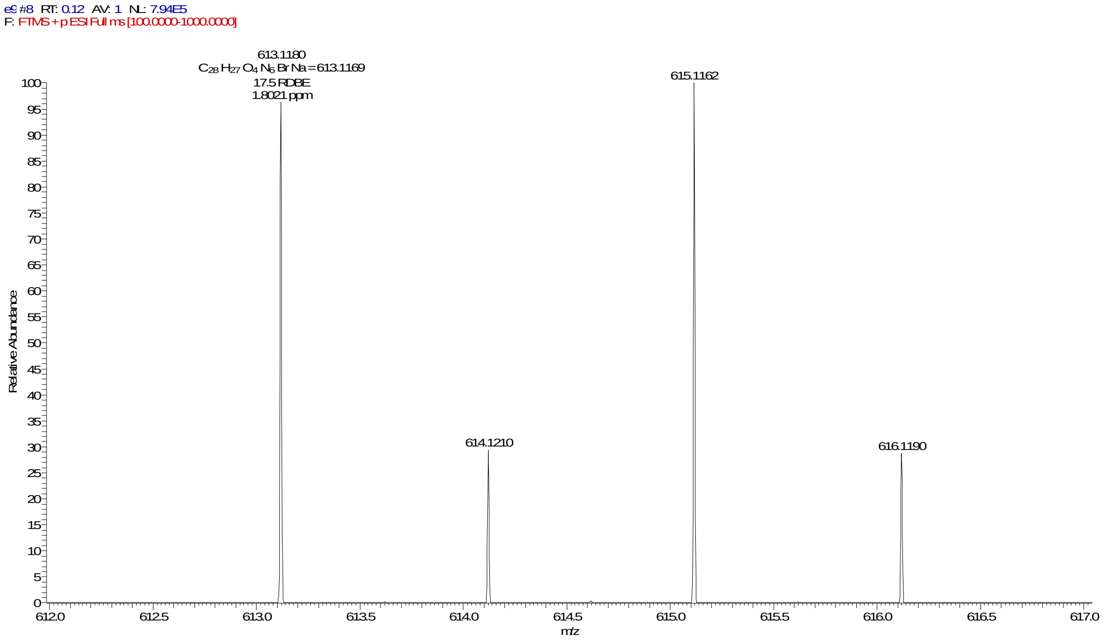

Figure S10-1. ^{1}H NMR spectrum (600MHz, DMSO-d_6) of compound 3j

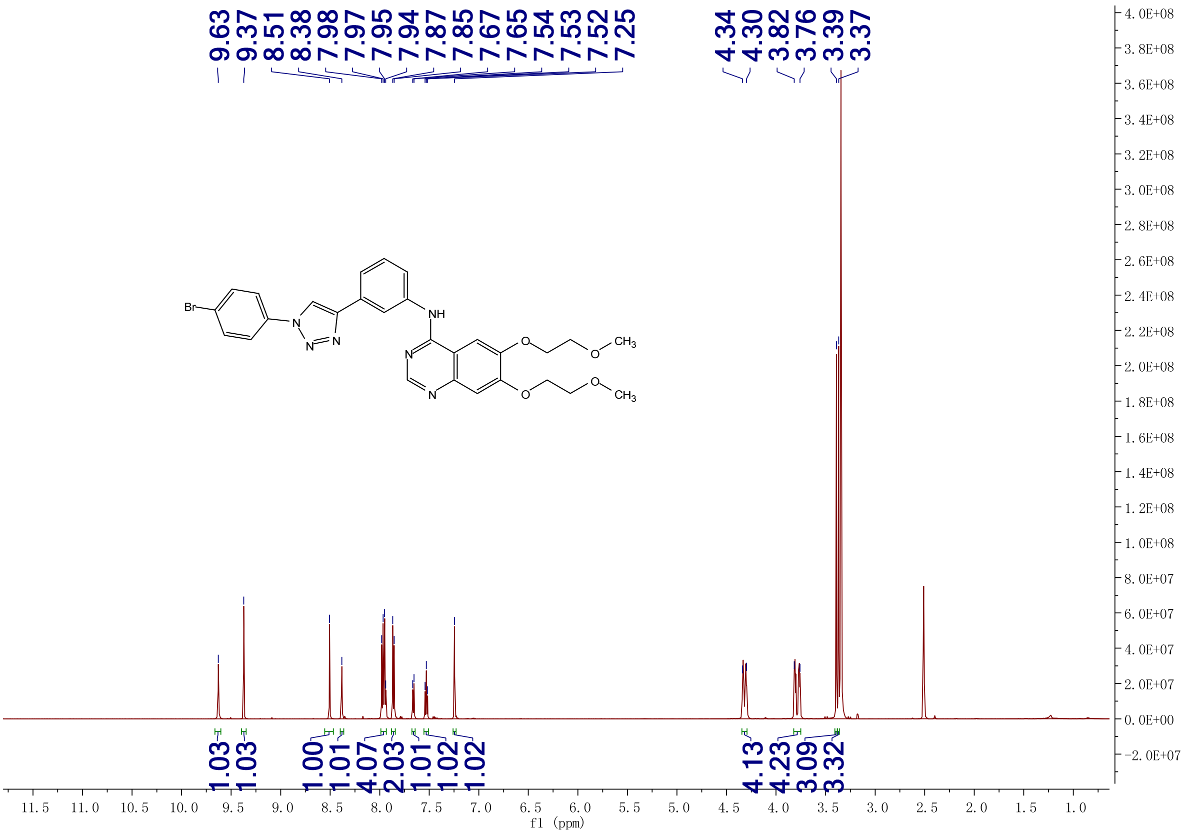

Figure S10-2. ^{13}C NMR spectrum (150MHz, DMSO-d$_6$) of compound 3j

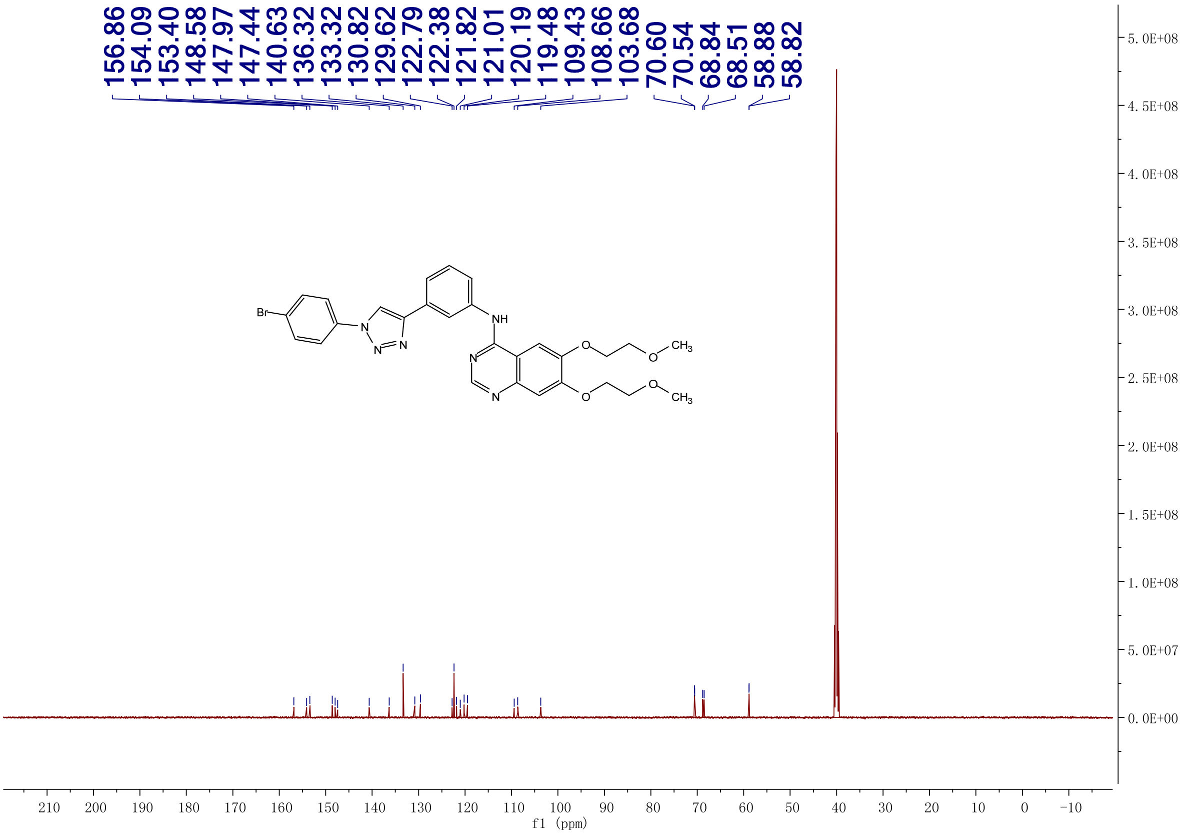

Figure S10-3. HR MS of compound 3j

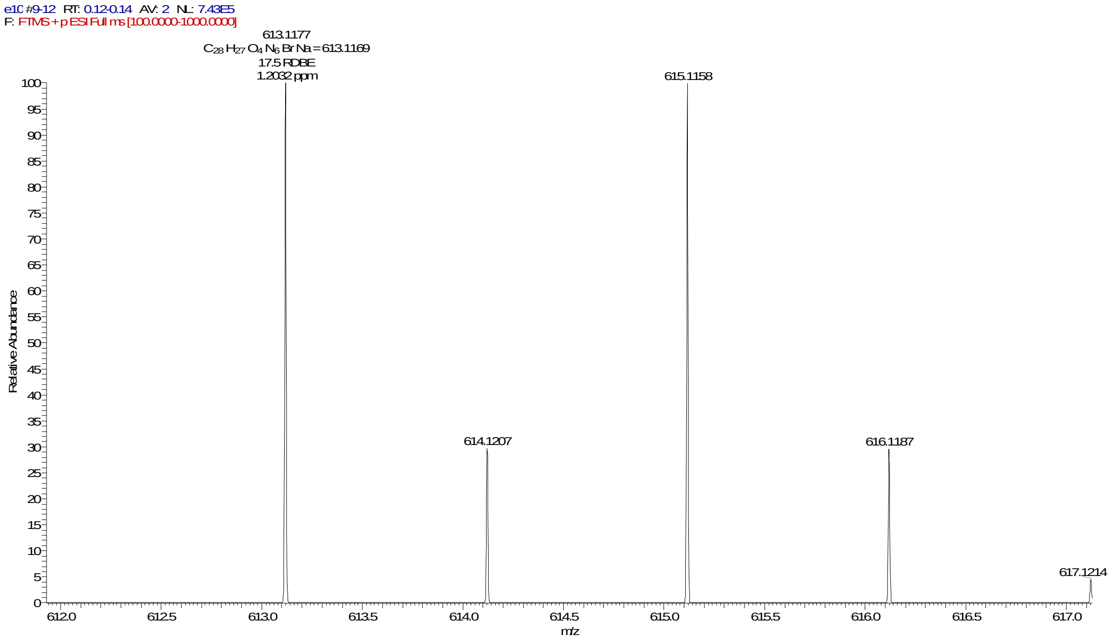

Figure S11-1. ^{1}H NMR spectrum (600MHz, DMSO-d_6) of compound 3k

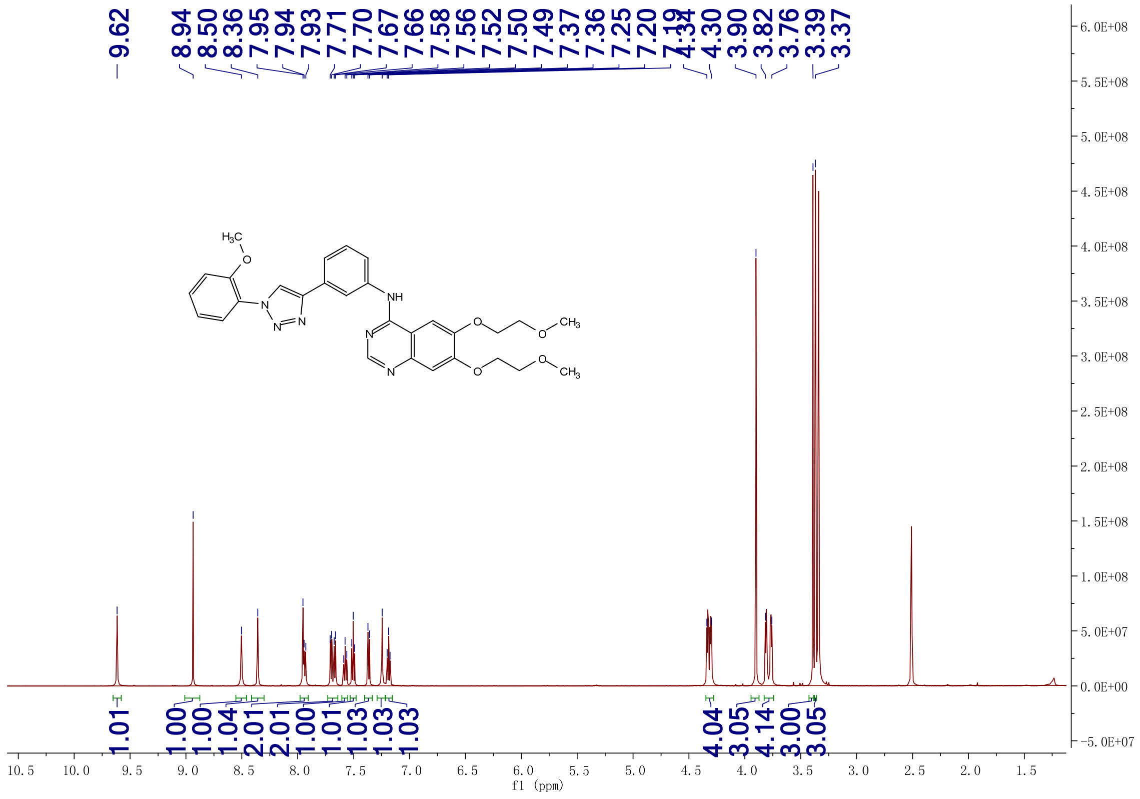

Figure S11-2. ^{13}C NMR spectrum (150MHz, DMSO-d$_6$) of compound 3k

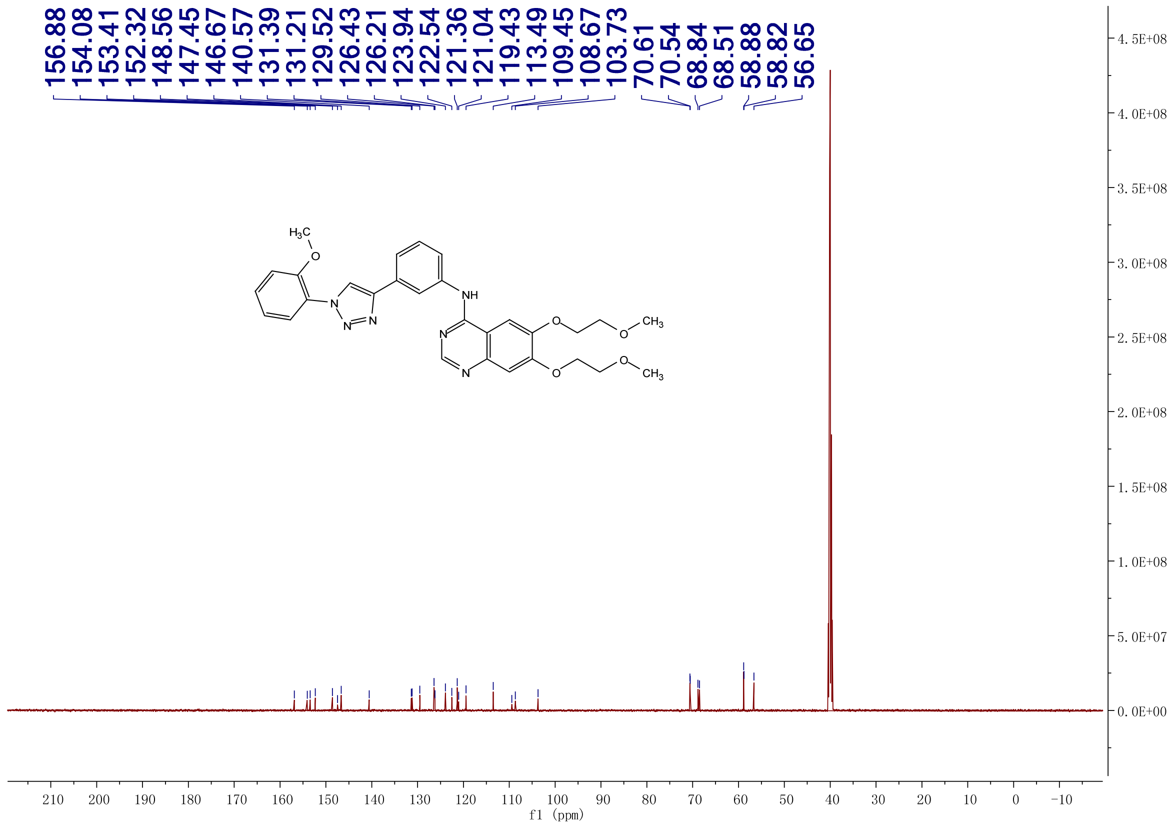

Figure S11-3. HR MS of compound 3k

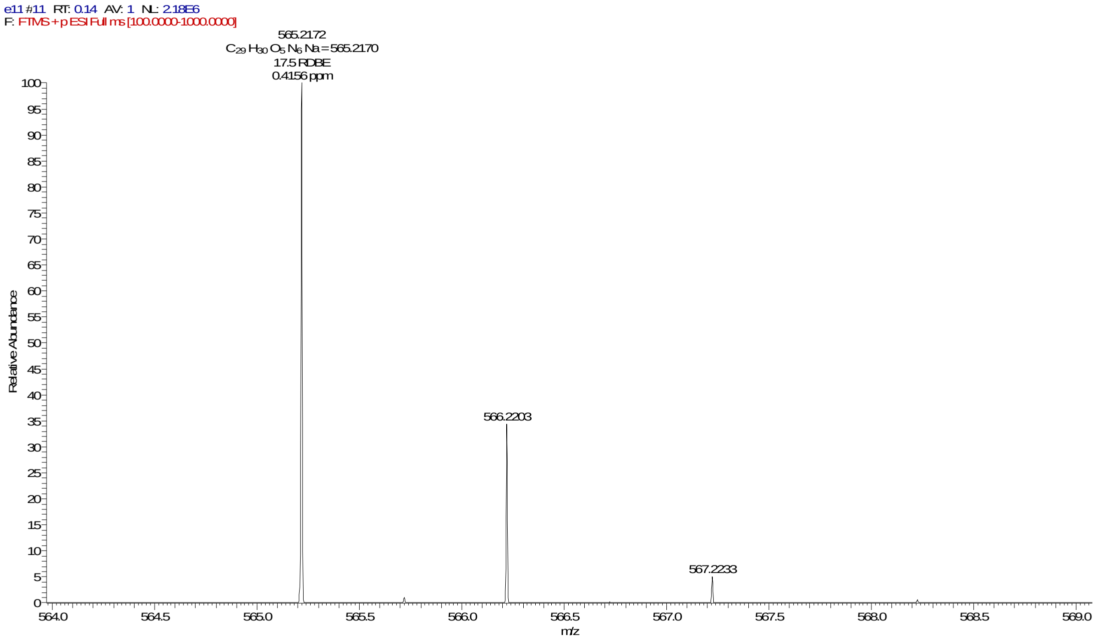

Figure S12-1. ^{1}H NMR spectrum (600MHz, DMSO-d_6) of compound 3l

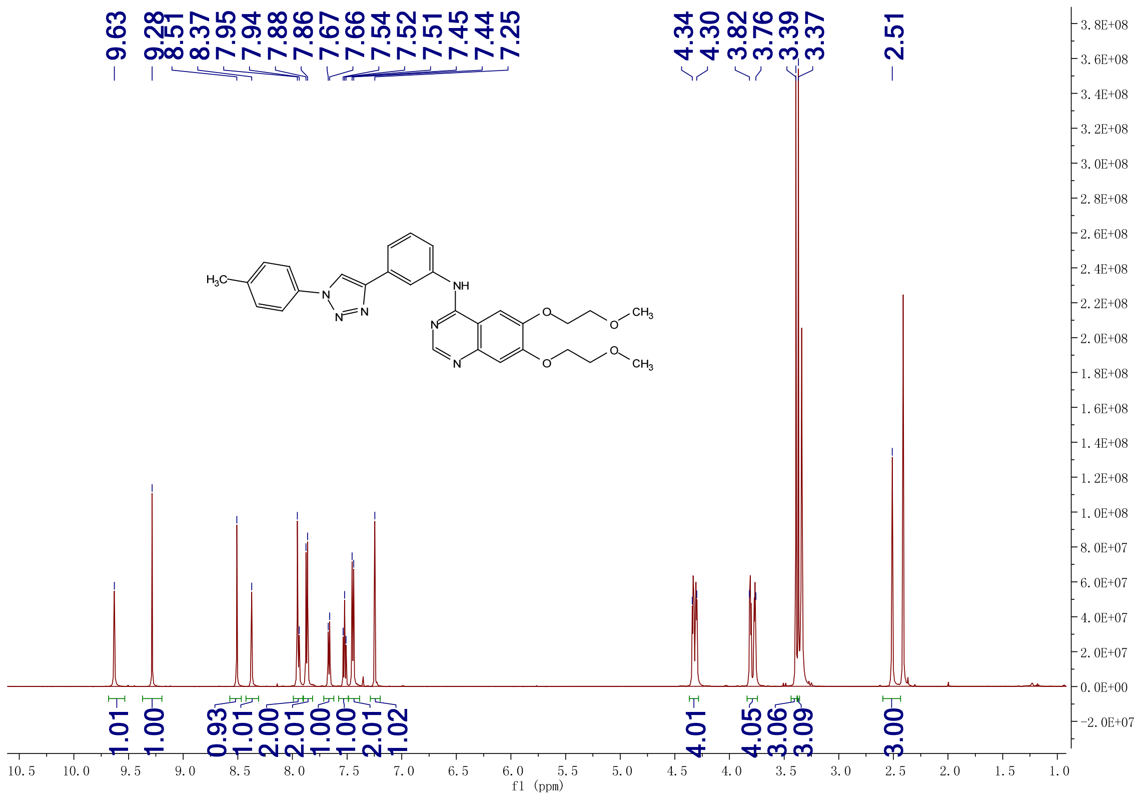

Figure S12-2. ^{12}C NMR spectrum (150MHz, DMSO-d_6) of compound 3l

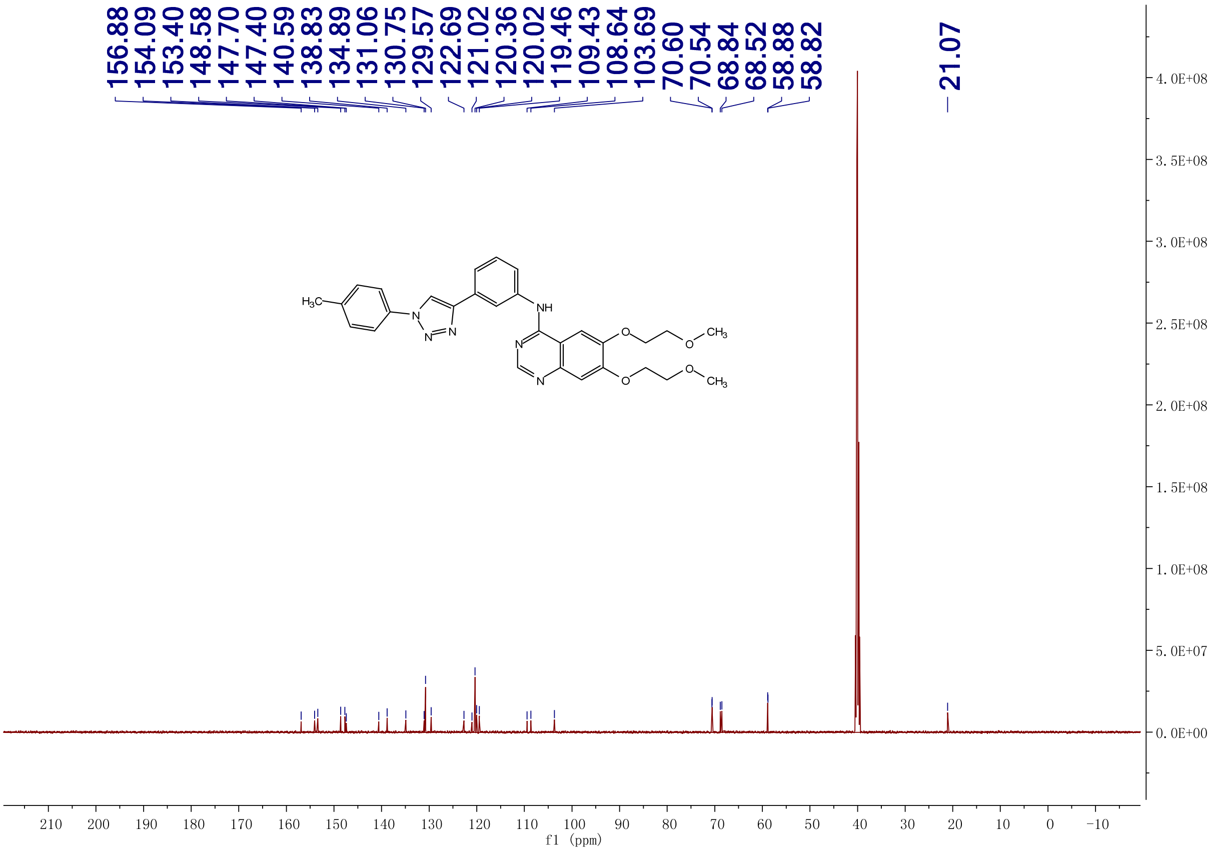

Figure S12-3. HR MS of compound 3l

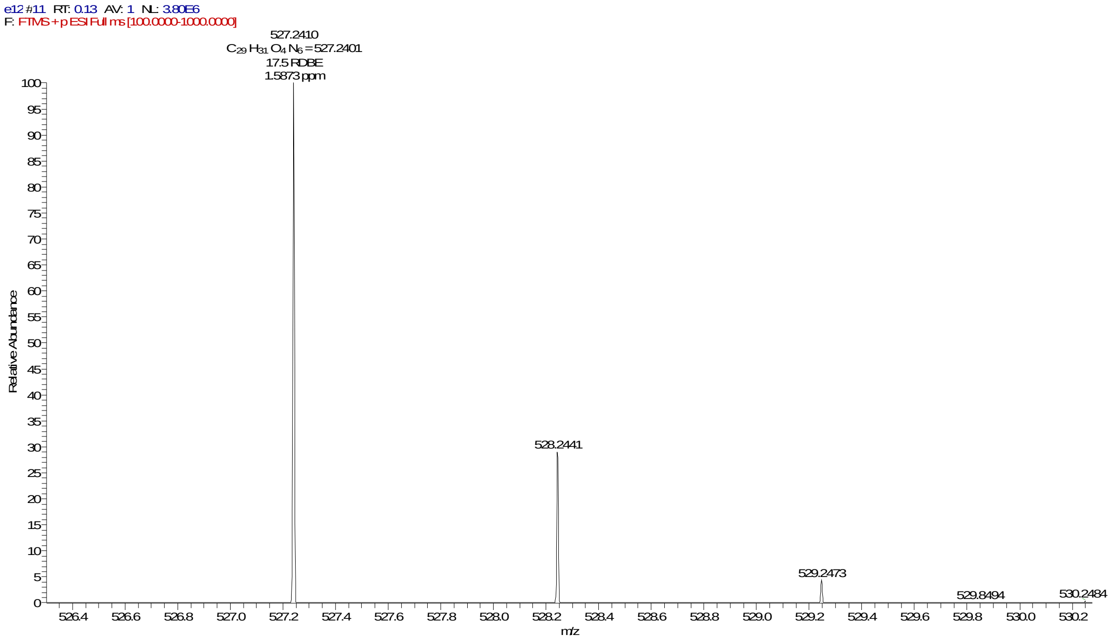

Figure S13-1. ^{1}H NMR spectrum (600MHz, DMSO-d$_6$) of compound 3m

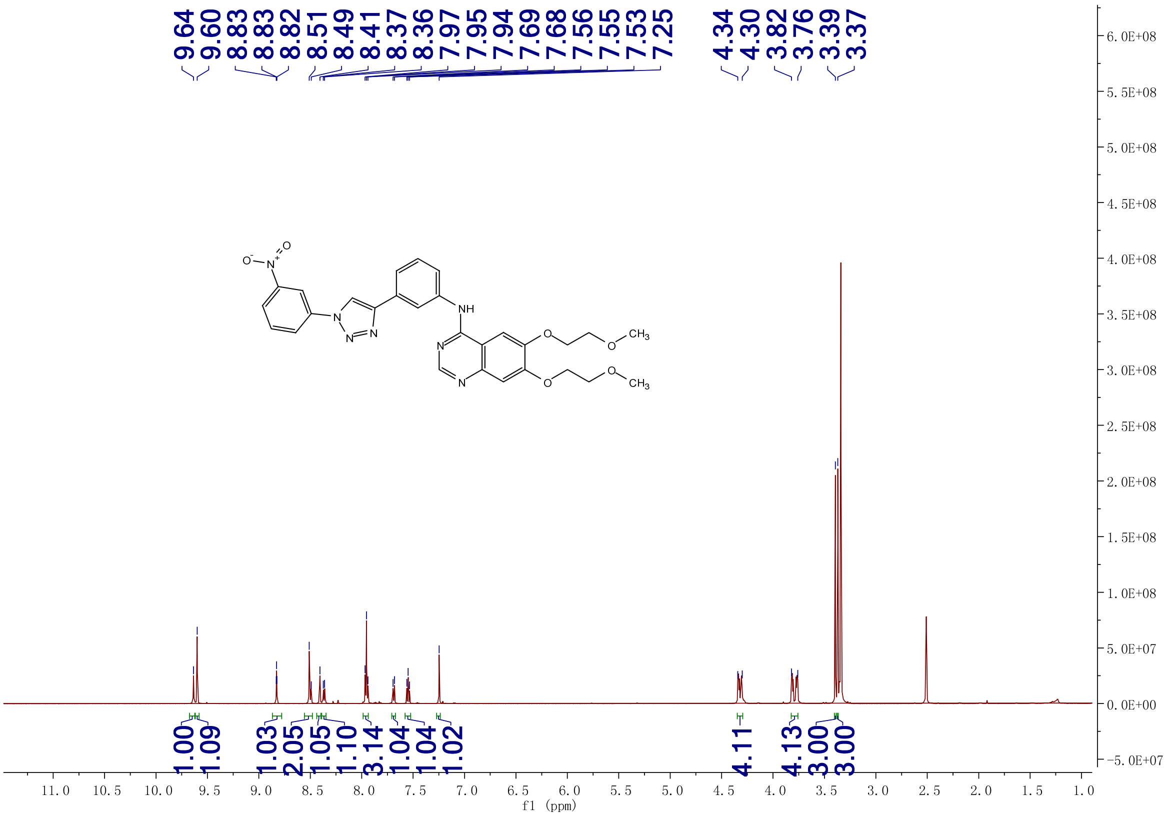

Figure S13-2. ^{13}C NMR spectrum (150MHz, DMSO-d_6) of compound 3m

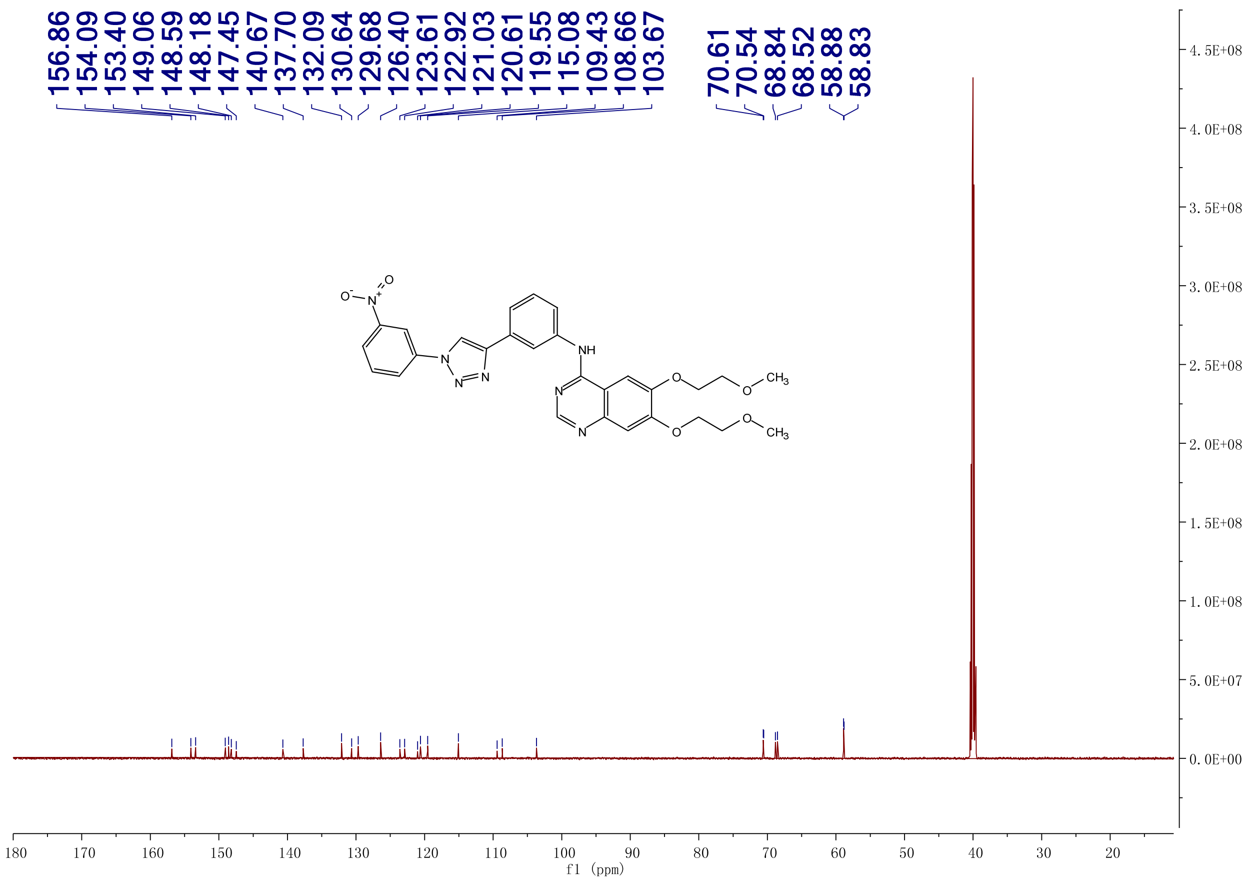

Figure S13-3. HR MS of compound 3m

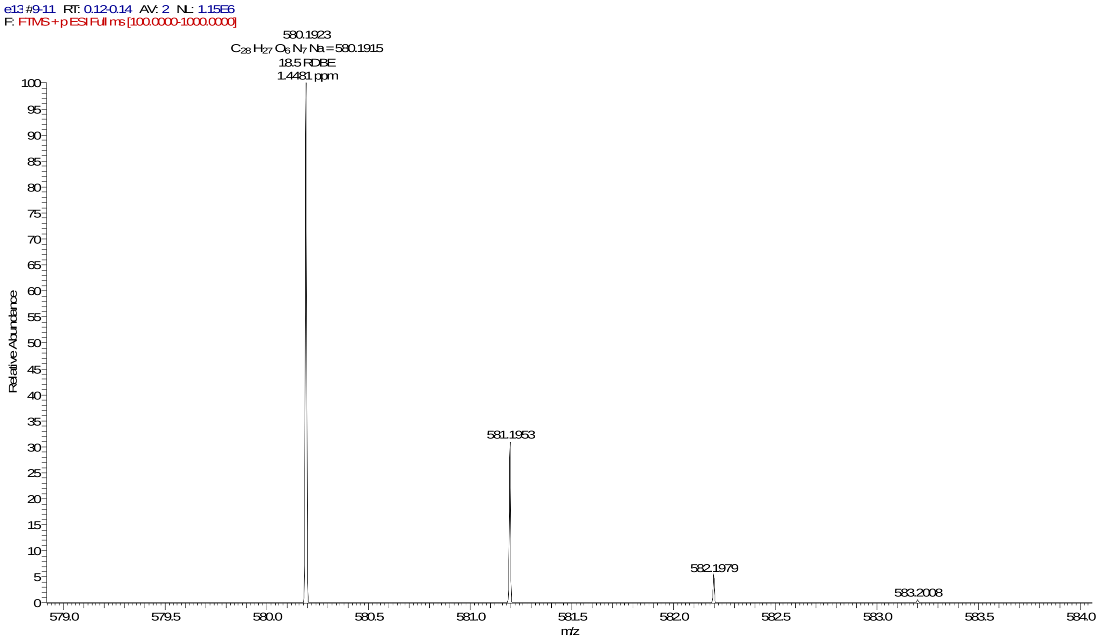

Figure S14-1. ^{1}H NMR spectrum (600MHz, DMSO-d$_6$) of compound 3n

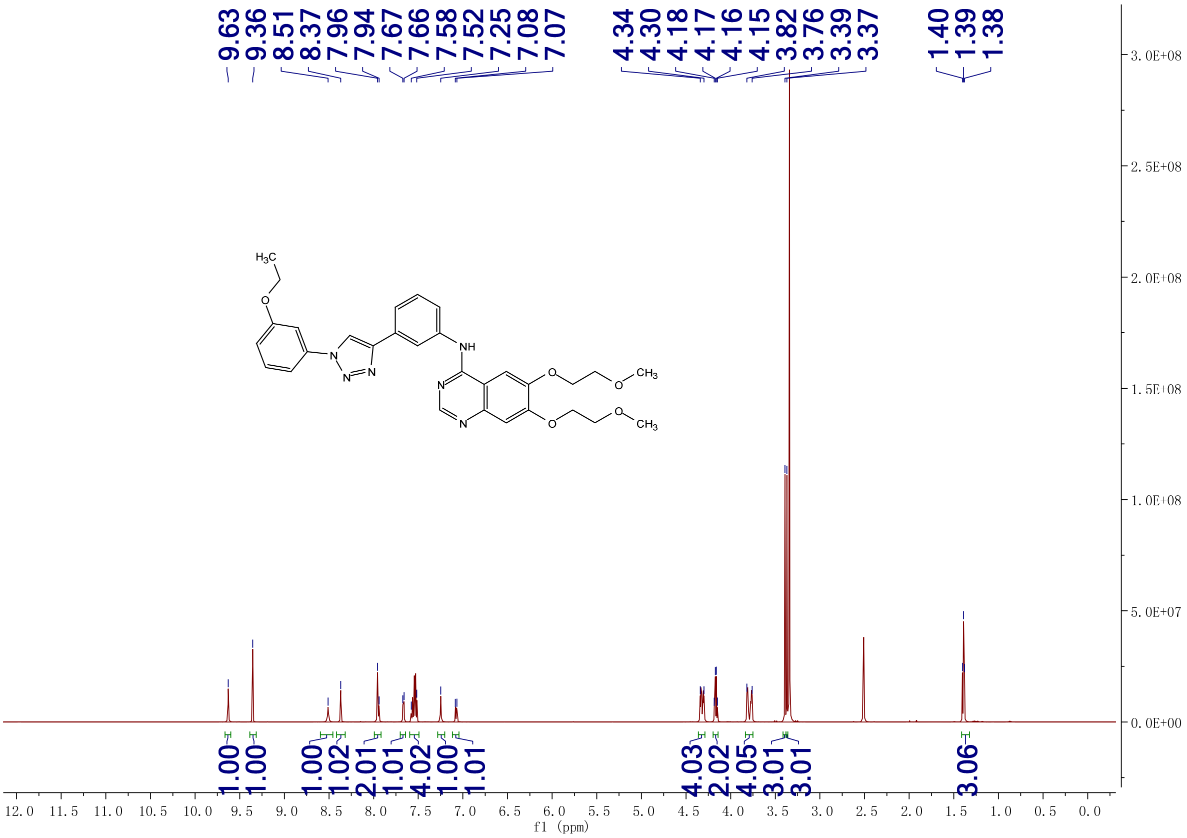

Figure S14-2. ^{13}C NMR spectrum (150MHz, DMSO-d_6) of compound 3n

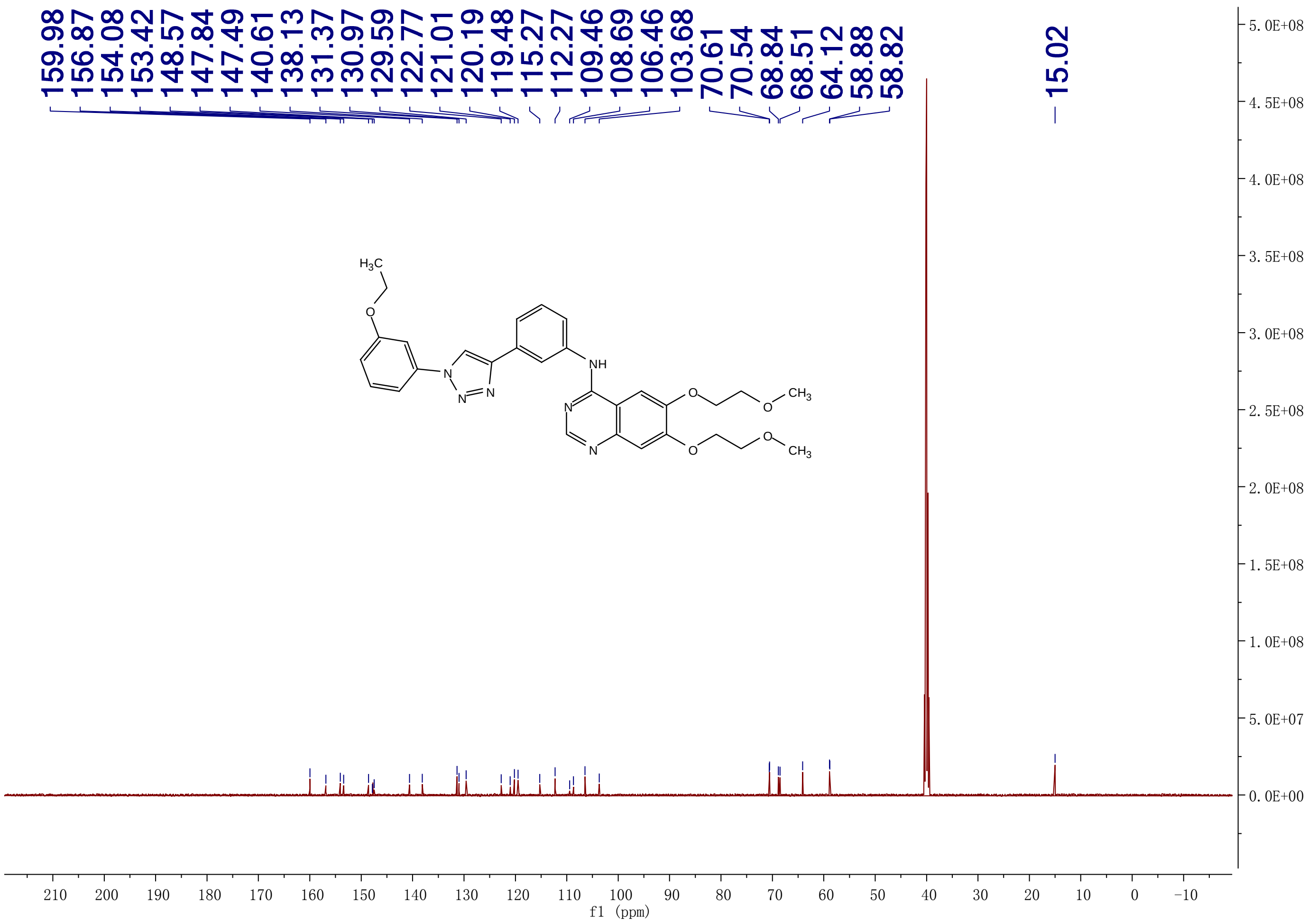

Figure S14-3. HR MS of compound 3n

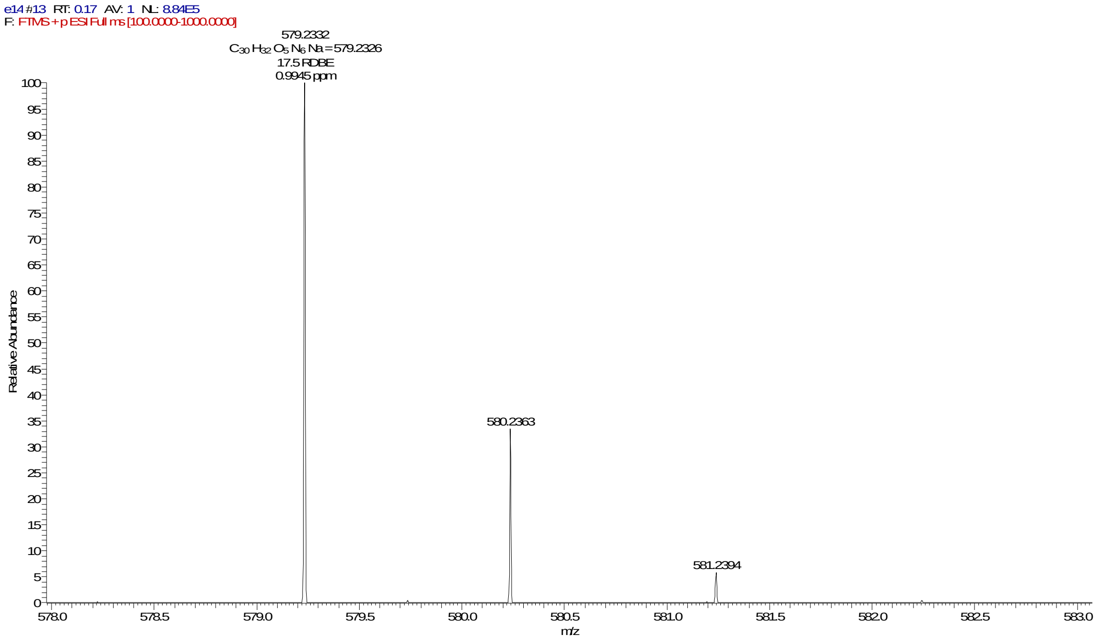

Figure S15-1. ^{1}H NMR spectrum (600MHz, DMSO-d_6) of compound 3o

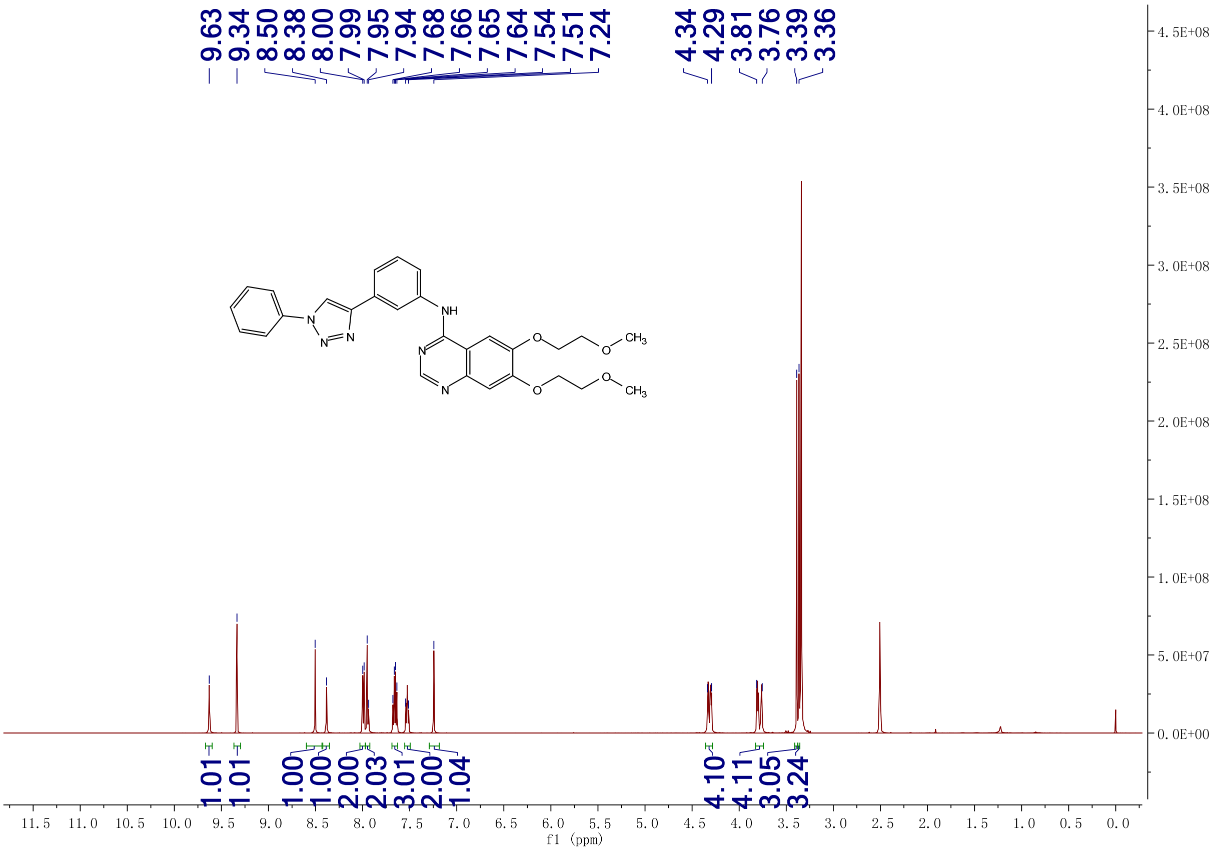

Figure S15-2. ^{13}C NMR spectrum (150MHz, DMSO-d_6) of compound 3o

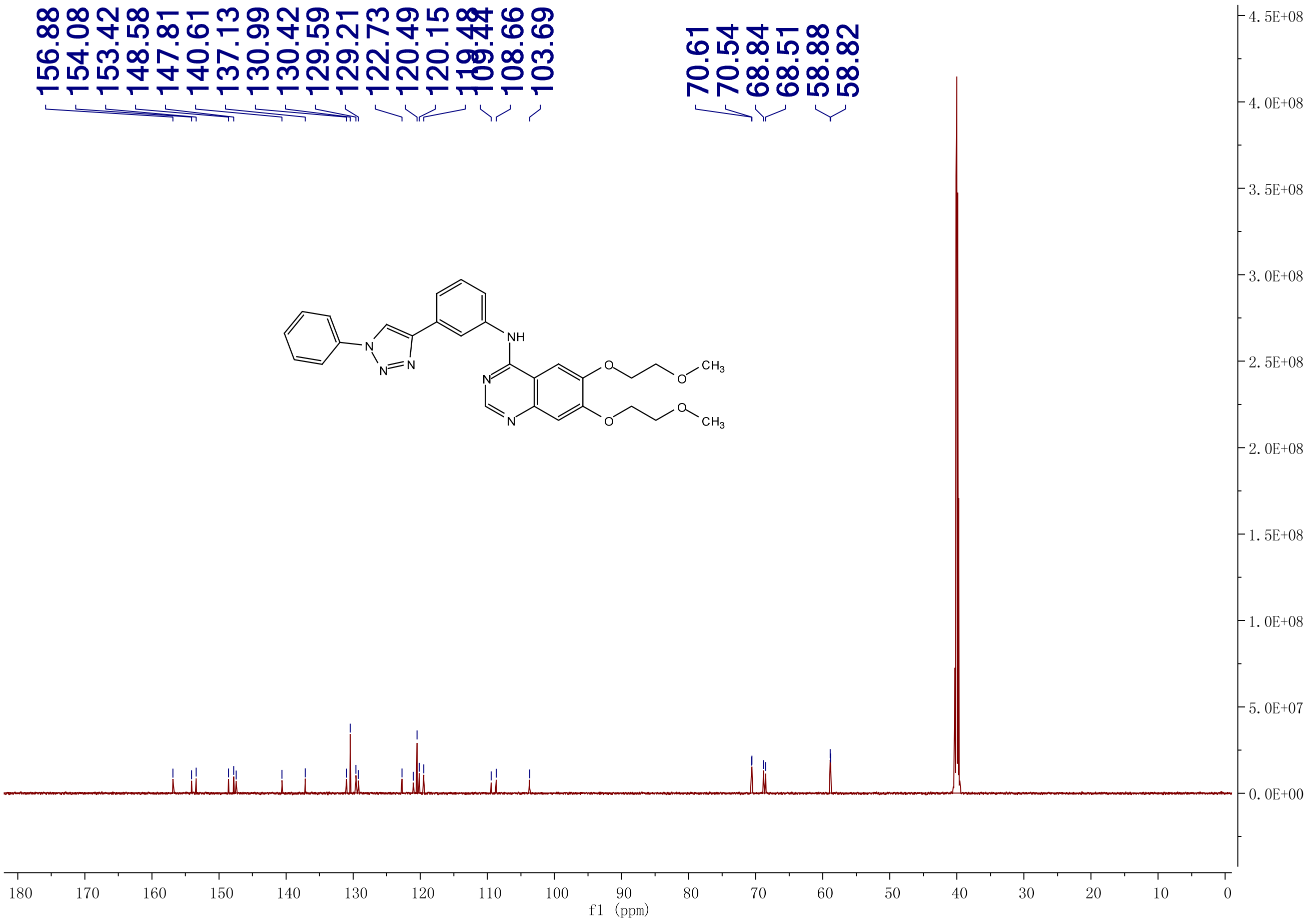

Figure S15-3. HR MS of compound 3o

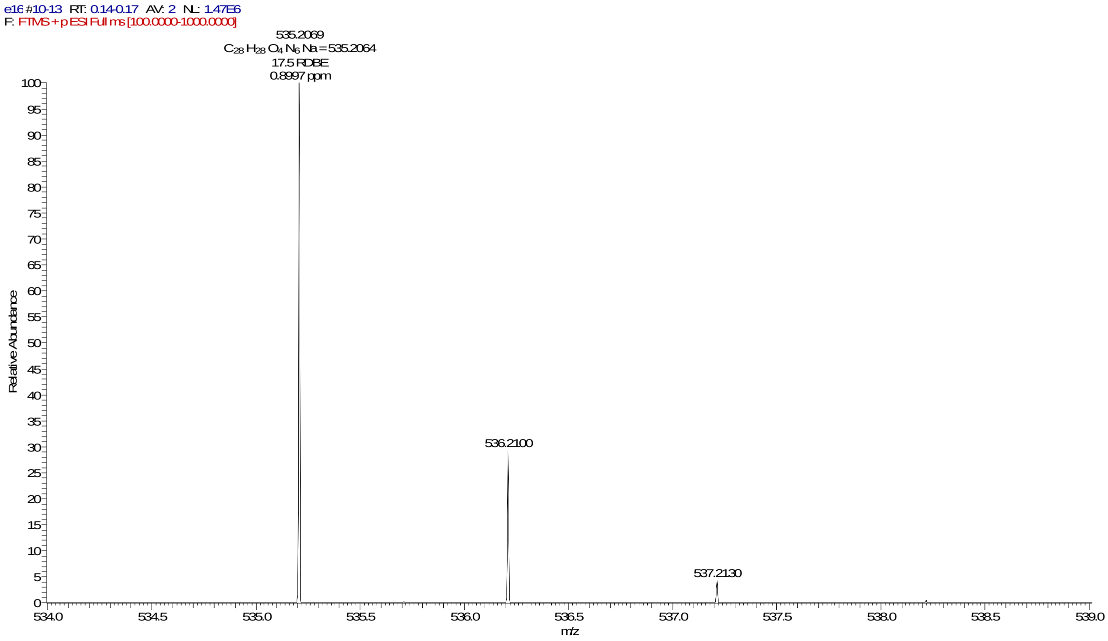

Figure S16-1. ^{1}H NMR spectrum (400MHz, DMSO-d_6) of compound 3p

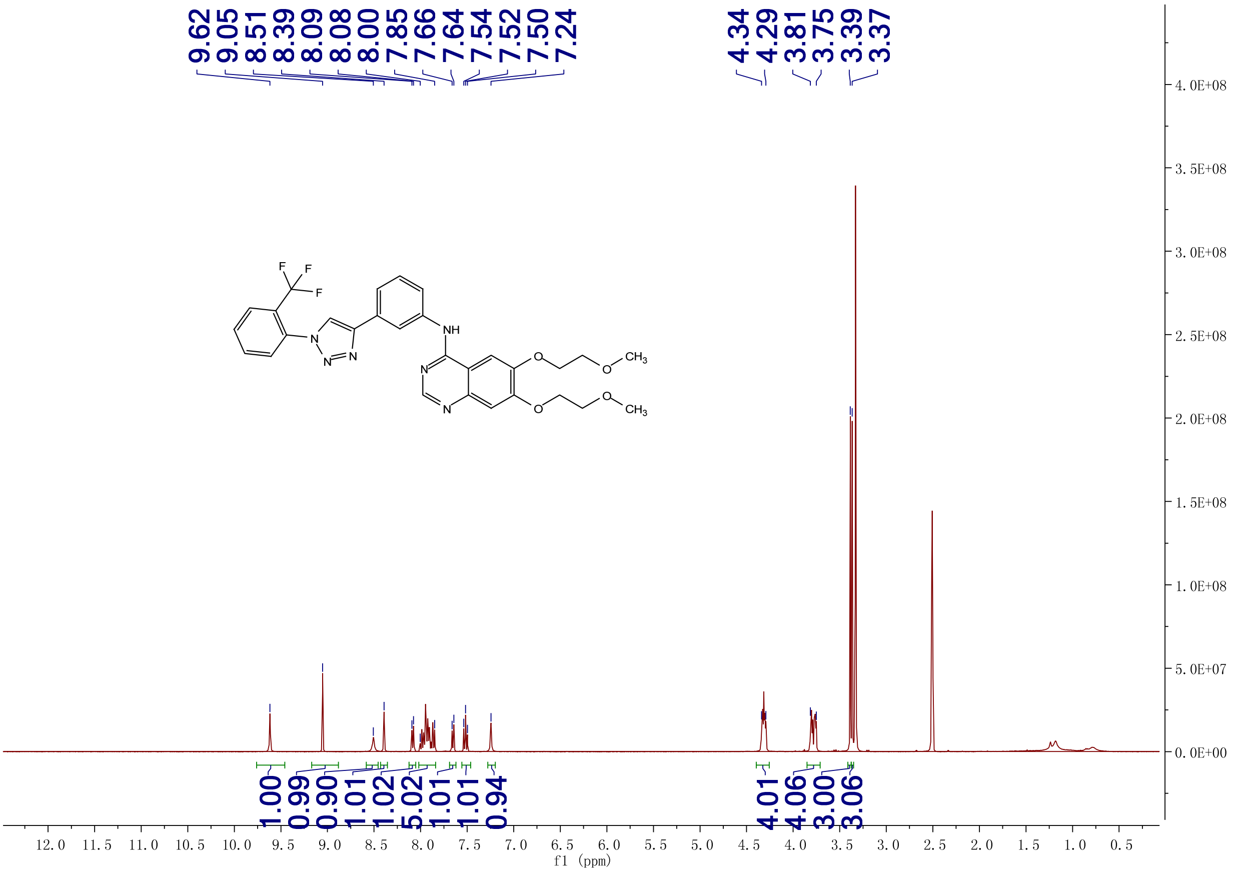

Figure S16-2. ^{13}C NMR spectrum (100MHz, DMSO-d_6) of compound 3p

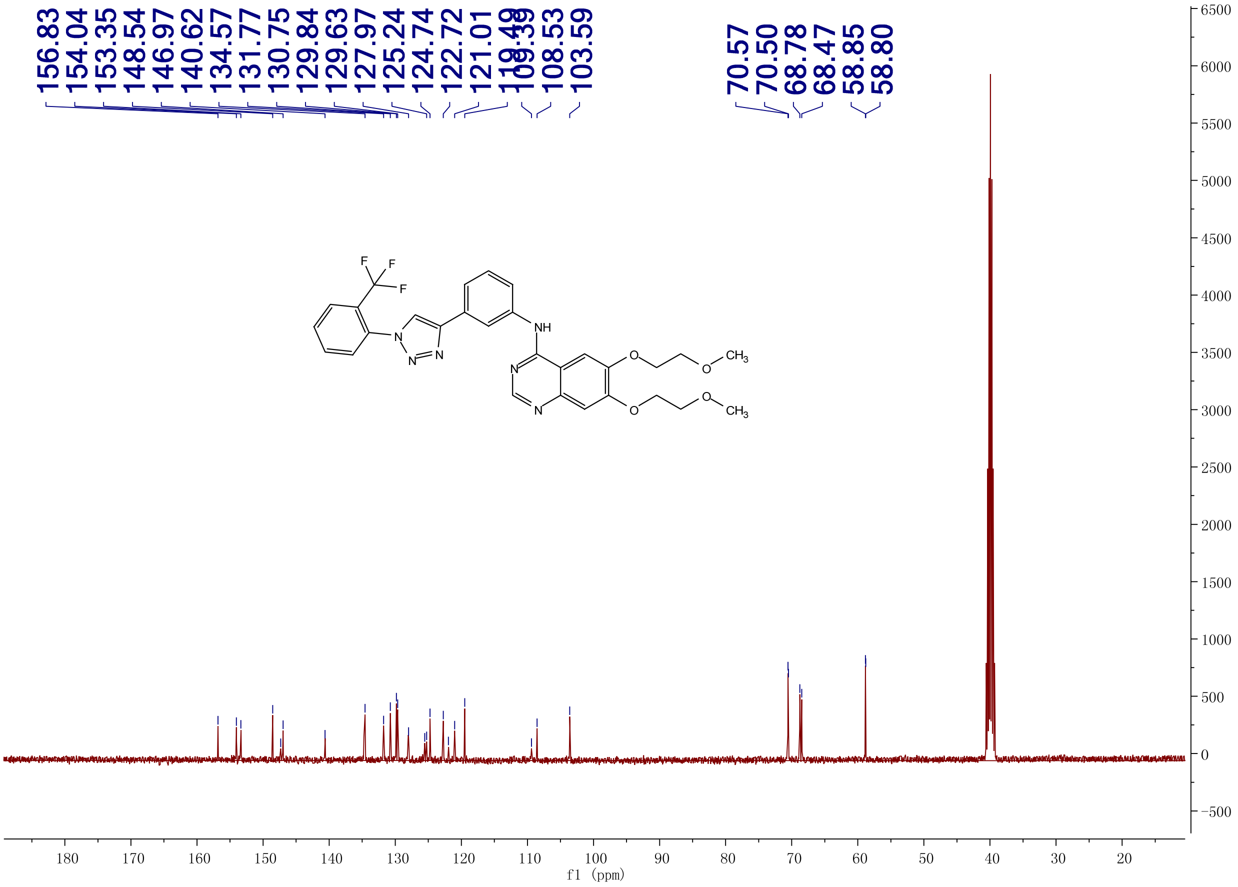

Figure S16-3. HR MS of compound 3p

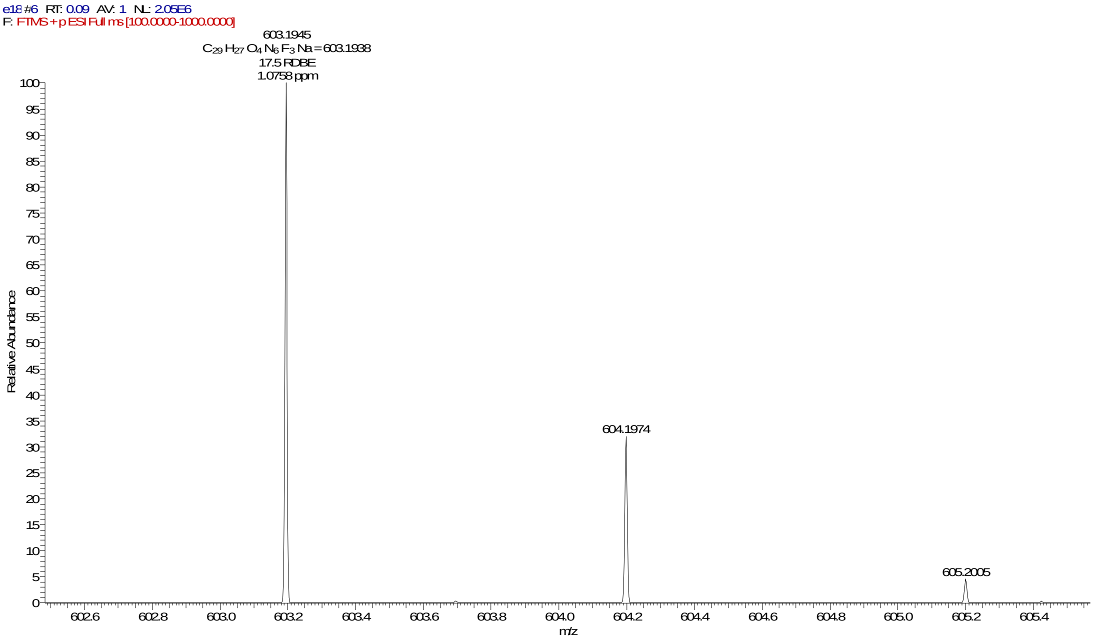

Figure S17-1. ^{1}H NMR spectrum (400MHz, DMSO-d_6) of compound 3q

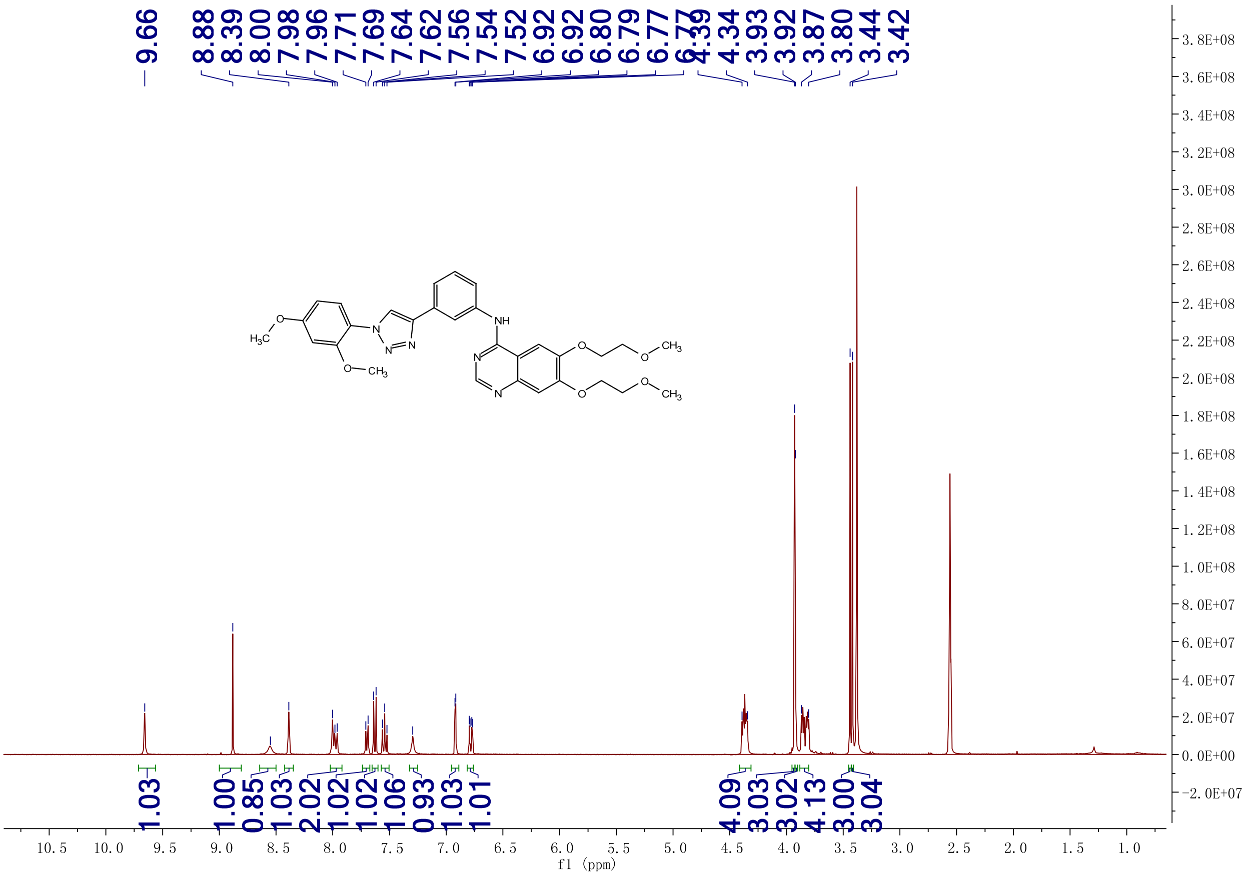

Figure S17-2. ^{13}C NMR spectrum (100MHz, DMSO-d_6) of compound 3q

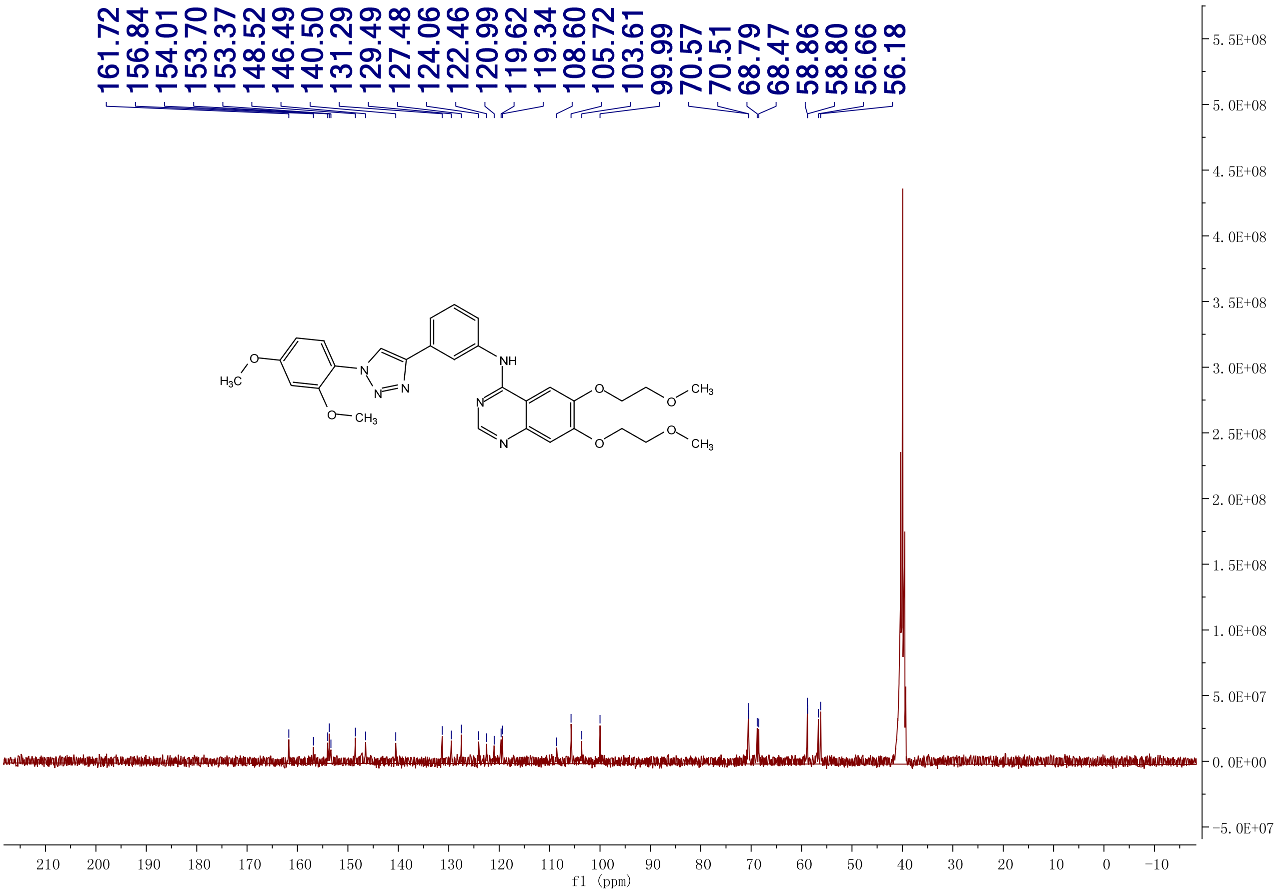

Figure S17-3. HR MS of compound 3q

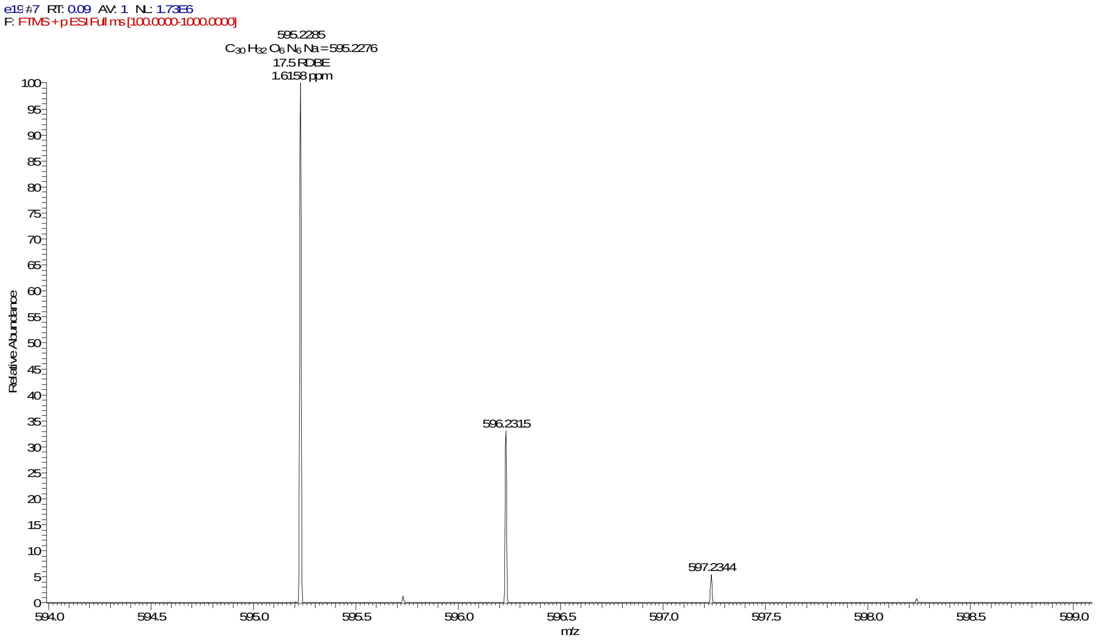

Figure S18-1. ^{1}H NMR spectrum (400MHz, DMSO-d_6) of compound 3r

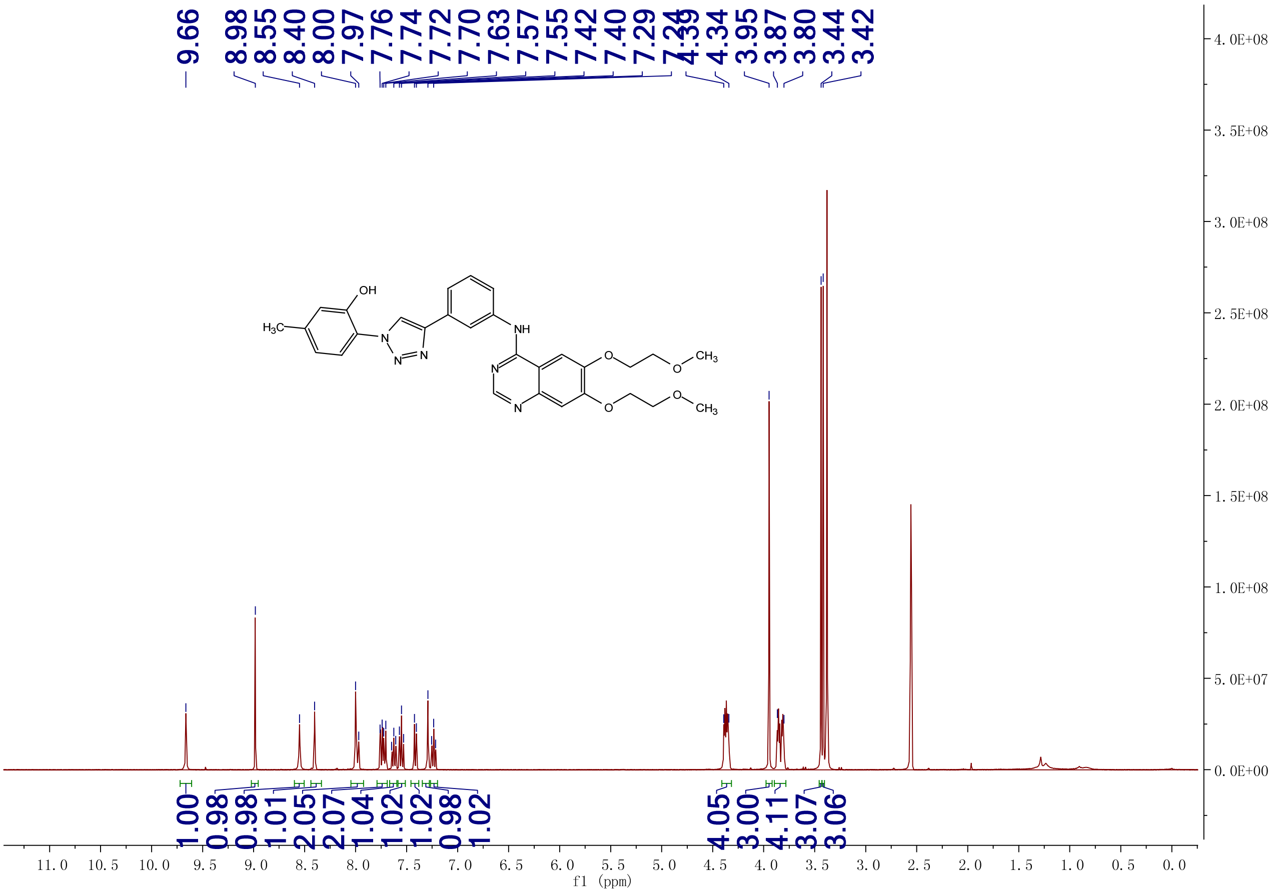

Figure S18-2. ^{13}C NMR spectrum (100MHz, DMSO-d_6) of compound 3r

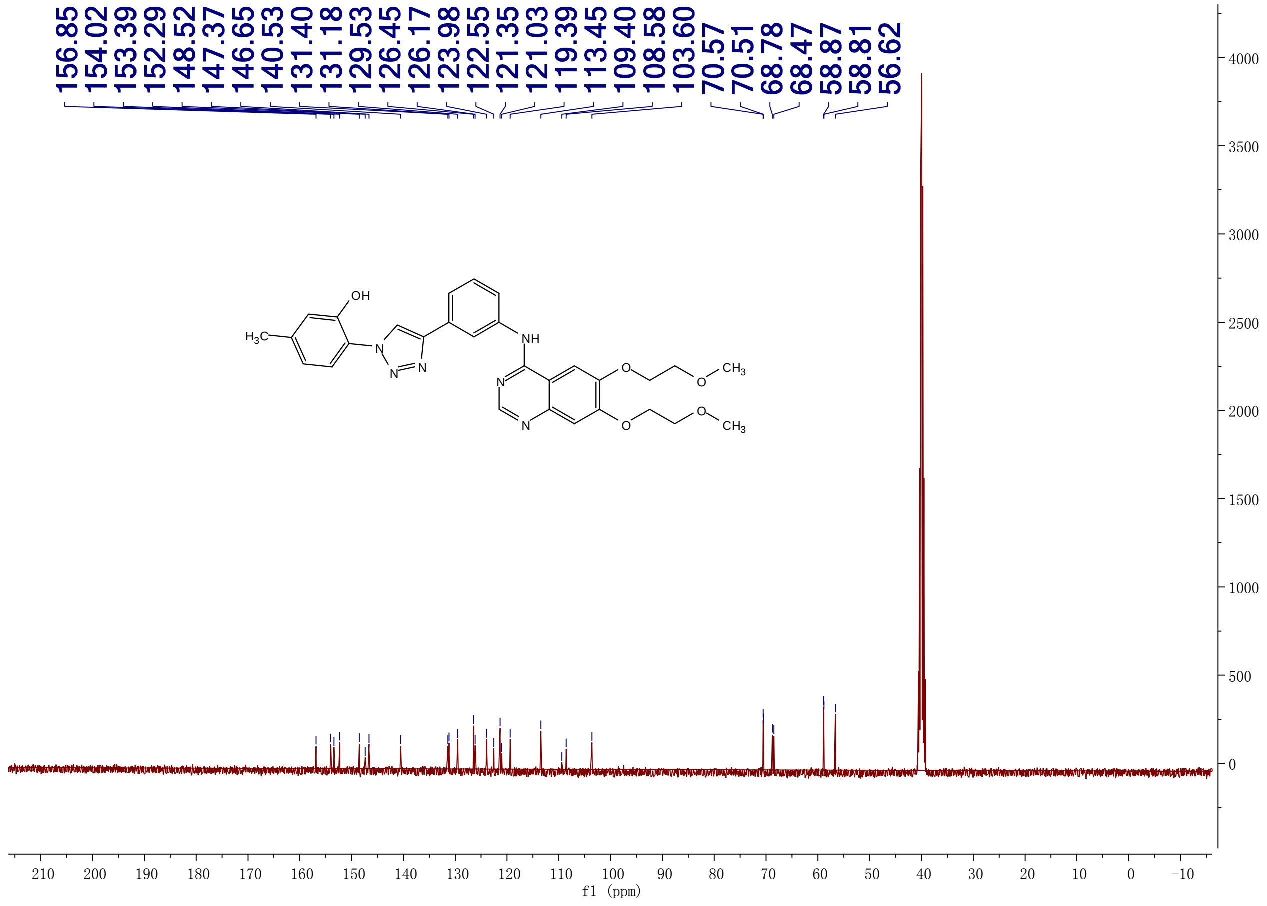

Figure S18-3. HR MS of compound 3r

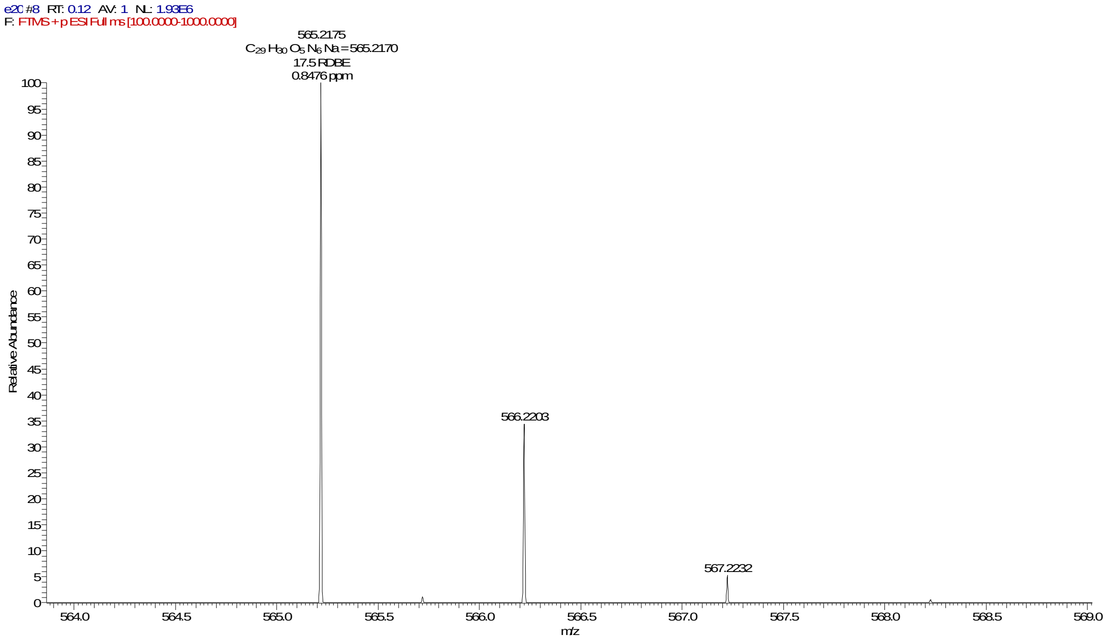

Figure S19-1. ^{1}H NMR spectrum (600MHz, DMSO-d_6) of compound 3s

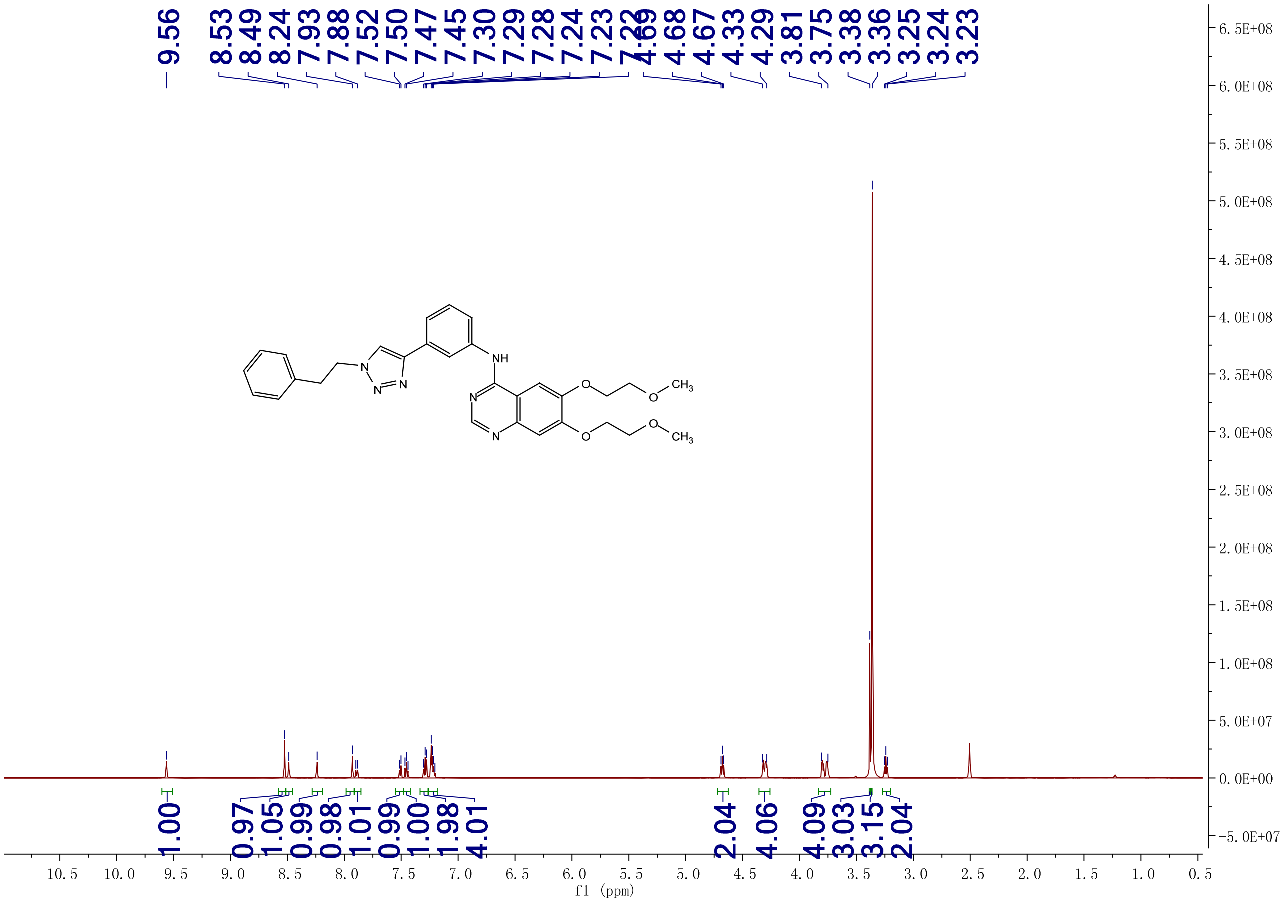

Figure S19-2. ^{13}C NMR spectrum (150MHz, DMSO-d_6) of compound 3s

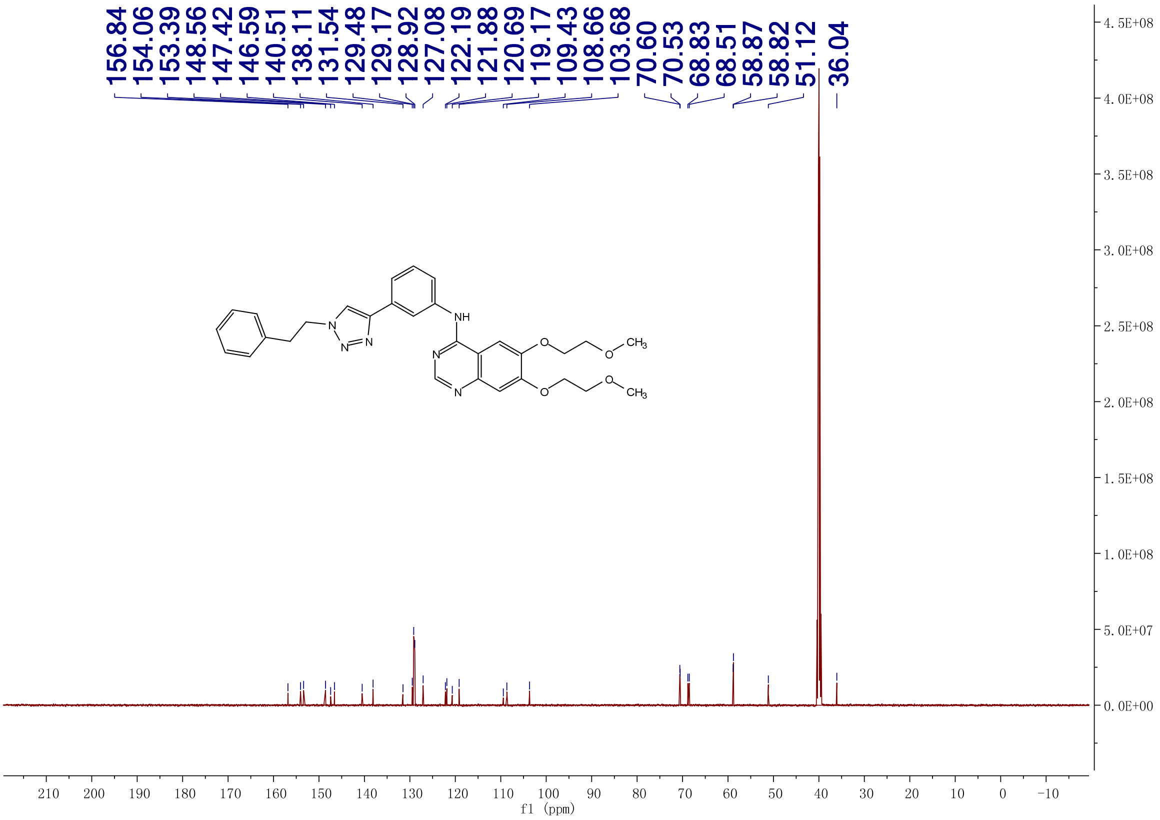

Figure S19-3. HR MS of compound 3s

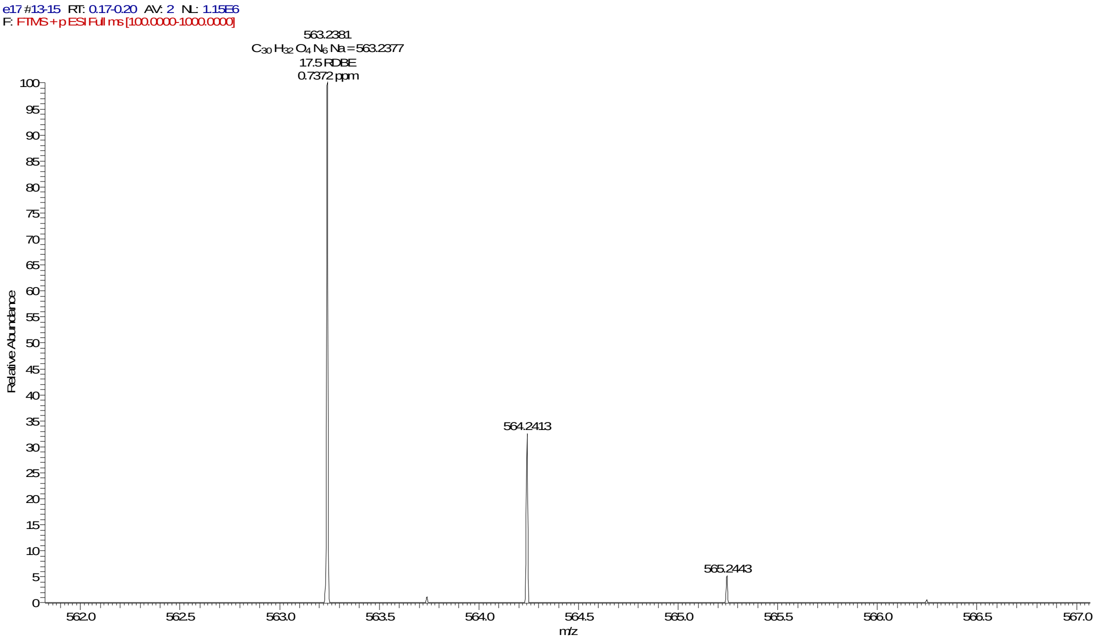

Supplement: Supplementary file 1 [file DataSheet1.pdf]
